# Determinants of tumor immune evasion: the role of T cell exposed motif frequency and mutant amino acid exposure

**DOI:** 10.3389/fimmu.2023.1155679

**Published:** 2023-05-05

**Authors:** E. Jane Homan, Robert D. Bremel

**Affiliations:** ioGenetics LLC, Madison, WI, United States

**Keywords:** immune evasion, neoantigen, T cell exposed motif, T cell epitope, immunoediting, positive selection, microbiome, tumor mutation

## Abstract

Few neoepitopes detected in tumor biopsies are immunogenic. Tumor-specific T cell responses require both the presentation of an epitope that differs from wildtype and the presence of T cells with neoepitope-cognate receptors. We show that mutations detected in tumor biopsies result in an increased frequency of rare amino acid combinations compared to the human proteome and gastrointestinal microorganisms. Mutations in a large data set of oncogene and tumor suppressor gene products were compared to wildtype, and to the count of corresponding amino acid motifs in the human proteome and gastrointestinal microbiome. Mutant amino acids in T cell exposed positions of potential neoepitopes consistently generated amino acid motifs that are less common in both proteome reference datasets. Approximately 10% of the mutant amino acid motifs are absent from the human proteome. Motif frequency does not change when mutants were positioned in the MHC anchor positions hidden from T cell receptors. Analysis of neoepitopes in GBM and LUSC cases showed less common T cell exposed motifs, and HLA binding preferentially placing mutant amino acids in an anchor position for both MHC I and MHC II. Cross-presentation of mutant exposed neoepitopes by MHC I and MHC II was particularly uncommon. Review of a tumor mutation dataset known to generate T cell responses showed immunogenic epitopes were those with mutant amino acids exposed to the T cell receptor and with exposed pentamer motifs present in the human and microbiome reference databases. The study illustrates a previously unrecognized mechanism of tumor immune evasion, as rare T cell exposed motifs produced by mutation are less likely to have cognate T cells in the T cell repertoire. The complex interactions of HLA genotype, binding positions, and mutation specific changes in T cell exposed motif underscore the necessity of evaluating potential neoepitopes in each individual patient.

## 1 Introduction

Recognition of tumor-specific neoepitopes by cytotoxic lymphocytes is the primary immunological mechanism for elimination of tumor cells (1). A fundamental premise is that for an effective tumor recognition response to occur, a mutation must generate an epitope different from the unmutated wildtype (2). Secondly, there must be one or more clones of T cells bearing receptors that bind to the mutant peptide:MHC complex (pMHC). Individual tumor-specific amino acid mutations create unique peptides which are potential targets for neoepitope vaccines (1, 3). However, very few mutations produce immunogenic neoantigens (4, 5). Here we analyze some of the ways in which neoepitopes evade immune surveillance. In particular, we show alterations in the frequency of occurrence of the amino acid motifs exposed to the αβT cell receptor (TCR) by a mutated peptide, when bound and presented by an MHC, compared to the frequency of occurrence of the same amino acid motifs in the human proteome and in a representative gastrointestinal (GI) microbiome. The GI microbiome is included as a recognized source of diverse T cell stimulation linked to cancer outcome (6, 7). As the amino acid combinations that engage the TCR may be continuous pentamers (MHC I) or discontinuous pentamers (MHC II), we refer to these as amino acid “motifs”.

T cell recognition of a tumor-specific mutation depends on presentation of short peptides bound in MHC molecules. Amino acids of the TCR α and β chains engage the MHC histotope and the protruding amino acid side chains of the bound peptides (8–10). T cell recognition is highly polyclonal; the exposed amino acid motif of a bound peptide may be recognized by a hundred or more cognate T cell clones with different alpha and beta subunits (11).The amino acids in a peptide whose side chain atoms interact with those within the MHC groove determine the pMHC binding affinity (12). These groove-facing amino acids in the so-called ‘anchor positions’ are hidden from the TCR. Only amino acid side chains in the non-anchor positions have atomic-level interactions with the TCR (9). Thus, for T cell recognition of a neoepitope, the mutant amino acids must to be in a position exposed to the TCR and not hidden in the anchor positions (13, 14). Both CD8+ and CD4+ T cell responses are needed for an effective tumor targeting response (15–20).

When a peptide is bound in an MHC, whether MHC I or MHC II, the exposed amino acids comprise a pentamer (9, 21–23). We refer to these exposed pentamers as the T cell exposed motif (TCEM) and the hidden residues in the anchor positions as groove-exposed motifs (GEM). In a 9mer peptide bound in an MHC I, the TCEM comprises amino acids p4, p5, p6, p7 and p8 (TCEM I). When a 15mer peptide is bound in an MHC II amino acids p2, p3, p5, p7, and p8 of the central 9mer are the dominant TCEM (TCEM II) (9, 22, 24–27).

The total possible combinations of 20 amino acids as a pentamer is 20^5^ or 3.2 million. We have previously shown that, when all possible sequential peptides are considered, the human proteome only contains approximately 2.4 million of the possible 3.2 million unique pentamers for each MHC class (28). A dataset comprising the proteomes of 67 representative bacterial species found in the gastrointestinal microbiome (GI microbiome) comprised 2.9 million of the possible pentamer motifs (28), partially overlapping those in the human proteome.

Self-peptides in the human proteome are the basis of both positive and negative selection of naïve T cells during thymic processing (29–34). The number of times any self-peptide is presented on thymic epithelial cells, and particularly presentation of the exposed amino acid pentamer motifs they comprise, plays a role in shaping the initial T cell repertoire (35). In early life peptides derived from exogenous proteins, including peptides of the GI microbiome carried by antigen presenting cells, also contribute to positive selection of T cell clones (36–40). The T cell repertoire is further shaped over a lifetime of exposure to peptides with recognized TCEM (41). Prior to puberty and early adulthood this expands the diversity of the repertoire. In later life immunosenescence leads to progressive fragmentation of T cell repertoire diversity, in part due to exposure to chronic viral pathogens (42–45). T cells that recognize uncommon TCEM are less likely to be selected in the thymus, and progressively less likely to be represented in the T cell repertoire as it narrows in ageing, thereby handicapping a response to a low-frequency epitope.

The quorum of T cell clones which can respond to a given pMHC is complemented by T cells which have arisen initially in response to structurally similar “near-neighbor” epitopes (46, 47) and by recognition of more distinct TCEM (23, 48, 49). Limited exposure to rare TCEM in repertoire development would tend to reduce the size of the responding quorum, and likely dampen the overall response rather than eliminate it completely. Not all TCEM will be equally presented to a T cell by individuals of differing HLA genotype. The diversity of peptides presented may be reduced if one or more HLA loci are homozygous (50–53). Intrathymic selection of T cells is thus unique to an individual’s HLA genotype (54). The lifelong sculpting of the T cell repertoire is a function of the combination of peptide binding by individual HLA alleles, diversity of personal antigenic challenge by exogenous epitopes, and TCR cross reactivities (55). While acknowledging these variables affecting the diversity of T cell repertoires, the examination of tumor-specific TCEMs relative to the frequency of matching motifs in the human proteome and other reference datasets can offer insights into the mechanisms of immune evasion. The potential impact of the frequency in the human proteome of pentamers matching TCEM in microorganisms on control of infection has been explored, concluding that the absence of certain TCEM during positive thymic selection may create a disadvantage in immune response to pathogens (35).

Mutations detected in tumor biopsies are the survivors of immune pressure and selection, known as immunoediting, which likely occurs over years before clinical presentation (56–58). Multiple modes of evasion have been described. Many mutated proteins are not expressed and so would be expected to be inconsequential to both tumor cell replicative advantage and immune pressure (4). Peptides comprising mutations, but which are not bound by MHCs, leave potential cognate T cells ignorant of their existence (59). The expression of the HLA loci may be down-regulated in tumor cells (60, 61). The tumor microenvironment may provide physical and immunosuppressive barriers to effective immunogenicity and surveillance (62–68).

In this study we demonstrate the impact of the frequency patterns of TCEM presented to TCR on immune evasion of tumors. Frequency patterns are overlaid on the binding of mutated peptides by MHC, which determines whether a mutant amino acid is exposed or is hidden in a groove facing position.

First, we wished to determine if the TCEM arising from tumor mutations and potentially presented to TCR differ from their un-mutated wildtype homologs when compared to the frequency of matching pentamers in the normal human proteome (human proteome pentamer frequency hPPF) and in the gastrointestinal microbiome (giPPF). This was addressed by examining the pentamer motif frequencies in a large array of recorded missense mutations in 123 proteins previously classified as drivers of tumor progression, either as oncogenes or tumor suppressors (69) and recorded in the Genome Data Commons (GDC) (70).

Secondly, we asked whether mutated amino acids are more or less likely to be hidden from a TCR when bound in the patient’s MHC, and which of those amino acid motifs exposed to the TCR were less common in the reference datasets.

The third question we addressed was whether the patterns of TCEM frequency and HLA binding differed between oncogenes, tumor suppressors, and passenger mutations, and whether the characteristics of the driver mutations or cancer types differed in their ability to escape immune surveillance. These questions can only be addressed within the context of each patient’s HLA genotype and the predicted binding position of each mutant pMHC. To address the above questions, and that of differences between tumor drivers and passengers, we downloaded mutation lists and derived HLA alleles for 31 glioblastoma multiforme (GBM) and 30 lung squamous cell carcinoma (LUSC) cases from the GDC. These two cancers have quite different mutational burdens and a mixture of driver and passenger mutations.

Finally, we accessed a set of tumor mutations previously demonstrated by others to elicit T cell responses and examined which of the criteria of MHC binding and motif frequency they fulfilled (4).

Overall our results shed light on mechanisms of immune evasion and hence guide potential approaches to specifically targeting a tumor cell with an immunotherapeutic approaches.

## 2 Materials and methods

### 2.1 Determination of hPPF and giPPF frequencies

**Figure 1:**
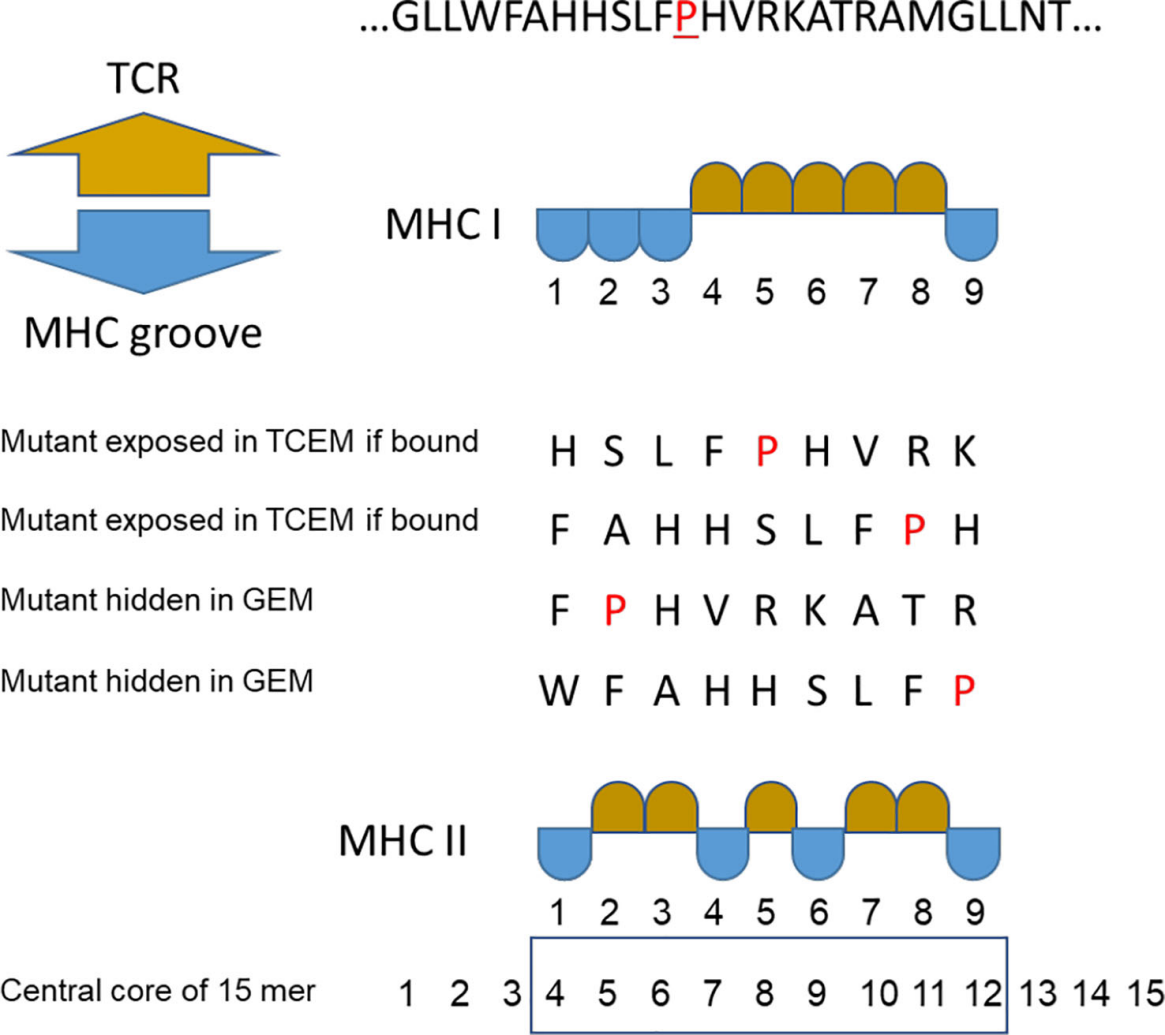
Peptide binding numbering convention. Multiple binding positions in an example peptide where proline (P) is the mutant amino acid. TCEM = T cell exposed motif shown in gold. GEM = MHC groove exposed motif (anchor positions) shown in blue. For MHC II the numbering of the central core of the 15mer is shown.

Amino acid motif frequencies, corresponding to the continuous and discontinuous pentameric configurations of TCEM, were determined in the Hg38 human proteome HUMAN_9606 retrieved from the UniProt repository and excluding immunoglobulins (71). The longest isoform of each protein was selected and used for extraction of the motif frequencies. Each protein in the dataset was broken into successive 15mers with a sliding window displaced by a single amino acid, as previously described (28). This dataset comprised approximately 11.63 million peptides. With this approach the first amino acid of each sequential 15mer corresponds to a linear sequence of the entire proteome. There is a small number of 15mers in the proteome repository that contain unidentified amino acids (X,U,B); peptides comprising these were eliminated. Likewise, the N and C terminal peptides shorter than 9 amino acids were excluded. The first 9 amino acids of each 15mer were used to assemble the database of 9mers comprising the pentamers corresponding to TCEM I at their amino acids p4, p5, p6, p7, p8. These were coded as ~~~XXXXX~, where X is the amino acid in the 9mer sequence and ~ represents any amino acid in the flanking GEM (i.e. anchor) positions. A similar approach was carried out to extract the TCEM II. In this case amino acids p5, p6, p8, p10, p11 of the 15mer represented p2, p3, p5, p7, p8 of the central 9mer which begins at amino acid 4 of the 15mer. TCEM II is coded as XX~X~XX. The amino acid numbering convention we use is shown in **Figure 1**. A master database of each of the possible 20^5^ pentamers was created for each motif configuration and used to determine the frequency distributions of the motifs in the proteome. All pentamer extraction and frequency distributions were done within JMP^®^ datatables, (SAS Institute, Cary N.C). The TCEM I and TCEM II frequency data are count data, where some of the counts are zeros. The best fit of the pentamer frequency distributions were computed as a zero inflated Poisson (ZIP) distribution, where λ is the Poisson mean and π is the portion of the distribution with zero counts in the reference dataset. The JMP^®^ univariate platform provides confidence limit estimates for the Poisson distributions. For some graphical analyses the distribution was standardized using a SHASH transform to normal of the log_2_(1+actual frequency) (72). Because of the underlying Poisson distribution, the SHASH transformed distributions are not typical Gaussian normal distribution curves, but they make the distributional changes easier to discern in graphic presentation.

For the GI microbiome reference dataset the same process was carried out for all open reading frames in the genomes of 67 bacterial species in 35 genera assembled from the NIH Human Microbiome Project Reference Genomes database (www.hmpdacc.org/HMRGD) (28, 73) (**Supplemental Table 1**). The GI microbiome reference dataset is about ten times larger than the human proteome, approximately 109 million peptides.

The result of the above processes is two databases of 3.2 million pentamers each, with tallies of the number of times that a particular motif was present in the human proteome and the GI microbiome. The tallies represent the frequency we refer to throughout the paper as hPPF and giPPF.

### 2.2 Tumor mutation data acquisition

#### 2.2.1 Oncogene and tumor suppressor set

Oncogenes and tumor suppressor genes were identified based on listings by Vogelstein et al. (69). All recorded mutations of 54 oncogenes and 71 tumor suppressor genes were assembled from the GDC in July 2022. Duplicate amino acid mutations created by different codon mutations were excluded. The dataset was limited to missense variants resulting in single amino acid changes. Corresponding wildtype protein sequences were downloaded from UniProt (71), and, based on the genomic annotation convention, the single longest isoform of each protein was used for all computation. Mutant versions of the proteins were assembled and mutation positions of the target amino acids were verified in the protein isoform. Two genes, CDH1 and SMARCA4, were removed from consideration as the reported mutant amino acid changes could not be resolved in any isoform. One reported mutant of ATRX similarly could not be resolved and was excluded. A group of 12 ARID1B mutations were resolved within the shorter reference sequence. The resultant dataset comprised 7,239 mutant protein sequences across 54 oncogenes and 13,634 mutant protein sequences of 69 tumor suppressor genes (**Supplemental Table 2**).

In each mutant and wildtype sequence the amino acid pentamer motifs corresponding to the MHC I configuration of p4, p5, p6, p7, p8 positions, and discontinuous pentamers in the MHC II configuration of p2, p3, p5, p7, p8 were identified and the count of the corresponding motifs determined in the human proteome and GI microbiome datasets. The TCEM comprising mutant amino acids were identified for comparison of frequency with their wildtype counterparts. The mutant proteins were aligned relative to the mutant position (set at zero as shown in **Supplemental Figure 1**) to facilitate graphical comparison of the TCEM hPPF. This process was repeated for the giPPF.

#### 2.2.2 GBM and LUSC sets

Data for 31 cases of glioblastoma multiforme (GBM) and 30 cases of lung squamous cell carcinoma (LUSC) were downloaded from GDC. TCGA case numbers are listed in **Supplemental Table 3**. The cases were selected at random from those for which BAM files were available. This set included 8,207 proteins with missense mutations, comprising approximately 100,000 peptides carrying a mutant amino acid in overlapping 9mer and 15mers. All missense mutations for each case were assembled and mutated sequences constructed and verified as described above. A portion of each BAM file comprising the sequences chromosome 6 were downloaded as the basis for determining patient HLAs. Predicted HLA binding affinity (as described below) was computed as a mean LN(ic50) of 25 member neural network ensembles for each allele in a patient’s genotype. The standard deviation of the ensemble predictions is also computed, providing an estimate of confidence limits around the mean. Kurtosis and skew of binding affinity distributions varies widely among the different alleles. To accommodate these characteristics the ensemble predictions were placed on a common Zscale by a SHASH transformation to normal standardization to zero mean unit variance within protein for each allele (74). Proteins identified as oncogenes or tumor suppressor gene products, as above, were designated “drivers”; other proteins were designated as “passengers”.

#### 2.2.3 T cell responder set

Supplementary Tables of the report by Parkhurst et al. (4) were used to construct mutant protein sequences from the GENCODE ENST hg38 reference sequences. As described above for the GBM and LUSC sets, we computed the Ln(ic50) for peptides in the proteins for which we were able to verify the amino acid at the indicated coordinate using the stable GENCODE ENST (two were eliminated as unresolved). Based on the HLAs reported in the study, the predicted binding affinity was computed for each HLA and placed on a common scale by SHASH transformation to normal. The authors had identified peptides ranging from 8-12 amino acids as the “predicted minimal epitope” that had generated the CD4+ and/or CD8+ T cell peptide recall responses. As our prediction system is restricted to 9mers for MHC I the 12mers were decomposed into four successive 9mers. All the peptides in the author-selected set exhibited a relatively high binding affinity for several 9mer TCEM I binding registers. A 6(allele) x 5(TCEM pentamer) matrix was used to compute the minimum Zscale (highest predicted affinity) across the 6 MHC I genotype and the 5 TCEM I binding registers. The average TCEM I Zscale minimum across all neoantigens in the set was -2.26σ (stdev ± 0.78), indicating the authors had selected high affinity peptides. A composite MHC I and MHC II HLA genotype was created for each patient. We extracted the TCEM I and TCEM II amino acid patterns from the peptide sequences and combined them with the SHASH standardized within-protein predicted binding data.

### 2.3 HLA binding predictions

The approach to predicting binding behavior of peptide-MHC (pMHC) complexes was described previously (75, 76). This is based on a neural network application derived from the chemometric approach of Wold et al. (77) who used partial least squared regression (PLS) of the principal components of amino acid physical properties as predictors of the structure activity relationships of peptides. Use of principal component analysis produces appropriately weighted predictors as input parameters; a key feature in machine learning (78). The principal components used as inputs to the neural networks are effectively dimensionless proxies comprising a large number of amino acid physical properties.

As training sets we use curated public datasets of ic50 (nM) pMHC retrieved from the IEDB repository (79) (minimum 200 pMHC trainers per allele) and only 9mer and 15mer peptides. Neural network ensembles were created with the neural platform of JMP^®^ in a bootstrap aggregating (bagging) process (80, 81). This current approach builds on our initial work, but now uses a much larger, periodically updated, set of data as pMHC training sets (75, 76). The bagging process produces several different predictors from the same training set. The predictors converge to unique solutions that are statistically equivalent, due to the randomization of the trainers by the bagging process. The prediction equations that exhibit the best generalization performance, as determined by four different statistical measures during the training, are used to create an ensemble of neural networks. In practice we generate 300 unique predictors for each HLA allele and then use the best 25 statistical generalizations as the working ensemble set based on the training statistics. Use of multiple ensemble predictions on an individual peptide enables prediction of an ensemble mean and an ensemble standard deviation for any peptide. The standard deviation provides a metric of the precision and a statistical confidence limit of the predictions outside of the training sets used to create the ensembles.

As is common practice in ligand binding analysis, the ic50 input parameter is natural logarithm (Ln) transformed to reduce the bias in the least squares processes introduced by the thousand-fold range in ic50 nM values. As described previously (74) the raw binding data (Ln(ic50)) is standardized to a zero mean, unit variance (ZScale) distribution within each protein using the SHASH or Johnson distribution transformation in JMP^®^. This process places the predictions for different alleles across a genotype on a common scale for the protein under consideration and was shown to be an effective way of analyzing binding and biological activity (75, 76). The underlying concept is that the A alleles will compete with other A alleles, the B alleles with other B alleles, and the C alleles with other C alleles. It is not uncommon within large sets of peptides to find some which are predicted to bind with similar affinities to alleles of several different loci. As TCR tend to use different Valpha and Vbeta families for different MHC this suggests that the same peptide may be presented to different cognate T cells sets in the context of the histotope of the different MHC molecules.

### 2.4 Determination of the HLA genotype

A chromosome 6 BAM slice containing the HLA locus was retrieved from the normal exome files for each GBM and LUSC patient at GDC and converted into a paired fastq files using SamToFastq from Genome Analysis Toolkit (82, 83). The HLA genotypes were determined by tabulating the alignments to exon 2 and exon 3 of different HLA molecules using magicBLAST (84). These two exons comprise the peptide binding domains of the MHC sequences. HLA cDNA coding sequences were retrieved from https://www.ebi.ac.uk/ipd/imgt/hla/ and used to create the BLAST database reference used by magicBLAST.

### 2.5 Combined patterns of TCEM and binding

Whether or not a cognate T cell receptor engages a particular peptide depends on two factors: a) the binding affinity of the peptide to the MHC; and b) which of the amino acids are protruding from the MHC surface and available for TCR engagement (the TCEM) and which are facing inwards towards the MHC groove (GEM) and not directly accessible to the TCR. To represent the combined logic of these two features for the MHC I A, B and C loci and the tumor-specific mutant amino acids we used a 6-bit binary pattern (one for each of 3 pairs of Class I alleles) with each bit representing the logic of two features: a bit = 1 if the peptide binds *and* the mutant amino acid side chain is protruding in a TCEM; and a bit = 0 if the peptide does not bind *or* if it binds but the mutant amino acid is not protruding, *or* both. Thus, a binding threshold was assigned (as a Zscale cut point) and each mutated and each unmutated (wildtype) peptide in the GBM and LUSC dataset was assigned a 6-bit binary values representing the MHC I loci: one for wildtype and one for the mutant. Therefore, there are 2^6^ different patterns of genotype x binding combinations, and each peptide is assigned one of the 64 patterns. A similar 2 bit representation was applied to the MHC II DRB1 alleles. DP and DQ alleles were not included because the presence of two different alpha and two different beta subunits results in potentially 4 different heterozygous combinations of each and without knowing the combinations *in vivo* it is not possible to derive reliable binding affinity predictions. Using this categorical HLA coding approach we created contingency tables of wildtype vs mutant that identify the subsets of different peptides based on presentation of a neoepitope or not. By standardizing all allele binding to the Z scale the multivariate data from different alleles can be combined into composite HLA genotype variables. For example, one can assign a class I genotype to a 6-bit binary value AABBCC: 000000 = no allele binds, 001000 one of the B alleles binds, 100010 one A and one C allele binds at greater affinity than the threshold. The first MHC I A allele for each patient was designated as A1, the second as A2 etc.

Such coding requires determination of an appropriate cut-off to score binding. It has been common practice to use thresholds for binding such as 50nM and 500nM in analysis of MHC binding. However, reports suggest that even peptides with significantly lower affinities are capable of generating T cell responses (2, 13, 59). We previously compared the intra-protein standardized binding for over 400 of different allele + pMHC molecules for which there were curated responses in the IEDB database and in that study found T cell responses to lower MHC binding affinities (74). We found an overlap between positive and negative responses and derived a statistical cut point from a random partitioning algorithm for intra-protein standardized (ZScale) binding values. Depending on the allele being considered this value equates to about 3-5 micromolar, or an affinity about 10x lower than the 500nM value, and more in accord with that reported by Duan et al. (2) and more recently by Yamarkovich (59). Based on this original analysis and validation in multiple infectious disease projects, we routinely apply -1σ standard deviation Zscale value (-1σ) as a cutoff, which corresponds to approximately the 16^th^ percentile point.

### 2.6 HLA genotype binding simulation in the human proteome

To better understand the multi-allelic binding patterns we computed the binding affinities (as LN(ic50) for all 11.6 million peptides in the human proteome described above for a simulated genotype comprising a combination of common HLA alleles: A*02:01, A*03:01, B*07:02, B*08:01, C*04:01, C*07:02, DRB1*01:01, and DRB1*04:01. Using the ensemble approach described above, predicted binding affinities and standard deviations were computed for this combination of alleles for the entire proteome comprising 11.6 million 9mers and 15mers. The affinities were also standardized to Zscale by SHASH transform to normal within protein across the entire proteome. This provided a mechanism for determining the number of self-peptides that would be bound individually and in combination across the HLA genotype for the entire proteome. The Shannon entropy of the HLA binding patterns describes the diversity of binding for any particular one of the GBM and LUSC cases.

## 3 Results

### 3.1 Human proteome peptides bind to common MHC genotypes

**Figure 2:**
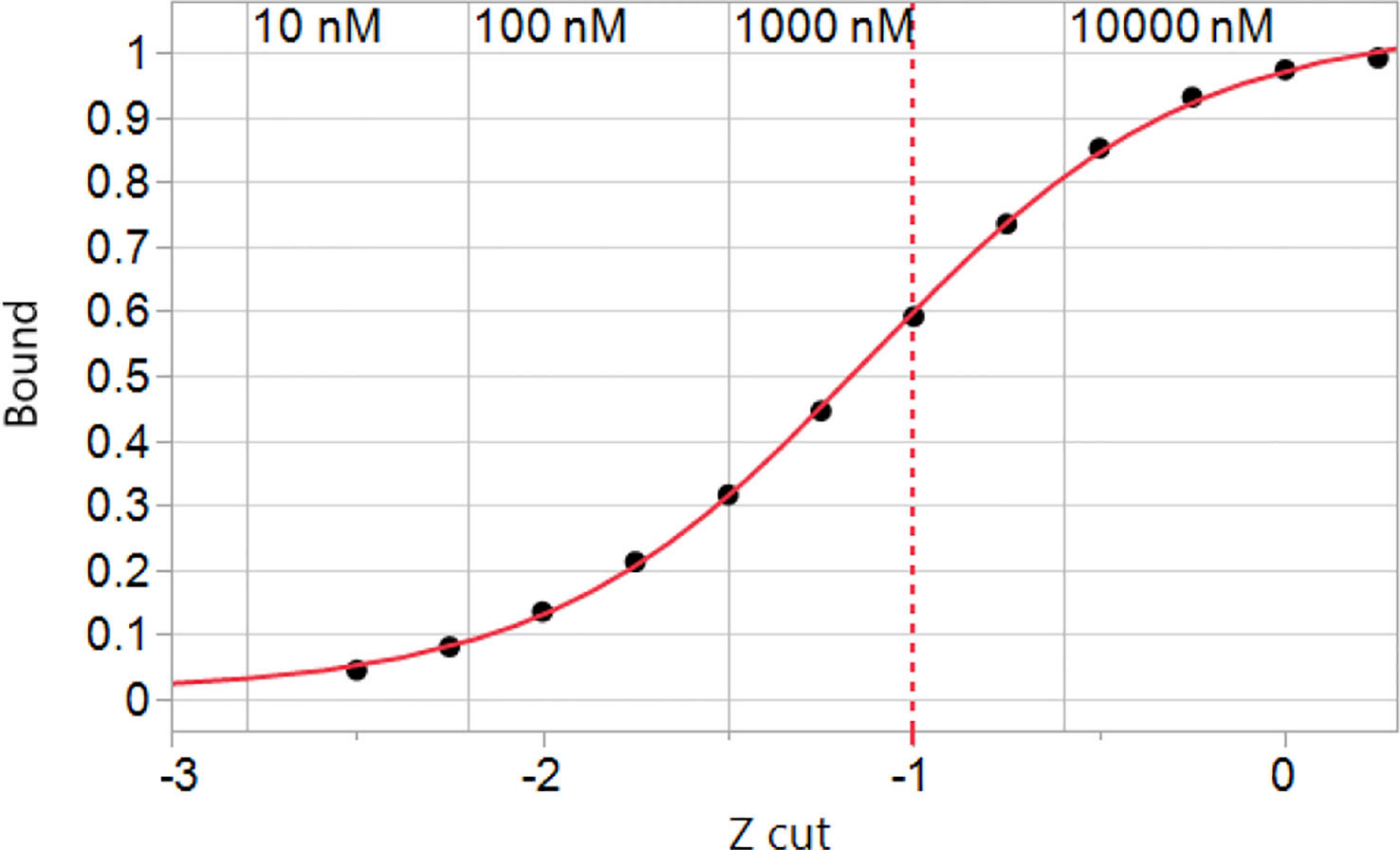
Simulated binding of peptides in the human proteome for a hypothetical genotype of common MHC I alleles. The cumulative peptide count was tallied at each of the Zscale cut-points of the standardized binding affinity and the fraction of the proteome at that standardized affinity is plotted vs. the Zscale cut point (**Supplemental Table 4**). Numerical values of the predicted ic50 in nM are indicated for A*02:01 back-calculated from the standardized values. The values of other alleles is similar. The inflection point of the curve with a 4 parameter logistic fit is -1.12σ. A -1σ cut point indicated by the dashed line is used throughout.

Using the Zscale standardization it was possible to derive a picture of how a typical HLA genotype responds to the entire human proteome. Binding of the peptides by the constituent HLAs exhibits a sigmoid curve to which a standard 4 parameter logistic plot of a Hill coefficient model can be fitted (**Figure 2**). Interestingly, the inflection point of the curve is at a Zscale value of -1.12σ for A*02:01. The other alleles have similar values, supporting the use of -1σ as a convenient and reasonable approximation of the half-maximal binding point for an entire genotype, and concordant with our previous results with T cell epitope responses to influenza (74). The -1σ Zscale metric is effectively a surrogate for the Kd commonly used in receptor analysis. It should be noted that the Zscale sigmoid curve midpoint is consistent with the binding affinity described by Duan et al. (2). Proteome-wide peptide binding patterns for this HLA genotype are summarized in **Supplemental Table 4**.

### 3.2 T cell exposed motif frequencies in oncogenes and tumor suppressor gene products

#### 3.2.1 Tumor mutations generate less common T cell exposed motifs

**Figure 3:**
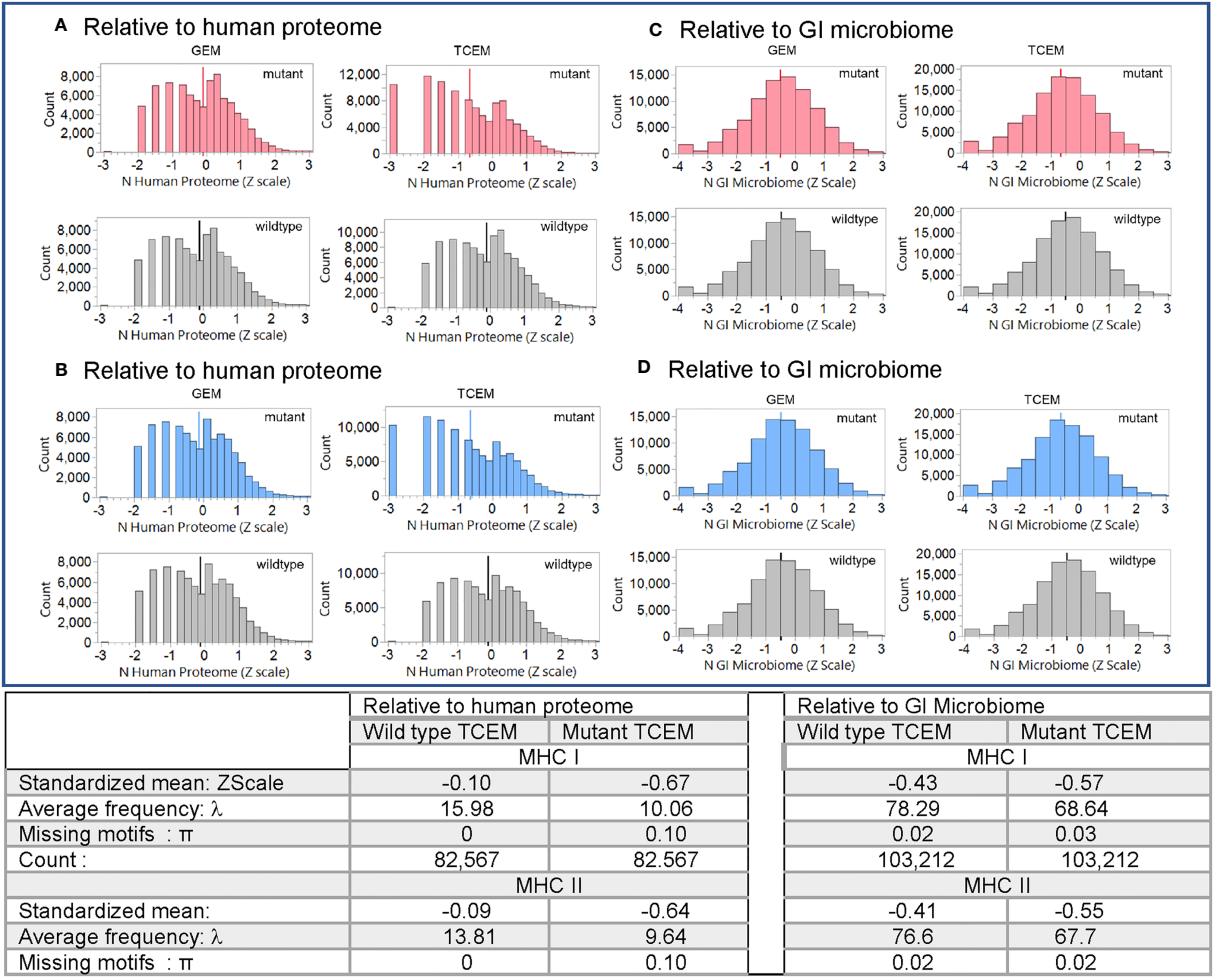
Pentamer motif frequencies comprising the mutant amino acid in oncogene and suppressor gene products ranked by matching pentamer frequencies in the human proteome and GI microbiome. The Y axis shows the count of pentamer motifs in the oncogene and suppressor gene product mutation dataset. Counts of pentamer positions which place the mutant amino acid in the MHC I GEM I or TCEM I positions are shown in red. Discontinuous pentamer positions which place the mutant amino acid in the MHC II GEM II or TCEM II positions are shown in blue. Wildtype homologues are shown in grey. In **(A, B)** the X axis is the Z scale standardized frequency of each pentamer motif in the human proteome (hPPF). The histogram bar on the far left of **(A, B)** TCEM indicates motifs absent from the human proteome, the second bar is singletons, the third bar doubletons etc. In **(C, D)** the X axis is the Z scale standardized frequency of each pentamer motif in the GI microbiome (giPPF). This larger dataset more closely approaches a normal distribution, while still underlain by a Poisson distribution. Summary statistics are for the standardized distribution shown and for the corresponding Poisson distribution shown in **Supplemental Figure 2**. λ= Poisson mean, π = fraction of zero counts.

**Figure 4:**
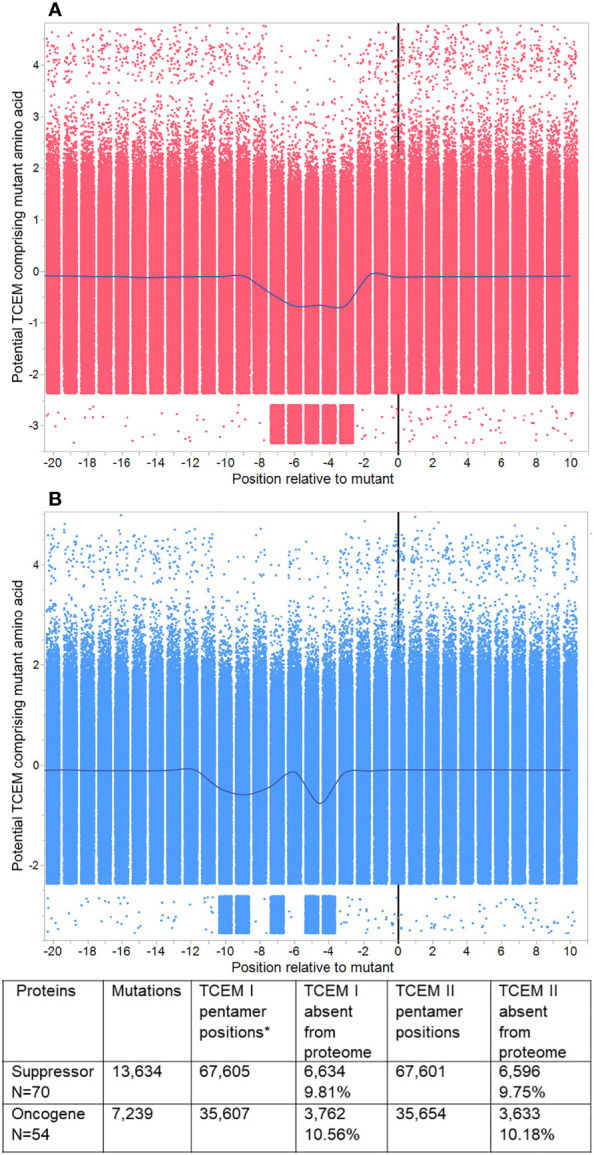
Pentamer motif frequencies in mutated oncogenes and suppressor gene products, aligned at the mutant position. Peptide 9mers adjacent to the mutant position in 123 oncogenes and suppressor gene products were aligned at the mutant position 0. **(A)** shows TCEM I (MHC I). **(B)** shows TCEM II (MHC II). The Y axis shows the Z scale standardized frequency in the human proteome of the pentamer motifs corresponding to the potential TCEM at each position. Overall, each plot shows 679,210 pentamer motifs comprising each mutant amino acid in each possible position for 20,873 unique mutations. The points are randomly jittered for visualization. The line shows the mean at each TCEM position and shows the downward shift in hPPF for those TCEM containing the mutant amino acid. A few rare motifs occur outside the main pattern. These arise from 16 proteins in which the longest isoform was non-canonical, and in which there were single rare motifs at positions other than the mutant which then appears for each of multiple mutants (e.g. RUNX1 isoform Q01196-8 has 84 mutants and a single additional rare motif).

Evaluation of the hPPF and giPPF characteristics of TCEM across the 123 recognized oncogene and suppressor gene products shows that when a mutant amino acid is located in a T cell exposed position, the matching amino acid pentamer motif has a lower frequency count in the human proteome (hPPF score), as shown in the Zscale histogram in **Figure 3**. In approximately 10% of TCEM the corresponding pentamer motif is completely absent from the proteome (**Figures 3A, B** TCEM). To a lesser degree, the same pattern is seen relative to the giPPF (**Figures 3C, D** TCEM). Corresponding Poisson distributions are in **Supplemental Figure 3**. **Supplemental Figure S4** shows the impact on hPPF frequency of the TCEM when the mutant amino acid is located in each pocket position. A peptide which is preferentially bound so that the mutant amino acid lies in a GEM position has with no change in hPPF and giPPF relative to wildtype, however a mutation in the GEM position will likely modify binding as discussed below. As tabulated in **Figure 3**, there was no difference between the patterns observed in oncogenes and in suppressor gene products, so these are combined in both **Figures 3**, **4**.

As shown in the table in **Figure 3** for oncogene and suppressor mutant peptides presented by MHC I, the hPPF of matching pentamers changes from a count of 15.98 in the wildtype with zero missing (hPPF=0) to a hPPF of 10.06 in the mutant, with 10% mutant TCEM having no representation in the normal human proteome. The Poisson summary statistics (λ=mean, π = fraction of zero counts) also shown in **Figure 3** are highly significantly different. The Poisson mean frequencies of the pentamer motifs in the wildtype oncogenes and suppressors are overall about 3 times that in the proteome as a whole, where λ= 4.9. However, as it is a Poisson distribution, while the TCEM hPPF frequencies of the wildtype driver proteins appear quite common in aggregate, there is a wide variance among the individual proteins.

The Poisson mean frequency count in the giPPF (λ = 37.7, compared to 4.9 hPPF) as a whole is higher than the proteome, reflecting the larger size of the GI dataset, which is approximately 10x the size of the human proteome. Just as noted in the hPPF, the TCEM comprising the mutant amino acid in the oncogene and suppressor proteins are also less common in the giPPF; the λ decreases from 78.29 (wildtype) to 68.64 (mutant). There is also a statistically significant increase in the fraction of TCEM absent from the GI microbiome (i.e. giPPF=0). Overall the motifs in the wildtype driver genes are quite common in the giPPF and overlap with those in the hPPF.

For MHC II, the patterns and frequency shifts of the TCEM II motifs parallel those of TCEM I for both reference datasets. In TCEM II, the hPPF λ is decreased from 13.81 in the wildtype to 9.64 in the mutated protein and the giPPF from 76.6 to 67.7. Corresponding Poisson distributions are in **Supplemental Figure 2**.

The entire dataset of mutated protein sequences was aligned by setting the mutant position to zero. **Figure 4** shows the sequential peptides in positions either side of the mutant position, using Z-scale scoring of each TCEM relative to their hPPF. The overall hPPF of the five positions that comprise the mutant amino acid is clearly reduced for both MHC I and MHC II as is shown by the smoothing line through the means at each position relative to the mutation at zero. These patterns are a sliding window of TCEM indexed by one amino acid so the observed concordance of changes is not a gradual change, but an abrupt shift for TCEM at a specific position. Only at the precise position where the mutant amino acid is placed in the TCEM pentamer does the frequency change. Positions either side, where the mutant amino acid would lie in the GEM positions or outside the bound peptide, are unaffected.

Within this aggregate pattern, comprising 20,873 mutations, there are different numbers of unique mutations for each oncogene and suppressor gene product, and different numbers of cases are associated with each mutation. The overall frequency trend is towards a statistically significant lower hPPF (seen also in **Figures 3A, B**). Any single missense mutation may create from zero to ten TCEM motifs (TCEM I plus TCEM II) that are completely absent from the human proteome. There is a very small subset of mutations within the set that produce TCEM for which the frequency increases.

#### 3.2.2 Example proteins

The oncogene and suppressor gene product dataset includes 471 unique mutations in TP53 representing 3,147 cases documented at GDC. TP53 stands out among the proteins analyzed as having not only a large number of different mutations, but also proportionally a high count of low-frequency TCEM.

**Figure 5:**
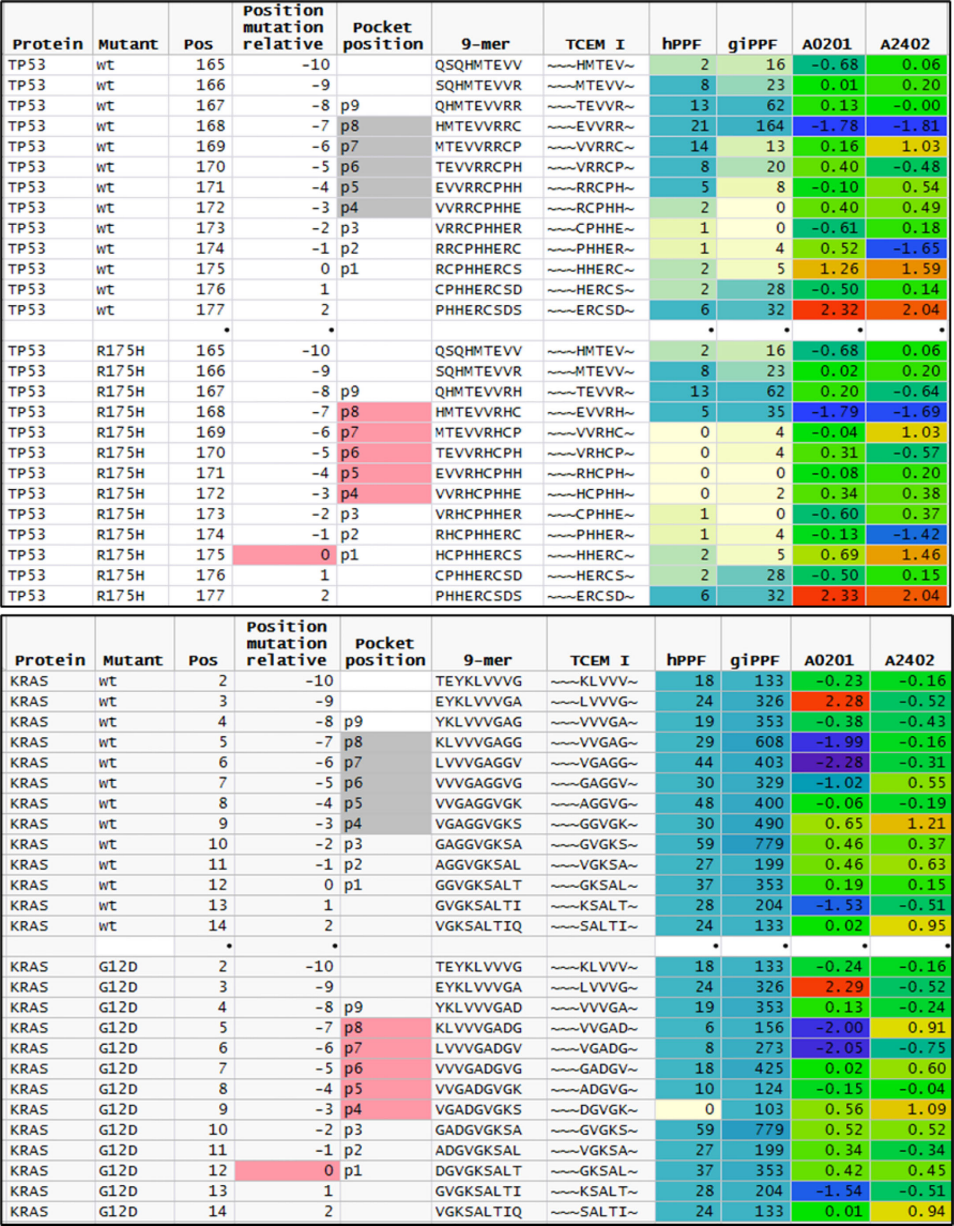
Comparison of featured of sequential peptide position in TP53 R175H and KRAS G12D. Upper plot shows sequential 9mer peptides in TP53 wildtype and R175H tracking the change in GEM vs TCEM position, TCEM amino acids, hPPF, and giPPF and predicted binding for A*02:01 and A*24:01. The baseline hPPF in the wildtype is low, and in the mutant comprises multiple missing (hPPF=0). Lower plot shows the same fields for KRAS G12D, where the baseline hPPF is high. Column headings: Position: index amino acid position in protein; Position mutant relative: index amino acid relative to mutant position; pocket position indicated p1-p9 with TCEM shaded; A*02:01 and A*24:01 is the Z scale predicted binding affinity at every position for these alleles shown in standard deviation units (σ) where blue shading indicates higher affinity.

We examined the TP53 mutations to evaluate further whether a higher count of rare motifs was created by the more commonly recognized deleterious mutations. Of the 471 unique mutations, all had a reduced hPPF count for the mutated TCEM, while 222 had 1 to 7 of the possible 10 TCEM I or TCEM II missing from the human proteome. As shown in **Figure 5**, for the most common TP53 mutation, R175H, 4 of the 5 TCEM I that include the mutant histidine are absent in the human proteome. One TCEM I is also absent in the GI microbiome, while 3 more positions have very low giPPF counts. This implies that of 5 possible CD8+ TCR binding motifs, 4 would be unlikely to encounter a cognate T cell clone and immune surveillance would be dependent on T cells responsive to near-neighbor TCEM. Only the TCEM I ~~~EVVRH~ is represented in the human proteome and in the GI microbiome. Notably the hPPF of the wildtype TCEM is very low across these TP53 pentamers, indicating that only a small reduction in frequency is needed to create multiple missing motifs. We did not determine the HLA of the 3,147 patients with TP53 mutations, and thus did not predict the binding position of the TP53 mutant for each case, but it is assumed that a wide diversity of allele combinations would be present. **Figure 5** shows the peptide binding register that positions the mutated amino acid in p8 of the MHC I binding pocket has a predicted high affinity binding of both A*02:01 and A*24:02 (Z scale binding approximately -1.7σ) as compared to all other registers that would place the mutant in a TCEM. In this scenario A*02:01 and A*24:02 would be likely to bind and present the single TCEM that is represented in both the proteome and GI microbiome albeit as a slightly lower frequency motif compared to the wildtype pentamer. Interestingly, this peptide has been reported by others as an immunogenic neoantigen in TP53 R175H for A*02:01 (4, 85, 86).

KRAS presents a contrasting situation to TP53. The driver dataset included 61 unique KRAS mutations in 1,263 cases. Of the KRAS mutants, 78% are in positions G12 or G13. Within these two positions, only one mutation (G12D) creates a single TCEM I that is absent from the human proteome (~~~DGVGK~). Interestingly, the adjacent TCEM, for G12D and for the other mutations at this position, have a different pattern from TP53: a high hPPF and high giPPF in the wildtype which is somewhat reduced in the mutant. There is a difference in predicted binding of peptides exposing G12D in the example alleles shown, with A*02:01 being likely to present this TCEM and A*24:02 less likely to do so.

### 3.3 Combined T cell exposed motif frequency and HLA binding in GBM and LUSC cases

GBM and LUSC mutant proteins exhibit patterns of lower frequency TCEM comprising mutated amino acids relative to wildtype, like those observed in the driver mutation dataset described above. **Supplemental Figure 4** shows the comparative Poisson hPPF distribution of TCEM I and TCEM II comprising mutant amino acids in the proteins of the GBM and LUSC cases. As shown above for the driver mutations, the Poisson mean hPPF of TCEM I for both GBM and LUSC relative to the wildtype decrease about 50%, from a count of 18.6 (wildtype) to 9.0 (mutant) for TCEM I and 21.0 (wildtype) to 11.2 (mutant) and with about 10% of the TCEM I missing in each case. Likewise for MHC II, the TCEM II hPPF decreases from a count of 17.4 (wildtype) to 9.2 (mutant) for GBM and 17.3 (wildtype) to 10.6 (mutant) for LUSC, with 9-11% of the TCEM motifs missing from the proteome. There are far more passengers than driver mutations in the GBM and LUSC datasets and the wildtype hPPF and giPPF range widely. The patterns are similar between MHC I and MHC II and between the two different cancers, and like those observed in the large oncogene and suppressor gene product set above. The TCEM giPPF distributions are shown in **Supplemental Figure 5**. There is also a decrease in the Poisson mean TCEM giPPF frequencies, but with a smaller fraction missing. Thus, there is an overall pattern in this large population of passenger mutations in which the mutation creates more low-frequency motifs, and comprises a fraction of motifs missing completely from the human proteome, the GI microbiome or both.

#### 3.3.1 Preferred binding register determines mutant presentation to TCR

**Figure 6:**
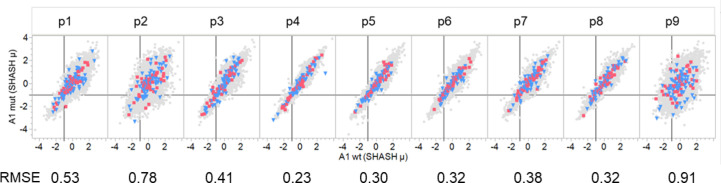
Comparison of binding of peptides with driver and passenger mutations in each pocket position. Comparison of predicted binding to mutant and wildtype peptides by a single MHC A allele that is a composite of the first A alleles of all GBM and LUSC patients. Individual graphs show comparative binding for successive peptides that position the mutant amino acid in pocket positions 1-9. Values are the neural network ensemble means that are transformed to a common scale normal distribution (Z Scale) with SHASH. The symbols in gray show all passenger mutations. Blue triangles are suppressor gene product peptides. Red squares are oncogene gene product peptides. The RMSE value is for a fit of the data in each panel with a regression with a slope of one and an intercept of zero. The vertical black line in each cell is the -1σ Zscale cut point.

We compared the predicted MHC binding of peptides carrying the mutant amino acids from passenger gene products to those of drivers within the GBM and LUSC dataset. Zscale standardized binding predictions comparing the predicted MHC binding of the wildtype peptides to that of the mutant peptides for each 9mer comprising a mutant amino acid is shown in **Figure 6** for the composite of the first (sort order) MHC I A alleles of all GBM and LUSC cases. The dataset for each pocket position was fitted with a regression line with a slope of one and an intercept of zero, that effectively represents the null hypothesis. No difference was noted between the wildtype and mutant peptides (**Figure 6**). The RMSE of patterns around the regression line gives an estimate of the binding variance between the peptide pairs that is induced when the mutant is in the particular pocket position. The known effects of mutation on binding to the pocket positions p1, p2, p3 and p9 are evident by the larger RMSE values, whereas the variation when the mutant amino acid is in TCEM I positions p4, p5, p6, p7,or p 8 is substantially smaller. It is evident in **Figure 6** that the patterns are similar for drivers and passengers and for the two different types of cancer across all patients.

**Figure 7:**
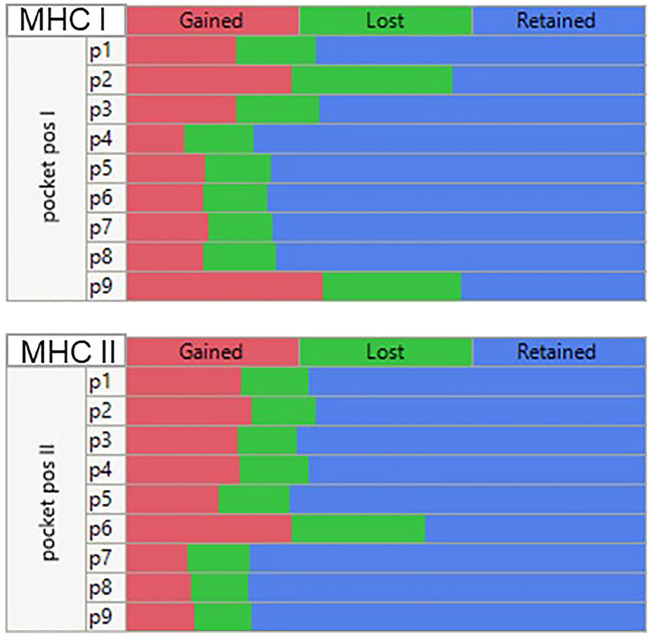
Multiple comparison analysis of predicted peptide binding changes by MHC pocket positions for all mutations in all GBM and LUSC cases analyzed. Multiple categorical comparison which places the peptide amino acid in the indicated pocket position. For this visualization the threshold for the changes was set at a -1σ standard deviation level (approx. 16% percentile). If the binding affinity was <= -1σ before mutation and > -1σ after mutation (reduced affinity) = ‘Lost’. An affinity > -1σ before mutation and <= -1σ after mutation = “Gained” and if there was no change = “Retained”.

Mutations at each of the positions in the peptide may affect the MHC I and MHC II binding and, if the changes are sufficiently large, may alter the presentation register exposed to the potential cognate T cells. **Figure 7** is a composite multiple response analysis of all alleles and all pocket positions for all the GBM and LUSC mutations analyzed. Mutations in the GEM (anchor) positions can either reduce or increase binding. If the binding affinity is increased by the mutation, the mutant amino acid will be more likely to be hidden from the TCR, effectively re-enforcing the presentation of the wild-type TCEM. However, if the mutation decreases the binding affinity, a new binding hierarchy of peptides can arise in that region of the protein, causing a different TCEM register to be exposed to the TCR. The effect of the mutation on the binding register of the peptide containing the mutation is shown in **Figure 7**. This depicts the pattern generated using the criteria of whether the Zscale (-1σ) activity threshold is lost or gained as a result of the mutation, summarized across the entire composite genotype of the GBM and LUSC cases. The patterns indicate that approximately the same number can be lost, gained, or retained, with the predominant effects being due to changes in p9, followed by p2, p1, and p3 of MHC I and in p6 of the MHC II, which based on the numbering system used is the same as p9 of MHC I (**Figure 1**). There is no difference between the pattern for GBM and for LUSC (not shown).

#### 3.3.2 HLA genotype binding as a determinant of potential neoepitopes

**Figure 8:**
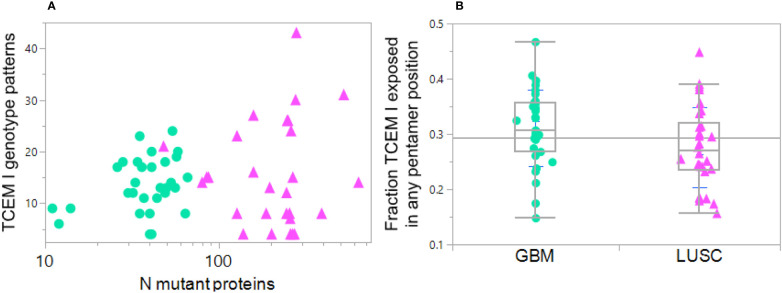
Diversity in HLA genotype binding patterns of mutant peptides in GBM and LUSC cases. Each dot indicates one case GBM: green circles, LUSC pink triangles. **(A)** Variation in the number of MHC I TCEM presentation patterns between different cases compared to the number of total mutant proteins in the particular case. X axis shows the number of mutations per case. The Y axis TCEM I genotype pattern shows the number of the possible 64 combinations in each case that simultaneously fulfill two criteria: 1) expose the mutant amino acid in any of the TCEM I positions and 2) have a predicted MHC binding above the threshold for one or more HLA I allele in the patient’s genotype. The maximum number of combinations would be 64 = 2^6^ in the situation where some of the mutant peptides are bound to all of the different alleles (see Section 2.5). Thus, one of the LUSC case with the highest mutational diversity has approximately 300 mutated proteins and these mutant peptides are distributed across over 40 TCEM exposure-MHC binding combinations, including some peptides with exposed mutations binding to four different alleles. At the lower end of the Y axis are cases where exposure and binding are restricted to a very small number of TCEM-MHC binding combinations, even though some of the cases have a large number of mutated proteins. **(B)** Shows for each case the fraction of all the mutated peptides in which the mutation is exposed in any of the 5 TCEM I motif registers exposing the amino acid side chains and binding occurs to one or more alleles in the patient HLA genotype at Z< -1σ. Each point is the average over all mutations in all proteins in the case. Note that the mean is at approximately 0.3; if binding occurred equally in all of the 9 positions, a mutant amino acid would be placed in a TCEM 5 out of 9 times and the mean would be 0.55. This underscores the dominance of binding placing the mutant in positions 2 or 9 and thereby evading T cell detection.

Many studies restrict analysis to a single MHC allele such as A*02:01 and do not consider the full genotype. Such simplification can seriously restrict the view of how a patient’s genotype may be displaying each peptide across all their MHC alleles. The bitmap pattern was devised to assign two of the most relevant features of a tumor peptide for pMHC exposure to cognate T cells: exposure of a mutant amino acid in the TCEM and its binding affinity. This categorical coding strategy makes it possible to consolidate and compare multiple cancer cases and illuminates the complexity of multi-allele datasets with many peptides. **Figure 8A** shows that among the cases of GBM and LUSC there is a large diversity in binding and mutant amino acid presentation patterns among the cases; a total of 54 of the possible 64 (2^6^ MHC I) different bit patterns are found among the 61 cases by this analysis.

Using the Zscale -1σ threshold, a summary of the data over all 61 cases shows there is considerable variation between patients, ranging from 12% to 35% of the peptides bound to any combination of an individual patient’s alleles while also exposing the mutant amino acid (**Figure 8B**). For the GBM cases the average is 0.33; about 1/3 of the binding registers in all of the patient alleles that bind the peptide at the Zscale cut-off affinity or higher place the mutant amino acid in a binding register exposed to the T cell. The average for LUSC is slightly lower. In both types of cancer the pattern varies greatly between patients and is biased towards being hidden. Overall the level of TCEM presentation is strongly influenced by the diversity of binding patterns of the patient HLA genotype. In patients where the mutant peptides are bound to more HLAs in their genotype, a higher fraction of mutant amino acids are exposed to the T cells.

#### 3.3.3 Combination of mutant peptide binding and TCEM exposure

Combining pMHC binding and TCEM exposure patterns for both class I and also class II alleles generates a more complete picture of the peptide binding and presentation on cells. **Table 1** summarizes the down-selection, starting from the entire sets of mutant peptides for GBM and LUSC, to the subset which are both bound and for which the mutant amino acid is in the TCEM presented to the TCR. The combined potential CD8+ and CD4+ immunogenic neoepitopes are on the bottom line. A complete contingency table providing the underlying data is in **Supplemental Table 5**. **Table 1** shows the 74-78% of the total peptides do not bind to any combination of class I or class II alleles at a higher affinity than the selected Z scale cutoff. Approximately 22-26% of the total bind to MHC I and about 4% of the total meet both the MHC I and MHC II criteria. Nevertheless, it is possible to identify a set of peptides that satisfy the bound/mutant exposed presentation criteria for both MHC I and MHC II in each case.

**Table 1:** Summary statistics of peptides analyzed in 31 GBM and 30 LUSC cases.

	GBM		LUSC	
Protein missense mutations	1,234		6,860	
Total missense peptides 9mers + 15mers comprising mutant amino acid	14,807		85,883	
Total 9mers comprising mutant amino acid	11,106		64,425	
Non-binding mutated 9mers	5,971	54%	38,175	59%
Non-binding + non TCEM I presenting	8,246	74%	50,006	78%
Binding 9mers Z < -1σ for 1 or more alleles in genotype	5,135	46%	26,250	41%
Binding 9mers Z < -1σ + TCEM I presenting	2,860	26%	14,419	22%
Binding 9mers presenting both TCEM I and II Z<-1σ, TCEM _I and Z<-1σ, TCEM_II	484	4%	2,442	4%

#### 3.3.4 Frequency overlaid on HLA genotype

By combining the predicted Zscale binding value for all 9-mer peptides that contain the mutant amino acid over the patient’s entire MHC I genotype matrix of Zscale values (6 (alleles) x5 (registers of potential exposure)), the most probable (dominant) pMHC combination(s) that expose the mutant amino acid can be determined.

To get an overall picture of the effects of combining the three variables: binding, TCEM exposure, and PPF frequency, we combined the binding results of the LUSC and GBM datasets with the hPPF over the range of rare motif frequencies from 0 to 10, the latter being the average mutant TCEM Poisson frequency in the driver and suppressor dataset (see **Figure 3**). The cumulative fractions of most probable pMHC-exposed mutant combinations were then computed from the 6x5 matrix described above. This showed clear differences between the two types of cancer, as well as differences between the drivers vs passengers, not appreciable in the prior analyses.

**Figure 9:**
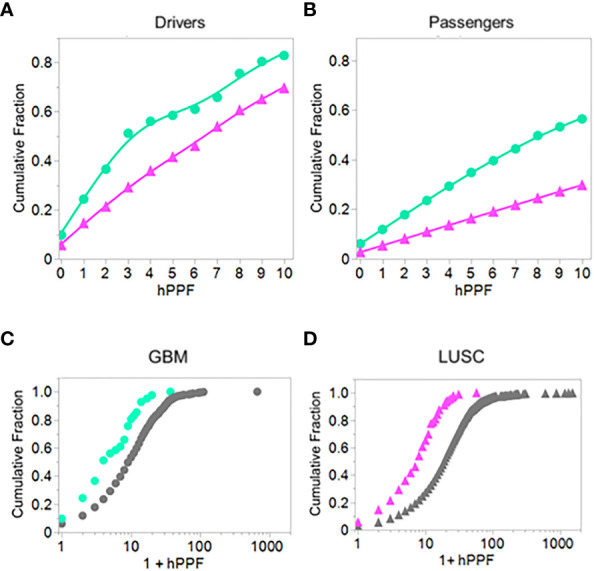
Comparing driver and passenger gene products: Cumulative fraction HLA genotype binding of peptides with TCEM exposed mutants by hPPF count in the human proteome. Cumulative binding computed as in **Figure 2** except for a defined set of peptides presenting the mutant amino acid in the TCEM. **(A)** GBM and LUSC driver mutations. **(B)** GBM and LUSC passenger mutations. **(C)** GBM driver and passenger mutations. **(D)** LUSC driver and passenger mutations. GBM = green circles; LUSC = pink triangles. Data is for all cases of GBM or LUSC combined and is for hPPF selected using -1σ Zscale affinity cut off. The data is best fit with a Weibull growth model: a(1-Exp(-(x/c)^b^)) where a=upper asymptote, b=growth rate,c=inflection point.

**Figure 9** shows the cumulative fraction of all mutants which are bound (Y axis) against the hPPF (X axis) for drivers and passengers. Panels A and B show the results for the lowest frequency pentamers, those having hPPF in the range of 0-10 counts in the proteome. In GBM the TCEM hPPF scores for peptides bound and presented are more rare for both drivers and passengers than in LUSC and this difference is maintained at all levels of hPPF. Moreover, there is a difference between the drivers as compared to the passengers. **Figures 9C, D** show the cumulative fraction for each cancer type, comparing the hPPF of drivers and passengers over the full range of human proteome motif frequencies found in the tumor mutant data sets. As the hPPF motif frequency count increases an inflection is reached that is comparable to the Poisson mean hPPF in each cancer type. The underlying basis for the differential effects between cancers and drivers vs. passengers is not obvious, but in both types of cancers the passengers comprise the vast majority of the mutant peptides.

### 3.4 TCEM frequencies in a group of known neoepitope immunogens

The study reported by Parkhurst et al. (4) is very detailed and provides a comprehensive analysis of mutant identification including tumor, normal, and RNA sequences, mutant curation of allele specific expression, and isolation and characterization of peptide recall responses of reactive T cell clones. This enables us to examine whether the peptides eliciting responding T cells fulfill the criteria of binding and mutant amino acid exposure and to determine the possible fate of TCEM that are pentamers missing from the proteome.

**Figure 10:**
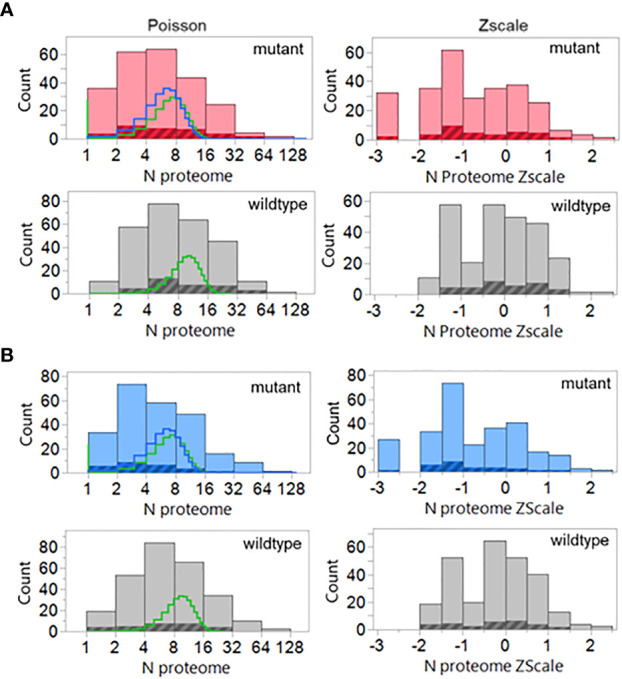
Poisson and Zscale distributions of the peptides previously demonstrated to elicit cognate T cell responses. Counts are for all peptides and all binding registers of potentially T cell exposed amino acids in the 9mers of all cases where authors had identified responding T cells The data assembly process is similar to that used in **Figure 3** including the GEM (i.e. tetramer) frequencies as well. The highlighted areas are the peptides indicated by the authors to be the predicted minimal epitopes. **(A)** MHC I, **(B)** MHC II, compared to wildtype (grey). Lines in the histogram show two different Poisson fits: green=standard fit; blue=-zero inflated model.

The results are shown in **Figure 10**. The shift towards a less common hPPF in the mutant TCEM I as compared to wildtype seen in the datasets analyzed above is also present here. The wildtype hPPF Poisson mean λ is 10.4 compared to the mutant λ of 7.6, and 12% of the TCEM are absent from the human proteome. The cross-hatched area in the histograms in **Figure 10** show the hPPF score of the peptides that the authors identified as the predicted minimal epitope. All but two of the peptide-allele combinations identified by the authors as producing T cell responses are found in the human proteome. For these two peptides we predicted that a different, or additional, MHC allele than that initially indicated may have been preferentially binding and presenting the mutant amino acids in a position that generated a more common TCEM. We find that the 9mer peptides identified in that paper were predicted as potential responders by all three factors in our multivariate selection criteria. The authors did not report MHC II activity, but all of the peptides in the dataset had overlapping longer peptides with relatively high DRB1 MHC II binding affinity.

## 4 Discussion

Mutations detected in tumor biopsy proteins are those which have not been previously eliminated by effective immune surveillance. We show that tumor mutations identified in biopsies have TCEM pentamers that have lower counts in the human proteome, lower counts in the gastrointestinal microbiome, or in both, than their unmutated wildtype counterparts. A mutant TCEM in a driver protein has, on average, six fewer copies of matching pentamers in the human proteome than does the corresponding TCEM in the wildtype protein (**Figure 3**). Approximately 10% of the TCEMs comprising the mutant amino acid in an oncogene or tumor suppressor gene product have no matching pentamer in the human proteome. A lower hPPF reduces the probability that a cognate T cell clone was selected in the thymus. Mutants also generate pentamer motifs less commonly found in the larger GI microbiome reference dataset, also indicative of a lower probability of a cognate T cell in the repertoire.

We confirm the observations of others that mutant amino acids are more likely to be found in peptide positions 2 and 9 of a MHC I binding groove than in the TCEM, and so are more likely to be hidden from the TCR (2, 14, 87, 88). Mutations can change the peptide binding hierarchy within the context of the patient HLA genotype. We show that MHC II mutant amino acids are found preferentially in position 6 of the central 9mer of a binding 15mer peptide, also an anchor position concealed in the MHC groove. When a mutant amino acid is in the anchor positions, the amino acid motifs exposed to the TCR are not differentiated from wildtype.

In both GBM and LUSC datasets more mutant amino acids are hidden in GEM positions than exposed to T cells. In peptides predicted to have affinity to one or more of the patient HLA molecules, the mutant amino acid was in the exposed p4, p5, p6, p7, p8 positions on average in only 25-30% of them. If the distribution were random, one would expect 5 of 9 amino acids (i.e. 56%) to be exposed. So there is approximately a two-fold selection against mutant exposure and in many of cases of both cancer types the evasion bias was even greater.

Applying the -1σ Zscale as a threshold, 54% of the GBM and 59% of the LUSC peptides containing mutants did not bind to any of the 6 MHC I alleles of the patients. When the predicted TCEM frequency is combined with HLA binding in the GBM and LUSC cases, only 22-25% of the peptides comprise mutant amino acids positioned where they are presented to the TCR by one or more HLA alleles, and also have a TCEM that is a pentamer motif found in the human proteome (hPPF >0). The combination of non-exposure and rare motifs significantly reduces the chance that the tumor amino acid mutation will be an immunogenic neoepitope through TCR engagement and T cell activation. While a TCEM pentamer with no match in the human proteome is the extreme case, the mean frequency count was reduced across all tumor mutants in these patients.

Similar combinatorial binding and frequency relationships were observed for MHC I bound peptides with potential to stimulate a CD8+ cytotoxic T cell response and for MHC II bound peptides with potential to stimulate a CD4+ T cell response. The probability of MHC I *and* MHC II both fulfilling the criteria for binding and mutant exposure was determined for each GBM and LUSC patient and found to be approximately 4% of the total mutant peptides. Although GBM and LUSC differ in the number of mutations each cancer type typically comprises, and in many other features, the patterns of TCEM hPPF were very similar, with the Poisson mean of the hPPF reduced in mutants and a fraction of the mutant TCEM pentamers missing altogether from the proteome. Similar patterns of binding and TCEM hPPF were observed in both mutated driver and passenger gene products.

When a group of mutated tumor 9mer peptides previously demonstrated (4) to elicit T cell responses in the patients were examined, they were found to fulfill the criteria of binding for one or more of the patient’s HLA alleles and the corresponding TCEM having an adequate hPPF. Notably, 51 of 53 responders presented TCEM with hPPF >0; only 2 were are absent from the human proteome but were found to be present in the GI microbiome.

### 4.1 Limitations

The present study has several limitations. First, we evaluated the TCEM within each mutated protein without consideration of the level of expression of that protein in the tumor or whether the mutant was found in the RNA. Expression of the mutated gene, and particularly of the mutant haplotype, is one of the major limitations of neoepitope antigenicity. Only a small percentage of the alleles with in-frame mutations in coding sequences detected in tumor DNA are actually transcribed (4, 89, 90). Levels of expression may differ widely between proteins and cell types even in normal tissue, and may be further distorted in tumors by changes in gene copy number. Parkhurst et al. (4) show that as few as 30% of mutated amino acids could be detected in in the transcriptome, even in highly expressed mRNA. Our own observation of clinical cases is that in some cases as few as 15% of mutated amino acids can be found in transcripts (unpublished observations). While overall tumor burden (percent of cells in a biopsy carrying the mutation) can affect targeting and detection, we did not address it here as a factor in determining immunogenicity.

In considering the probable frequency of presentation of a given motif from the human proteome during positive thymic selection we were not able to consider differential transcription in the thymic epithelium nor biases which may be created by thymoproteasomal or enzymatic cleavage in the thymus (91–95). We also did not evaluate potential changes in cathepsin cleavage in mutated proteins as a factor in presentation of tumor-specific peptides by antigen presenting cells in the tumor microenvironment. A number of commonly observed SNP mutations generate amino acid changes that would affect cleavage by endosomal cathepsin B, L or S.

We limited the tumor mutations analyzed to single amino acid missense variants. We excluded mutations which resulted in a loss of a stop signal, indels, splice variants, and fusions. There is no reason to think that other types of mutation, if expressed and generating tumor-specific TCEM, have a different pattern of immune escape, and this is consistent with our unpublished observations.

We analyzed relatively small case numbers of two cancers with different mutational burdens. In these, consideration of MHC II was limited to DRB alleles. This allowed us to develop estimates of variability and gain insight into how to undertake a systematic larger-scale analysis to include a wider variety of cancer types. Different cancer types will exhibit variations on the patterns we observed. Furthermore, we did not address non-classical MHC-T cell interactions (96, 97).

We include the GI microbiome ecosystem in our consideration because it is a recognized contributor to T cell repertoire diversity and it has been shown to predict the T cell response to checkpoint inhibitors (7, 37, 98). The representative GI microbiome dataset we used is necessarily a generalization of a dynamic and diverse population of microbes that differ between individuals and over time. While derived from open reading frames, the dataset does not address levels of expression. Other exogenous epitope exposures, including pathogenic, environmental and interventional epitopes, such as biopharmaceuticals or vaccines, also contribute to the repertoire of peptides and hence exposures that shape the T cell repertoire over a lifetime, although likely to a less continuous and less diverse degree than the microbiome.

Immunogenicity is a two-sided relationship. We have examined here the potential presentation and exposure of tumor-specific neoepitopes. We have not addressed the other side of the relationship: the avidity of the TCR for the pMHC, nor the cognate T cell clonal population sizes within a patient’s T cell repertoire.

### 4.2 Impact of TCEM pentamer frequency on tumor immunogenicity

An amino acid pentamer is limited to 3.2 million possible different combinations. The role of this limitation in polyspecificity of T cell responses has been recognized (28). Polyspecificity of T cells, in which a TCR may engage the same exposed motif in multiple antigens, is essential to accommodate responses to all the possible novel antigens which an individual may encounter (99–101). Any reduction in T cell repertoire diversity increases the potential for gaps permitting some epitopes to escape detection. The earliest T cell repertoire is the product of thymic selection (34). A focus on negative selection in central tolerance overlooks the impact that absence of a pentamer from the human proteome may have on the diversity of positive selection and the foundational T cell repertoire by precluding its presentation in the thymus (33, 90, 101). An individual’s repertoire is maintained, in active circulation and memory, by a lifetime of exposure to endogenous and exogenous stimuli. The age at which such exposures occur determines whether this builds a more diverse T cell repertoire or depletes it. Ageing is accompanied by a progressive loss of T cell repertoire diversity (42, 43, 45, 102). In cancer, a patient with a less diverse or declining repertoire eventually arrives at a pivot point at which immune evasion occurs and tumor progression ensues (42, 43). The appearance through mutation of less common TCEM will exacerbate the chance of evasion.

### 4.3 Homozygosity as a risk factor and the role of multiple-HLA allele presentation of peptides

Heterozygosity of HLA has long been recognized as enhancing the breadth of immune surveillance (103). Homozygosity at any HLA locus is a risk factor for tumor immune evasion (104, 105). Such increased risk may come about in multiple ways. HLA homozygosity reduces the options for presentation of a self-peptide during positive selection in the thymus, reducing the breadth of the initial T cell repertoire. The same limitation applies in the lifetime stimulation of T cells and replenishment of memory in response to exogenous antigens. In cancer, HLA homozygosity offers fewer means to present a neoepitope to T cells. While several of the patients in the GBM and LUSC datasets were homozygous for some alleles, the datasets are too small to address the impact of this definitively.

### 4.4 Combined CD4+ and CD8+ T cell responses

An optimal response to a tumor-specific neoantigen requires both CD8+ and CD4+ T cells (15). Using the overlay of binding and mutant amino acid exposure to the TCR embedded in our categorical approach and co-selecting for both class I and class II responses yields only 4% of mutants with TCEM matched by pentamers in the human proteome. For MHC II, only the DR alleles were considered; perhaps the DP and DQ alleles offer an advantage, but as noted, these present analytical challenges. However, even a multiple of the 4% value by each of these loci would still leave a major response limitation. This low number approximates that found experimentally, when consideration is given to the limited allele-specific expression (4). These estimates based on the human proteome are mitigated by the effect on the T cell repertoire of exposure to exogenous epitopes, including the GI microbiome. However, the implication is that for cases with low mutational loads, it may be difficult to obtain significant numbers of targets as immunogenic neoepitopes.

The approach used here to generate predicted peptide binding for the whole proteome for a simulated genotype of common HLA alleles, and to determine the potential cross-presentation makes it possible to derive a view of multi-allele MHC occupancy and thus T cell presentation. The sigmoid curve on a logarithmic scale (**Figure 1**) is like that in a receptor binding assay. Using the Zscale cut point near the inflection point is akin to a Kd value, a parameter often used in receptor binding comparisons. It is possible to use simulated patient genotype-proteome binding data in conjunction with transcript levels or protein expression levels to derive a quantitative view of pMHC occupancy diversity. Although derived here in a different way from the work of Yarmakovich et al. (59), the mid-point of 3300 nM when back calculated from a Z scale to the A*02:01 scale is comparable to the 1000 nM in that report, as well as values reported by Duan et al. (2). It is less stringent than the 500nM often used but likely all lie within a range that would be indistinguishable in bench-level receptor assays. Parkhurst et al. were able to obtain peptide recall responses of T cells with about 1.7% of the total neoepitopes evaluated (4). Considering that the mutant amino acid was found in about 30% of the transcriptomes in that study, our values would be very similar to their findings when allele specific expression is considered. This gives credence to the down-selection approach we describe. It further suggests that emphasis on only the highest affinity peptides, such as are detected by mass spectroscopy, may underestimate the full scope of potential immunogenic neoepitopes, and especially when the source of high affinity may be due to the mutant amino acids hidden in the anchor positions (88, 106).

### 4.5 Epitope dissimilarity from wildtype

Dissimilarity between wildtype and mutant peptides has been considered an indicator of likely immunogenicity (5). Our results suggest a more granular approach is needed, in which the portions of the peptide involved in binding to the MHC and those involved in T cell recognition are dealt with in their functional context. The differential agretopicity index (DAI) was designed to describe the difference in MHC binding between mutant and wildtype, as described by the scoring in NetMHC (https://services.healthtech.dtu.dk/), noting that the widest differential in binding was associated with changes in positions 2 or 9 of a 9mer MHC I bound peptide (2). We show here that the differential predicted binding between wildtype and mutants for any individual HLA is rarely large, and that mutation does not significantly change binding of many peptides, except when the mutant occurs in the binding groove, as observed by Duan et al. (2). Quantifying changes in DAI as an index of dissimilarity presumes that the mutated amino acid is in a GEM anchor position. But this in turn means the mutant amino acid is not exposed to the TCR in a TCEM and thus is unlikely to engage a different set of T cell clones than the wildtype.

Others have taken a more complex approach. Luksza et al. (107–110) use the concept of epitope “fitness” based on binding affinity and the similarity of a neoepitope to immunogenic antigens in public repositories. Again, this depends on MHC binding, but introduces the concept of cross reactivity and similarity (or lack thereof) to non-self peptide epitopes curated by IEDB as having generated a T cell peptide recall response. Our approach uses a discrete numerical frequency of TCEM-matched pentamer frequencies within the reference human proteome and GI microbiome. The hPPF and giPPF scores relative to these proteomes is a different metric for fitness more directly related to TCR engagement.

TCEM motifs that do not match pentamers found in the proteome are the archetype of ‘non-self’. However, as noted in the context of microorganisms (28, 35), the simple concept of self and not-self is confounded by the polyspecificity of T cell recognition. Two peptides may share identical TCEM, but have completely different, or very similar, binding affinities and bind to different HLA alleles.

### 4.6 Unique individual combinations

The role of each mutant amino acid in a tumor protein must be considered at each possible position within a potential peptide that may bind to each HLA allele the patient carries. The role of the mutant amino acid in the binding groove is as a determinant of MHC binding affinity, but when exposed it is critical to TCR engagement (111). This must then be considered for each MHC allele, locus and class. Many investigators only consider one allele, most often A*02:01. Others have opted to generalize and use a mean value for agretopicity across multiple neopitopes (112). However, a complete understanding of the functional changes that each mutation generates requires consideration of the entire HLA genotype of the individual. The combinatorial selection based on statistical standardization principles and multiple relevant properties is specific to a patient’s HLA genotype and the frequency spectrum of peptide motifs presented by their tumor. Among just the 61 patients in the GBM and LUSC sets, we found 54 of the possible 64 patterns (2^6^ HLA alleles) of possible binding and TCEM exposure with several patients having proteins with mutated peptides that bound to as many as 20 different peptide-HLA combinations, illustrating the uniquely personal differences.

### 4.7 Mutated tumor proteins differ in potential modes of evasion

Not all tumor mutations produce motifs missing from the human proteome, but virtually all produce lower frequency motifs. In KRAS, G12D generates a single TCEM that is missing from the human proteome; but also 4 TCEM I that have a reduced frequency. The other mutations which are recorded at this position in KRAS (G12V, G12C, etc.) also have a reduced hPPF relative to wildtype but not missing from the human proteome. Surrounding the mutant motifs at positions 12 and 13 in KRAS are many common motifs, among the highest counts in the entire proteome and GI microbiome. This may contribute to the reported downregulated tumor microenvironment for KRAS (113) and the detection of responding T cells effective in adoptive therapy (114). The shift of the passenger cumulative binding curves for the passenger mutations towards more common motifs (**Figures 9C, D**) could be interpreted as another manifestation of the same underlying behavior. Conversely, the most common TP53 mutation R175H occurs in a region with very few common TCEM reducing the chance of generating a CD8+ T cell response. Not all HLA alleles provide binding that would expose the one remaining R175H TCEM with higher human proteome and microbiome representation, although A*02:01 and A*24:02 are predicted to bind appropriately (**Figure 5**). Detection *in vitro* of T cell responses to a single TCEM is challenged by the sampling probability of capturing representatives of a set of likely small clones. Nevertheless, such narrow windows of opportunity can generate effective T cell responses (4, 85, 86). Not surprisingly, given the overall uncommon TCEM arising from this mutation, others were unable to detect T cell responses to R175H with cells from healthy donors (108).

### 4.8 Cross-reactive T cells

Each peptide-MHC pair may stimulate many different cognate T cell clones, each with different αβ TCR and different degrees of binding affinity (46). Not only are T cells necessarily polyspecific, but T cell clones arising initially from stimulation by near-neighbor epitopes, may engage with a particular TCEM (47). Thus, the T cell recall response to any peptide is a quorum response comprising a combination of unique TCR. In some cases this may occur with other less closely associated TCEM (49). When a TCEM is missing from the human proteome and microbiome, the T cell response would be more dependent on recognition by T cells responsive to such near-neighbor epitopes. This was not found to compensate for rare TCEM in infectious agents (35).

Many of the TCEM-matched pentamers that are absent or low frequency in the human proteome comprise cysteine, tryptophan, methionine, or histidine (28). While this is a product of codon numbers, it is also likely to reduce the chance of near-neighbors generating cross reactive T cells. This is the case especially for histidine and tryptophan, given the chemical dissimilarities of their amino acid side chains. More featureless TCEM are likely to have greater T cell cross reactivity (115).

### 4.9 Role of the microbiome

Our work shows the criticality T cell repertoire diversity in tumor immune surveillance. We assessed the TCEM pentamers relative to the GI microbiome because this is a source of a diverse array of pentamers that overlaps, but differs from, those in the human proteome (28). There is increasing awareness of the role of the microbiome in T cell repertoire development (37) and the benefits of a diverse microbiome early in life (116). The impact on tumor immunity of the microbiomes of mice raised in different environments has been clearly demonstrated (6). Notably tumor control following administration of check-point inhibitor drugs is related to differences in the patient’s GI microbiome (7, 98), and prior antibiotic administration (117). A microbiome that maintains a more diverse T cell effector repertoire would be consistent with better targeting of the uncommon TCEM created by tumor mutations, when unleashed by checkpoint inhibition. Greater microbial cross-reactivity therefore correlates with better neoantigen “fitness” (107).

### 4.10 Long time course and passengers

Analysis of mutations in tumors is inherently a study of survivorship bias. Tumor mutations detected in biopsies reflect a long development process, often years, from the initial occurrence of nucleotide mutations to clinical presentation. Tumor progression presupposes a replicative advantage accrues to the cells bearing mutations (118). The findings of this study support the key role of immune selective pressure in determining the subset of expressed mutations and their neoepitopes that remain at the time of biopsy (58). While a similar pattern of MHC binding combined with TCEM frequency was observed in GBM and LUSC cases, it is possible that the pattern is different in tumors of other tissues, rates of development, and initiation. For instance, patterns may differ in pediatric cancers with low mutational burdens, tolerization, and an immature T cell repertoire. The finding of a similar pattern of TCEM hPPF in passenger mutations as in the driver oncogenes and suppressors was unexpected. Passenger mutations are generally thought to accumulate progressively after an initial driver mutation (69). That they exhibit similar patterns of TCEM frequency, exposure and binding suggests that they may have been subject to the same selective pressures and, in some instances, may also be long-standing. Whether this holds true in metastases or in tumors of viral origin remains to be determined. The occurrence of rare TCEM motifs occur in both driver and passenger gene products suggests that they differ from amino acid changes that might be expected from evolutionary changes over millennia. For example, KRAS mutation G12C adds an unpaired cysteine into the KRAS protein. That free cysteine may form inappropriate disulfide bonding with other similarly mutated KRAS molecules forming dimers of unknown properties or form bimolecular complexes with other proteins. Similarly, random insertion of other amino acids with inappropriate physicochemical properties into other proteins could be detrimental to protein structure, activity, or stability. Others have noted that some MHC prediction algorithms sometimes give aberrant results with neoepitopes (59, 106). Given the insertion of highly rare combinations of amino acids by tumor mutations, the use of algorithms that depend on substitution matrices, which are derived from evolutionary amino acid changes, may be less suitable for prediction of unusual amino acid substitutions occurring in cancer.

### 4.11 Tumor specificity as a microcosm

An effective T cell response to a tumor is remarkably different from the response to an infectious agent in the precision that it requires. In a tumor T cell recognition and distinction of a single missense mutation from the wildtype homologue rests on only five TCEM I and five TCEM II pentamer registers comprising the mutated amino acid. A virus or bacteria confronts the T cell repertoire with a large array of potential targets overlaid along the full length of each of its proteins, giving multiple opportunities for MHC I and MHC II cross-presentation to effect a successful immune response. As we show, specific cross-presentation is a rare coincidence in tumor mutations. Targeting the tumor neoepitope is thus far more exacting, demanding alignment of MHC binding to expose the mutant and the presence of cognate T cells responsive to specific TCEM. Hence, there is a far greater probability of failure in targeting a tumor than an infectious agent. Conversely, the very narrow focus, indeed the relative simplicity, of defining a tumor-specific response also enables us to better understand the criteria for immunogenicity that play out in concert across every position in a microbial protein.

### 4.12 Immune pressure and genomic changes

Underlying the tumor-specific amino acid mutations in a tumor biopsy is an array of nucleotide mutations. Distinctive genome signatures in tumor biopsies are potentially indicators of initial causality and changes in gene function (119–122). Most nucleotide mutations in a tumor do not lie within protein coding regions. Mutations may, or may not, confer replicative advantage, whether they occur in in introns, 5’ and 3’ gene control regions, intergenic regions, or in exons. However, only mutations in protein coding regions will affect the immune pressure. The rare motifs in tumor mutated proteins emerge in the TCEM that engage TCR, not when the mutants are hidden from TCRs in the GEMs. This supports the role of immune pressure as a dominant factor in determining the landscape of those protein mutations surviving to be detected in biopsies (58).

The most common nucleotide changes are C>T, which dominate genome signatures of ageing (119, 120). The common arginine to histidine missense mutations are the product of a G>A nucleotide change, equivalent to a counterstrand C>T. Histidine features frequently in rare TCEM. Similarly, the common proline to leucine is produced by a C>T mutation and will almost certainly modify the processing by cathepsins in macrophages, dendritic or B cells and thus drastically change pMHC presentation to CD4+T cells (123–125).

## 5 Conclusions

Immune evasion handicaps an effective T cell response directed to a tumor cell expressing a mutated protein. Such evasion, combined with tumor cell replication, eventually tips the balance towards tumor progression. Our results shed light on some of the factors contributing to immune evasion and the low number of tumor mutations that result in an immunogenic neoepitope. We identify the previously unreported role in immune evasion of the frequency characteristics of the pentamers produced by mutation in T cell exposed motifs that engage the TCR. Tumor mutations are characterized by less common T cell exposed pentamer motifs than their wildtype counterpart and have a lower probability of encountering cognate TCR in the T cell repertoire.

The discussion of implications and limitations identified here underscores the complexity of the interface between tumorigenesis and immune surveillance and the challenges of analyzing a highly complex multivariate system. To understate this complexity in the design of immunotherapeutic interventions is to risk unexpected outcomes.

The interaction of HLA genotype and peptide binding, mutant positioning, and the mutation-specific T cell exposed motif frequency emphasize the need to evaluate potential neoepitope targets precisely, individually, and within the unique context of each patient and their complete HLA genotype to optimally utilize the immunogenic epitopes. These are important considerations in the design of neoepitope vaccines.

## Data availability statement

The original contributions presented in the study are included in the article/**Supplementary Material**. Further inquiries can be directed to the corresponding authors.

## Author contributions

All authors listed have made a substantial, direct, and intellectual contribution to the work and approved it for publication.

## References

[1] Tran E Robbins PF Rosenberg SA. 'Final common pathway' of human cancer immunotherapy: targeting random somatic mutations. Nat Immunol (2017) 18(3):255–62. doi: 10.1038/ni.3682 PMC6295671 28198830

[2] Duan F Duitama J Al Seesi S Ayres CM Corcelli SA Pawashe AP. Genomic and bioinformatic profiling of mutational neoepitopes reveals new rules to predict anticancer immunogenicity. J Exp Med (2014) 211(11):2231–48. doi: 10.1084/jem.20141308 PMC4203949 25245761

[3] Schumacher TN Schreiber RD. Neoantigens in cancer immunotherapy. Science (2015) 348(6230):69–74. doi: 10.1126/science.aaa4971 25838375

[4] Parkhurst MR Robbins PF Tran E Prickett TD Gartner JJ Jia L. Unique neoantigens arise from somatic mutations in patients with gastrointestinal cancers. Cancer Discovery (2019) 9(8):1022–35. doi: 10.1158/2159-8290.CD-18-1494 PMC7138461 31164343

[5] Wells DK van Buuren MM Dang KK Hubbard-Lucey VM Sheehan KCF Campbell KM. Key parameters of tumor epitope immunogenicity revealed through a consortium approach improve neoantigen prediction. Cell (2020) 183(3):818–34 e13. doi: 10.1016/j.cell.2020.09.015 33038342 PMC7652061

[6] Bessell CA Isser A Havel JJ Lee S Bell DR Hickey JW. Commensal bacteria stimulate antitumor responses *via* T cell cross-reactivity. JCI Insight (2020) 5(8):e135597. doi: 10.1172/jci.insight.135597 32324171 PMC7205429

[7] Gopalakrishnan V Spencer CN Nezi L Reuben A Andrews MC Karpinets TV. Gut microbiome modulates response to anti-PD-1 immunotherapy in melanoma patients. Science (2018) 359(6371):97–103. doi: 10.1126/science.aan4236 29097493 PMC5827966

[8] Garcia KC Teyton L Wilson IA. Structural basis of T cell recognition. Annu Rev Immunol (1999) 17:369–97. doi: 10.1146/annurev.immunol.17.1.369 10358763

[9] Rudolph MG Stanfield RL Wilson IA. How TCRs bind MHCs, peptides, and coreceptors. Annu Rev Immunol (2006) 24:419–66. doi: 10.1146/annurev.immunol.23.021704.115658 16551255

[10] Calis JJ de Boer RJ Kesmir C. Degenerate T-cell recognition of peptides on MHC molecules creates large holes in the T-cell repertoire. PloS Comput Biol (2012) 8(3):e1002412. doi: 10.1371/journal.pcbi.1002412 22396638 PMC3291541

[11] Naumov YN Hogan KT Naumova EN Pagel JT Gorski J. A class I MHC-restricted recall response to a viral peptide is highly polyclonal despite stringent CDR3 selection: implications for establishing memory T cell repertoires in "real-world" conditions. J Immunol (1998) 160(6):2842–52. doi: 10.4049/jimmunol.160.6.2842 9510187

[12] Falk K Rotzschke O Stevanovic S Jung G Rammensee HG. Allele-specific motifs revealed by sequencing of self-peptides eluted from MHC molecules. Nature (1991) 351(6324):290–6. doi: 10.1038/351290a0 1709722

[13] Fritsch EF Rajasagi M Ott PA Brusic V Hacohen N Wu CJ. HLA-binding properties of tumor neoepitopes in humans. Cancer Immunol Res (2014) 2(6):522–9. doi: 10.1158/2326-6066.CIR-13-0227 PMC4049249 24894089

[14] Calis JJ Maybeno M Greenbaum JA Weiskopf D De Silva AD Sette A. Properties of MHC class I presented peptides that enhance immunogenicity. PloS Comput Biol (2013) 9(10):e1003266. doi: 10.1371/journal.pcbi.1003266 24204222 PMC3808449

[15] Alspach E Lussier DM Miceli AP Kizhvatov I DuPage M Luoma AM. MHC-II neoantigens shape tumour immunity and response to immunotherapy. Nature (2019) 574(7780):696–701. doi: 10.1038/s41586-019-1671-8 31645760 PMC6858572

[16] Zander R Schauder D Xin G Nguyen C Wu X Zajac A. CD4(+) T cell help is required for the formation of a cytolytic CD8(+) T cell subset that protects against chronic infection and cancer. Immunity (2019) 51(6):1028–42 e4. doi: 10.1016/j.immuni.2019.10.009 31810883 PMC6929322

[17] Sun JC Bevan MJ. Defective CD8 T cell memory following acute infection without CD4 T cell help. Science (2003) 300(5617):339–42. doi: 10.1126/science.1083317 PMC2778341 12690202

[18] Janssen EM Lemmens EE Wolfe T Christen U von Herrath MG Schoenberger SP. CD4+ T cells are required for secondary expansion and memory in CD8+ T lymphocytes. Nature (2003) 421(6925):852–6. doi: 10.1038/nature01441 12594515

[19] Bremel RD Homan EJ. Recognition of higher order patterns in proteins: immunologic kernels. PloS One (2013) 8(7):e70115. doi: 10.1371/journal.pone.0070115 23922927 PMC3726486

[20] Kreiter S Vormehr M van de Roemer N Diken M Lower M Diekmann J. Mutant MHC class II epitopes drive therapeutic immune responses to cancer. Nature (2015) 520(7549):692–6. doi: 10.1038/nature14426 PMC4838069 25901682

[21] Reddehase MJ Rothbard JB Koszinowski UH. A pentapeptide as minimal antigenic determinant for MHC class I-restricted T lymphocytes. Nature (1989) 337(6208):651–3. doi: 10.1038/337651a0 2465495

[22] Bremel RD Homan EJ. Frequency patterns of T-cell exposed amino acid motifs in immunoglobulin heavy chain peptides presented by MHCs. Front Immunol (2014) 5:541. doi: 10.3389/fimmu.2014.00541 25389426 PMC4211557

[23] Birnbaum ME Mendoza JL Sethi DK Dong S Glanville J Dobbins J. Deconstructing the peptide-MHC specificity of T cell recognition. Cell (2014) 157(5):1073–87. doi: 10.1016/j.cell.2014.03.047 PMC4071348 24855945

[24] Wei P Jordan KR Buhrman JD Lei J Deng H Marrack P. Structures suggest an approach for converting weak self-peptide tumor antigens into superagonists for CD8 T cells in cancer. Proc Natl Acad Sci USA (2021) 118(23):e2100588118. doi: 10.1073/pnas.2100588118 34074778 PMC8201969

[25] Wang Y Sosinowski T Novikov A Crawford F Neau DB Yang J. C-terminal modification of the insulin B:11-23 peptide creates superagonists in mouse and human type 1 diabetes. Proc Natl Acad Sci USA (2018) 115(1):162–7. doi: 10.1073/pnas.1716527115 PMC5776820 29255035

[26] Nelson RW Beisang D Tubo NJ Dileepan T Wiesner DL Nielsen K. T Cell receptor cross-reactivity between similar foreign and self peptides influences naive cell population size and autoimmunity. Immunity (2015) 42(1):95–107. doi: 10.1016/j.immuni.2014.12.022 25601203 PMC4355167

[27] Rossjohn J Gras S Miles JJ Turner SJ Godfrey DI McCluskey J. T Cell antigen receptor recognition of antigen-presenting molecules. Annu Rev Immunol (2015) 33:169–200. doi: 10.1146/annurev-immunol-032414-112334 25493333

[28] Bremel RD Homan J. Extensive T-cell epitope repertoire sharing among human proteome, gastrointestinal microbiome, and pathogenic bacteria: implications for the definition of self. Front Immunol (2015) 6. doi: 10.3389/fimmu.2015.00538 PMC4617169 26557118

[29] Klein L Kyewski B Allen PM Hogquist KA. Positive and negative selection of the T cell repertoire: what thymocytes see (and don't see). Nat Rev Immunol (2014) 14(6):377–91. doi: 10.1038/nri3667 PMC4757912 24830344

[30] Takaba H Takayanagi H. The mechanisms of T cell selection in the thymus. Trends Immunol (2017) 38(11):805–16. doi: 10.1016/j.it.2017.07.010 28830733

[31] Fulton RB Hamilton SE Xing Y Best JA Goldrath AW Hogquist KA. The TCR's sensitivity to self peptide-MHC dictates the ability of naive CD8(+) T cells to respond to foreign antigens. Nat Immunol (2015) 16(1):107–17. doi: 10.1038/ni.3043 PMC4270846 25419629

[32] Michelson DA Hase K Kaisho T Benoist C Mathis D. Thymic epithelial cells co-opt lineage-defining transcription factors to eliminate autoreactive T cells. Cell (2022) 185(14):2542–58 e18. doi: 10.1016/j.cell.2022.05.018 35714609 PMC9469465

[33] Davis MM. Not-So-Negative selection. Immunity (2015) 43(5):833–5. doi: 10.1016/j.immuni.2015.11.002 26588773

[34] Sousa L Rodrigues PM Alves NL. T Cell selection in the thymus: new routes towards the identification of the self-peptide ligandome presented by thymic epithelial cells. Eur J Immunol (2023) 53(3):e2250202. doi: 10.1002/eji.202250202 36642953

[35] Koncz B Balogh GM Papp BT Asztalos L Kemeny L Manczinger M. Self-mediated positive selection of T cells sets an obstacle to the recognition of nonself. Proc Natl Acad Sci U S A (2021) 118(37):e2100542118. doi: 10.1073/pnas.2100542118 34507984 PMC8449404

[36] Hebbandi Nanjundappa R Sokke Umeshappa C Geuking MB. The impact of the gut microbiota on T cell ontogeny in the thymus. Cell Mol Life Sci (2022) 79(4):221. doi: 10.1007/s00018-022-04252-y 35377005 PMC11072498

[37] Zegarra-Ruiz DF Kim DV Norwood K Kim M Wu WH Saldana-Morales FB. Thymic development of gut-microbiota-specific T cells. Nature (2021) 594(7863):413–7. doi: 10.1038/s41586-021-03531-1 PMC8323488 33981034

[38] Ennamorati M Vasudevan C Clerkin K Halvorsen S Verma S Ibrahim S. Intestinal microbes influence development of thymic lymphocytes in early life. Proc Natl Acad Sci U S A (2020) 117(5):2570–8. doi: 10.1073/pnas.1915047117 PMC7007548 31964813

[39] Hadeiba H Lahl K Edalati A Oderup C Habtezion A Pachynski R. Plasmacytoid dendritic cells transport peripheral antigens to the thymus to promote central tolerance. Immunity (2012) 36(3):438–50. doi: 10.1016/j.immuni.2012.01.017 PMC3315699 22444632

[40] Klein L Hinterberger M Wirnsberger G Kyewski B. Antigen presentation in the thymus for positive selection and central tolerance induction. Nat Rev Immunol (2009) 9(12):833–44. doi: 10.1038/nri2669 19935803

[41] Murray JM Kaufmann GR Hodgkin PD Lewin SR Kelleher AD Davenport MP. Naive T cells are maintained by thymic output in early ages but by proliferation without phenotypic change after age twenty. Immunol Cell Biol (2003) 81(6):487–95. doi: 10.1046/j.1440-1711.2003.01191.x 14636246

[42] Palmer DB. The effect of age on thymic function. Front Immunol (2013) 4:316. doi: 10.3389/fimmu.2013.00316 24109481 PMC3791471

[43] Palmer S Albergante L Blackburn CC Newman TJ. Thymic involution and rising disease incidence with age. Proc Natl Acad Sci USA (2018) 115(8):1883–8. doi: 10.1073/pnas.1714478115 PMC5828591 29432166

[44] Thyagarajan B Faul J Vivek S Kim JK Nikolich-Zugich J Weir D. Age-related differences in T-cell subsets in a nationally representative sample of people older than age 55: findings from the health and retirement study. J Gerontol A Biol Sci Med Sci (2022) 77(5):927–33. doi: 10.1093/gerona/glab300 PMC9071411 34633448

[45] Qi Q Liu Y Cheng Y Glanville J Zhang D Lee JY. Diversity and clonal selection in the human T-cell repertoire. Proc Natl Acad Sci USA (2014) 111(36):13139–44. doi: 10.1073/pnas.1409155111 PMC4246948 25157137

[46] Naumova EN Yassai MB Demos W Reed E Unruh M Haribhai D. Age-based dynamics of a stable circulating Cd8 T cell repertoire component. Front Immunol (2019) 10:1717. doi: 10.3389/fimmu.2019.01717 31447830 PMC6691812

[47] Petrova G Ferrante A Gorski J. Cross-reactivity of T cells and its role in the immune system. Crit Rev Immunol (2012) 32(4):349–72. doi: 10.1615/CritRevImmunol.v32.i4.50 PMC3595599 23237510

[48] Felix NJ Donermeyer DL Horvath S Walters JJ Gross ML Suri A. Alloreactive T cells respond specifically to multiple distinct peptide-MHC complexes. Nat Immunol (2007) 8(4):388–97. doi: 10.1038/ni1446 17322886

[49] Wooldridge L Ekeruche-Makinde J van den Berg HA Skowera A Miles JJ Tan MP. A single autoimmune T cell receptor recognizes more than a million different peptides. J Biol Chem (2012) 287(2):1168–77. doi: 10.1074/jbc.M111.289488 PMC3256900 22102287

[50] Marty R Kaabinejadian S Rossell D Slifker MJ van de Haar J Engin HB. MHC-I genotype restricts the oncogenic mutational landscape. Cell (2017) 171(6):1272–83 e15. doi: 10.1016/j.cell.2017.09.050 29107334 PMC5711564

[51] Paul S Weiskopf D Angelo MA Sidney J Peters B Sette A. HLA class I alleles are associated with peptide-binding repertoires of different size, affinity, and immunogenicity. J Immunol (2013) 191(12):5831–9. doi: 10.4049/jimmunol.1302101 PMC3872965 24190657

[52] Chowell D Krishna C Pierini F Makarov V Rizvi NA Kuo F. Evolutionary divergence of HLA class I genotype impacts efficacy of cancer immunotherapy. Nat Med (2019) 25(11):1715–20. doi: 10.1038/s41591-019-0639-4 PMC7938381 31700181

[53] Chowell D Morris LGT Grigg CM Weber JK Samstein RM Makarov V. Patient HLA class I genotype influences cancer response to checkpoint blockade immunotherapy. Science (2018) 359(6375):582–7. doi: 10.1126/science.aao4572 PMC6057471 29217585

[54] Manczinger M Koncz B Balogh GM Papp BT Asztalos L Kemeny L. Negative trade-off between neoantigen repertoire breadth and the specificity of HLA-I molecules shapes antitumor immunity. Nat Cancer (2021) 2(9):950–61. doi: 10.1038/s43018-021-00226-4 35121862

[55] Petrova GV Naumov YN Naumova EN Gorski J. Role of cross-reactivity in cellular immune targeting of influenza a M1(58-66) variant peptide epitopes. Front Immunol (2022) 13:956103. doi: 10.3389/fimmu.2022.956103 36211433 PMC9539824

[56] Dunn GP Bruce AT Ikeda H Old LJ Schreiber RD. Cancer immunoediting: from immunosurveillance to tumor escape. Nat Immunol (2002) 3(11):991–8. doi: 10.1038/ni1102-991 12407406

[57] Schreiber RD Old LJ Smyth MJ. Cancer immunoediting: integrating immunity's roles in cancer suppression and promotion. Science (2011) 331(6024):1565–70. doi: 10.1126/science.1203486 21436444

[58] Matsushita H Vesely MD Koboldt DC Rickert CG Uppaluri R Magrini VJ. Cancer exome analysis reveals a T-cell-dependent mechanism of cancer immunoediting. Nature (2012) 482(7385):400–4. doi: 10.1038/nature10755 PMC3874809 22318521

[59] Yarmarkovich M Farrel A Sison A 3rd di Marco M Raman P Parris JL. Immunogenicity and immune silence in human cancer. Front Immunol (2020) 11:69. doi: 10.3389/fimmu.2020.00069 32256484 PMC7092187

[60] McGranahan N Rosenthal R Hiley CT Rowan AJ Watkins TBK Wilson GA. Allele-specific HLA loss and immune escape in lung cancer evolution. Cell (2017) 171(6):1259–71 e11. doi: 10.1016/j.cell.2017.10.001 29107330 PMC5720478

[61] Zhou C Tuong ZK Frazer IH. Papillomavirus immune evasion strategies target the infected cell and the local immune system. Front Oncol (2019) 9:682. doi: 10.3389/fonc.2019.00682 31428574 PMC6688195

[62] Oliveira G Stromhaug K Cieri N Iorgulescu JB Klaeger S Wolff JO. Landscape of helper and regulatory antitumour CD4(+) T cells in melanoma. Nature (2022) 605(7910):532–8. doi: 10.1038/s41586-022-04682-5 PMC9815755 35508657

[63] Chen DS Mellman I. Elements of cancer immunity and the cancer-immune set point. Nature (2017) 541(7637):321–30. doi: 10.1038/nature21349 28102259

[64] Philip M Schietinger A. CD8(+) T cell differentiation and dysfunction in cancer. Nat Rev Immunol (2022) 22(4):209–23. doi: 10.1038/s41577-021-00574-3 PMC9792152 34253904

[65] McGranahan N Swanton C. Cancer evolution constrained by the immune microenvironment. Cell (2017) 170(5):825–7. doi: 10.1016/j.cell.2017.08.012 28841415

[66] Joyce JA Fearon DT. T Cell exclusion, immune privilege, and the tumor microenvironment. Science (2015) 348(6230):74–80. doi: 10.1126/science.aaa6204 25838376

[67] Rosenthal R Cadieux EL Salgado R Bakir MA Moore DA Hiley CT. Neoantigen-directed immune escape in lung cancer evolution. Nature (2019) 567(7749):479–85. doi: 10.1038/s41586-019-1032-7 PMC6954100 30894752

[68] Bianchini R Kragiannis SN Jordakieva G Jensen-Jarolim E. The role of IgG4 in the fine tuning of tolerance in IgE-mediated allergy and cancer. Int J Mol Sci (2020) 21(14):5017. doi: 10.3390/ijms21145017 32708690 PMC7404042

[69] Vogelstein B Papadopoulos N Velculescu VE Zhou S Diaz LA Jr. Kinzler KW. Cancer genome landscapes. Science (2013) 339(6127):1546–58. doi: 10.1126/science.1235122 PMC3749880 23539594

[70] Grossman RL Heath AP Ferretti V Varmus HE Lowy DR Kibbe WA. Toward a shared vision for cancer genomic data. New Engl J Med (2016) 375(12):1109–12. doi: 10.1056/NEJMp1607591 PMC6309165 27653561

[71] UniProt Consortium. UniProt: the universal protein knowledgebase in 2021. Nucleic Acids Res (2021) 49(D1):D480–D9. doi: 10.1093/nar/gkaa1100 PMC7778908 33237286

[72] Jones MC Pewsey A. Sinh-arcsinh distributions. Biometrika (2009) 96(4):761–80. doi: 10.1093/biomet/asp053

[73] Human Microbiome Project C. A framework for human microbiome research. Nature (2012) 486(7402):215–21. doi: 10.1038/nature11209 PMC3377744 22699610

[74] Homan EJ Bremel RD. Patterns of predicted T-cell epitopes associated with antigenic drift in influenza H3N2 hemagglutinin. PLoSOne (2011) 6(10):e26711. doi: 10.1371/journal.pone.0026711 PMC3200361 22039539

[75] Bremel RD Homan EJ. An integrated approach to epitope analysis II: a system for proteomic-scale prediction of immunological characteristics. Immunome Res (2010) 6(1):8. doi: 10.1186/1745-7580-6-8 21044290 PMC2991286

[76] Bremel RD Homan EJ. An integrated approach to epitope analysis I: dimensional reduction, visualization and prediction of MHC binding using amino acid principal components and regression approaches. Immunome Res (2010) 6:7. doi: 10.1186/1745-7580-6-7 21044289 PMC2990731

[77] Wold S Sjorstrom M Eriksson L. PLS-regression: a basic tool of chemometrics. Chemometrics Intelligent Lab Systems (2001) 58:109–30. doi: 10.1016/S0169-7439(01)00155-1

[78] Hastie T Tibshirani R Friedman J. The elements of statistical learning: data mining, inference and prediction. 2nd ed. New York, N.Y.: Springer (2008). p. 745.

[79] Vita R Mahajan S Overton JA Dhanda SK Martini S Cantrell JR. The immune epitope database (IEDB): 2018 update. Nucleic Acids Res (2019) 47(D1):D339–D43. doi: 10.1093/nar/gky1006 PMC6324067 30357391

[80] Carney JG Cunningham P eds. The NeuralBAG algorithm: optimizing generalization performance in bagged neural networks. Bruges, Belgium: European Symposium on Artificial Neural Networks (1999).

[81] Breiman L. Using adaptive bagging to DeBias regressions. Mach Learning (2001) 45:261–77. doi: 10.1023/A:1017934522171

[82] McKenna A Hanna M Banks E Sivachenko A Cibulskis K Kernytsky A. The genome analysis toolkit: a MapReduce framework for analyzing next-generation DNA sequencing data. Genome Res (2010) 20(9):1297–303. doi: 10.1101/gr.107524.110 PMC2928508 20644199

[83] Van der Auwera GA Carneiro MO Hartl C Poplin R Del Angel G Levy-Moonshine A. From FastQ data to high confidence variant calls: the genome analysis toolkit best practices pipeline. Curr Protoc Bioinf (2013) 43:11 0 1– 0 33. doi: 10.1002/0471250953.bi1110s43 PMC4243306 25431634

[84] Boratyn GM Thierry-Mieg J Thierry-Mieg D Busby B Madden TL. Magic-BLAST, an accurate RNA-seq aligner for long and short reads. BMC Bioinf (2019) 20(1):405. doi: 10.1186/s12859-019-2996-x PMC6659269 31345161

[85] Lo W Parkhurst M Robbins PF Tran E Lu YC Jia L. Immunologic recognition of a shared p53 mutated neoantigen in a patient with metastatic colorectal cancer. Cancer Immunol Res (2019) 7(4):534–43. doi: 10.1158/2326-6066.CIR-18-0686 PMC6685528 30709841

[86] Malekzadeh P Yossef R Cafri G Paria BC Lowery FJ Jafferji M. Antigen experienced T cells from peripheral blood recognize p53 neoantigens. Clin Cancer Res (2020) 26(6):1267–76. doi: 10.1158/1078-0432.CCR-19-1874 PMC7424598 31996390

[87] Capietto AH Jhunjhunwala S Pollock SB Lupardus P Wong J Hansch L. Mutation position is an important determinant for predicting cancer neoantigens. J Exp Med (2020) 217(4):e20190179. doi: 10.1084/jem.20190179 31940002 PMC7144530

[88] Yadav M Jhunjhunwala S Phung QT Lupardus P Tanguay J Bumbaca S. Predicting immunogenic tumour mutations by combining mass spectrometry and exome sequencing. Nature (2014) 515(7528):572–6. doi: 10.1038/nature14001 25428506

[89] Bassani-Sternberg M Pletscher-Frankild S Jensen LJ Mann M. Mass spectrometry of human leukocyte antigen class I peptidomes reveals strong effects of protein abundance and turnover on antigen presentation. Mol Cell Proteomics MCP (2015) 14(3):658–73. doi: 10.1074/mcp.M114.042812 PMC4349985 25576301

[90] Lang F Schrors B Lower M Tureci O Sahin U. Identification of neoantigens for individualized therapeutic cancer vaccines. Nat Rev Drug Discov (2022) 21(4):261–82. doi: 10.1038/s41573-021-00387-y PMC7612664 35105974

[91] Meredith M Zemmour D Mathis D Benoist C. Aire controls gene expression in the thymic epithelium with ordered stochasticity. Nat Immunol (2015) 16(9):942–9. doi: 10.1038/ni.3247 PMC4632529 26237550

[92] Sasaki K Takada K Ohte Y Kondo H Sorimachi H Tanaka K. Thymoproteasomes produce unique peptide motifs for positive selection of CD8(+) T cells. Nat Commun (2015) 6:7484. doi: 10.1038/ncomms8484 26099460 PMC4557289

[93] Murata S Takahama Y Kasahara M Tanaka K. The immunoproteasome and thymoproteasome: functions, evolution and human disease. Nat Immunol (2018) 19(9):923–31. doi: 10.1038/s41590-018-0186-z 30104634

[94] Honey K Rudensky AY. Lysosomal cysteine proteases regulate antigen presentation. Nat Rev Immunol (2003) 3(6):472–82. doi: 10.1038/nri1110 12776207

[95] Gommeaux J Gregoire C Nguessan P Richelme M Malissen M Guerder S. Thymus-specific serine protease regulates positive selection of a subset of CD4+ thymocytes. Eur J Immunol (2009) 39(4):956–64. doi: 10.1002/eji.200839175 19283781

[96] D'Souza MP Adams E Altman JD Birnbaum ME Boggiano C Casorati G. Casting a wider net: immunosurveillance by nonclassical MHC molecules. PloS Pathogens (2019) 15(2):e1007567. doi: 10.1371/journal.ppat.1007567 30789961 PMC6383864

[97] Imbert C Olive D. Gammadelta T cells in tumor microenvironment. Adv Exp Med Biol (2020) 1273:91–104. doi: 10.1007/978-3-030-49270-0_5 33119877

[98] Matson V Fessler J Bao R Chongsuwat T Zha Y Alegre ML. The commensal microbiome is associated with anti-PD-1 efficacy in metastatic melanoma patients. Science (2018) 359(6371):104–8. doi: 10.1126/science.aao3290 PMC6707353 29302014

[99] Wucherpfennig KW Allen PM Celada F Cohen IR De Boer R Garcia KC. Polyspecificity of T cell and b cell receptor recognition. Semin Immunol (2007) 19(4):216–24. doi: 10.1016/j.smim.2007.02.012 PMC2034306 17398114

[100] Mason D. A very high level of crossreactivity is an essential feature of the T-cell receptor. Immunol Today (1998) 19(9):395–404. doi: 10.1016/S0167-5699(98)01299-7 9745202

[101] Leng Q Tarbe M Long Q Wang F. Pre-existing heterologous T-cell immunity and neoantigen immunogenicity. Clin Trans Immunol (2020) 9(3):e01111. doi: 10.1002/cti2.1111 PMC7085466 32211191

[102] Naumova EN Naumov YN Gorski J. Measuring immunological age: from T cell repertoires to populations. In: Fulop T, editor. Handbook of immunosenesence. New York, N.Y.: Springer International Publishing (2018).

[103] Doherty PC Zinkernagel RM. Enhanced immunological surveillance in mice heterozygous at the h-2 gene complex. Nature (1975) 256(5512):50–2. doi: 10.1038/256050a0 1079575

[104] Abed A Calapre L Lo J Correia S Bowyer S Chopra A. Prognostic value of HLA-I homozygosity in patients with non-small cell lung cancer treated with single agent immunotherapy. J Immunother Cancer (2020) 8(2):e001620. doi: 10.1136/jitc-2020-001620 33229510 PMC7684824

[105] Lee D Park J Choi H Gim G Cho S Kim L. Association of HLA class I homozygosity with unfavorable clinical outcomes in patients with non-small cell lung cancer treated with chemo-immunotherapy or immunotherapy as first-line therapy. Heliyon (2021) 7(9):e07916. doi: 10.1016/j.heliyon.2021.e07916 34568594 PMC8449023

[106] Abelin JG Keskin DB Sarkizova S Hartigan CR Zhang W Sidney J. Mass spectrometry profiling of HLA-associated peptidomes in mono-allelic cells enables more accurate epitope prediction. Immunity (2017) 46(2):315–26. doi: 10.1016/j.immuni.2017.02.007 PMC5405381 28228285

[107] Balachandran VP Luksza M Zhao JN Makarov V Moral JA Remark R. Identification of unique neoantigen qualities in long-term survivors of pancreatic cancer. Nature (2017) 551(7681):512–6. doi: 10.1038/nature24462 PMC6145146 29132146

[108] Hoyos D Zappasodi R Schulze I Sethna Z de Andrade KC Bajorin DF. Fundamental immune-oncogenicity trade-offs define driver mutation fitness. Nature (2022) 606(7912):172–9. doi: 10.1038/s41586-022-04696-z PMC9159948 35545680

[109] Luksza M Riaz N Makarov V Balachandran VP Hellmann MD Solovyov A. A neoantigen fitness model predicts tumour response to checkpoint blockade immunotherapy. Nature (2017) 551(7681):517–20. doi: 10.1038/nature24473 PMC6137806 29132144

[110] Luksza M Sethna ZM Rojas LA Lihm J Bravi B Elhanati Y. Neoantigen quality predicts immunoediting in survivors of pancreatic cancer. Nature (2022) 606(7913):389–95. doi: 10.1038/s41586-022-04735-9 PMC9177421 35589842

[111] Wucherpfennig KW. The structural interactions between T cell receptors and MHC-peptide complexes place physical limits on self-nonself discrimination. Curr Topics Microbiol Immunol (2005) 296:19–37. doi: 10.1007/3-540-30791-5_2 16329190

[112] Ghorani E Rosenthal R McGranahan N Reading JL Lynch M Peggs KS. Differential binding affinity of mutated peptides for MHC class I is a predictor of survival in advanced lung cancer and melanoma. Ann Oncol (2018) 29(1):271–9. doi: 10.1093/annonc/mdx687 PMC5834109 29361136

[113] Cullis J Das S Bar-Sagi D. Kras and tumor immunity: friend or foe? Cold Spring Harb Perspect Med (2018) 8(9):a031849. doi: 10.1101/cshperspect.a031849 29229670 PMC6120695

[114] Tran E Robbins PF Lu YC Prickett TD Gartner JJ Jia L. T-Cell transfer therapy targeting mutant KRAS in cancer. New Engl J Med (2016) 375(23):2255–62. doi: 10.1056/NEJMoa1609279 PMC5178827 27959684

[115] Song I Gil A Mishra R Ghersi D Selin LK Stern LJ. Broad TCR repertoire and diverse structural solutions for recognition of an immunodominant CD8(+) T cell epitope. Nat Struct Mol Biol (2017) 24(4):395–406. doi: 10.1038/nsmb.3383 28250417 PMC5383516

[116] Gilbert JA Quinn RA Debelius J Xu ZZ Morton J Garg N. Microbiome-wide association studies link dynamic microbial consortia to disease. Nature (2016) 535(7610):94–103. doi: 10.1038/nature18850 27383984

[117] Pinato DJ Howlett S Ottaviani D Urus H Patel A Mineo T. Association of prior antibiotic treatment with survival and response to immune checkpoint inhibitor therapy in patients with cancer. JAMA Oncol (2019) 5(12):1774–8. doi: 10.1001/jamaoncol.2019.2785 PMC6743060 31513236

[118] Hanahan D Weinberg RA. Hallmarks of cancer: the next generation. Cell (2011) 144(5):646–74. doi: 10.1016/j.cell.2011.02.013 21376230

[119] Alexandrov LB Nik-Zainal S Wedge DC Aparicio SA Behjati S Biankin AV. Signatures of mutational processes in human cancer. Nature (2013) 500(7463):415–21. doi: 10.1038/nature12477 PMC3776390 23945592

[120] Degasperi A Zou X Amarante TD Martinez-Martinez A Koh GCC Dias JML. Substitution mutational signatures in whole-genome-sequenced cancers in the UK population. Science (2022) 376(6591):science.abl9283. doi: 10.1126/science.abl9283 35949260 PMC7613262

[121] Steele CD Abbasi A Islam SMA Bowes AL Khandekar A Haase K. Signatures of copy number alterations in human cancer. Nature (2022) 606(7916):984–91. doi: 10.1038/s41586-022-04738-6 PMC9242861 35705804

[122] Steele CD Pillay N Alexandrov LB. An overview of mutational and copy number signatures in human cancer. J Pathol (2022) 257(4):454–65. doi: 10.1002/path.5912 PMC9324981 35420163

[123] Hoglund RA Torsetnes SB Lossius A Bogen B Homan EJ Bremel R. Human cysteine cathepsins degrade immunoglobulin G in vitro in a predictable manner. Int J Mol Sci (2019) 20(19):4843. doi: 10.3390/ijms20194843 31569504 PMC6801702

[124] Biniossek ML Nagler DK Becker-Pauly C Schilling O. Proteomic identification of protease cleavage sites characterizes prime and non-prime specificity of cysteine cathepsins b, l and s. J Proteome Res (2011) 10(12):5363–73. doi: 10.1021/pr200621z 21967108

[125] Rawlings ND Waller M Barrett AJ Bateman A. MEROPS: the database of proteolytic enzymes, their substrates and inhibitors. Nucleic Acids Res (2014) 42(Database issue):D503–9. doi: 10.1093/nar/gkt953 PMC3964991 24157837

