# Supplementary material for: Determinants of tumor immune evasion: the role of T cell exposed motif frequency and mutant amino acid exposure
Source: Front Immunol. 2023 May 5;14:1155679. doi: 10.3389/fimmu.2023.1155679 (PMC10196236; doi:10.3389/fimmu.2023.1155679)
Supplement: Supplementary file 1 [file DataSheet_1.pdf]

Supplemental Table 2: Oncogenes and tumor suppressor gene products analyzed.

Uniprot ID	Gene ID	Number Mutants	Type	Uniprot ID	Gene ID	Number Mutants	Type
P00519_2	ABL1	128	Oncogene	Q13315	ATM	450	TSG
P31749	AKT1	50	Oncogene	P46100	ATRX	517	TSG
Q9UM73	ALK	313	Oncogene	O15169	AXIN1	120	TSG
P10275	AR	175	Oncogene	P61769	B2M	36	TSG
P10415	BCL2	27	Oncogene	Q92560	BAP1	131	TSG
A0A2R8Y8E0	BRAF	134	Oncogene	Q6W2J9	BCOR	248	TSG
Q9BXL7	CARD11	286	Oncogene	P38398_7	BRCA1	213	TSG
P22681	CBL	110	Oncogene	P51587	BRCA2	463	TSG
Q9HC73	CRLF2	47	Oncogene	Q14790__9	CASP8	147	TSG
P07333	CSF1R	149	Oncogene	Q6P1J9	CDC73	116	TSG
P35222	CTNNB1	164	Oncogene	P42771_4	CDKN2A	103	TSG
P26358	DNMT1	198	Oncogene	P49715	CEBPA	17	TSG
Q9Y6K1	DNMT3A	162	Oncogene	I3L2J0	CIC	211	TSG
P00533	EGFR	270	Oncogene	Q92793	CREBBP	403	TSG
P04626	ERBB2	152	Oncogene	Q9NQC7	CYLD	142	TSG
Q15910_2	EZH2	119	Oncogene	Q9UER7	DAXX	114	TSG
P21802_3	FGFR2	171	Oncogene	Q09472	EP300	332	TSG
P22607_3	FGFR3	105	Oncogene	Q969H0	FBXW7	156	TSG
P36888	FLT3	203	Oncogene	Q96AE4	FUBP1	84	TSG
P58012	FOXL2	41	Oncogene	P15976	GATA1	65	TSG
P23769	GATA2	61	Oncogene	P23771_2	GATA3	117	TSG
P29992	GNA11	50	Oncogene	P20823	HNF1A	100	TSG
P50148	GNAQ	55	Oncogene	P41229	KDM5C	206	TSG
Q5JWF2	GNAS	178	Oncogene	A0A087X0R0	KDM6A	173	TSG
P84243	H3-3A	29	Oncogene	Q8NEZ4	KMT2C	723	TSG
P68431	H3C2	57	Oncogene	O14686	KMT2D	728	TSG
P01112	HRAS	40	Oncogene	Q13233	MAP3K1	180	TSG
O75874	IDH1	62	Oncogene	O00255	MEN1	75	TSG
P48735	IDH2	54	Oncogene	P40692	MLH1	98	TSG
P23458	JAK1	159	Oncogene	P43246	MSH2	107	TSG
O60674	JAK2	151	Oncogene	P52701	MSH6	171	TSG
P52333	JAK3	156	Oncogene	O75376	NCOR1	326	TSG
P10721	KIT	213	Oncogene	P21359	NF1	379	TSG
O43474_1	KLF4	66	Oncogene	P35240	NF2	74	TSG
P01116	KRAS	61	Oncogene	P46531	NOTCH1	371	TSG
Q02750	MAP2K1	65	Oncogene	Q04721	NOTCH2	300	TSG
Q93074	MED12	392	Oncogene	A0A7I2YQC0	NPM1	31	TSG
P08581_2	MET	218	Oncogene	Q02548	PAX5	90	TSG

Supplemental Figure 1: Peptide numbering convention

Alignment relative to mutant peptide index position.
TCEM highlighted

-10	LLWFAHHSL	LLWFAHHSLFPHVRK
-9	LWFAHHSLF	LWFAHHSLFPHVRKA
-8	WFAHHSLFP	WFAHHSLFPHVRKAT
-7	FAHHSLFPH	FAHHSLFPHVRKATR
-6	AHHSLFPHV	AHHSLFPHVRKATRA
-5	HHSLFPHVR	HHSLFPHVRKATRAM
-4	HSLFPHVRK	HSLFPHVRKATRAMG
-3	SLFPHVRKA	SLFPHVRKATRAMGL
-2	LFPHVRKAT	LFPHVRKATRAMGLL
-1	FPHVRKATR	FPHVRKATRAMGLLN
0	PHVRKATRA	PHVRKATRAMGLLNT
1	HVRKATRAM	HVRKATRAMGLLNTL
2	VRKATRAMG	VRKATRAMGLLNTLS
3	RKATRAMGL	RKATRAMGLLNTLSL

Supplemental File 2A Oncogenes and Suppressors Poisson Distributions of TCEM and GEM frequency: Relative to Hg38 Human proteome

MHC I

GEM

hg38 maxi_I mut

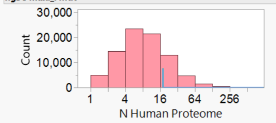

Summary Statistics	
Mean	15.93
Std Dev	83.63
Std Err Mean	0.29
Upper 95% Mean	16.50
Lower 95% Mean	15.36
N	82567.00
Skewness	25.98
Kurtosis	758.51
N Missing	0.00
N Zero	19.00
Interquartile Range	10.00
Proportion Zero	0.00

Fitted ZI Poisson Distribution

Parameter		Estimate	Std Error	Lower 95%	Upper 95%
Mean	λ	15.93	0.01	15.91	15.96
Zero Inflation	π	0.00	0.00	0.00	0.00

Measures	
-2*LogLikelihood	3633795.9
AICc	3633799.9
BIC	3633818.5

Fitted Poisson Distribution

Parameter		Estimate	Std Error	Lower 95%	Upper 95%
Mean	λ	15.93	0.01	15.90	15.96

Measures	
-2*LogLikelihood	3634044.9
AICc	3634046.9
BIC	3634056.2

hg38 maxi_I wt

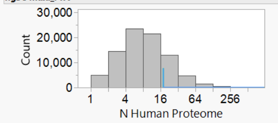

Summary Statistics	
Mean	15.98
Std Dev	84.38
Std Err Mean	0.29
Upper 95% Mean	16.55
Lower 95% Mean	15.40
N	82567.00
Skewness	25.83
Kurtosis	748.73
N Missing	0.00
N Zero	19.00
Interquartile Range	10.00
Proportion Zero	0.00

Fitted ZI Poisson Distribution

Parameter		Estimate	Std Error	Lower 95%	Upper 95%
Mean	λ	15.98	0.01	15.95	16.01
Zero Inflation	π	0.00	0.00	0.00	0.00

Measures	
-2*LogLikelihood	3667485.8
AICc	3667489.8
BIC	3667508.4

Fitted Poisson Distribution

Parameter		Estimate	Std Error	Lower 95%	Upper 95%
Mean	λ	15.98	0.01	15.95	16.01

Measures	
-2*LogLikelihood	3667736.7
AICc	3667738.7
BIC	3667748.0

TCEM

hg38 maxi_I mut

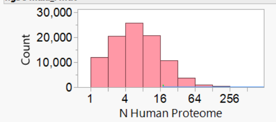

Summary Statistics	
Mean	9.05
Std Dev	36.57
Std Err Mean	0.11
Upper 95% Mean	9.27
Lower 95% Mean	8.83
N	103212.00
Skewness	55.99
Kurtosis	3866.95
N Missing	0.00
N Zero	10396.00
Interquartile Range	8.00
Proportion Zero	0.10

Fitted ZI Poisson Distribution

Parameter		Estimate	Std Error	Lower 95%	Upper 95%
Mean	λ	10.06	0.01	10.04	10.08
Zero Inflation	π	0.10	0.00	0.10	0.10

Measures	
-2*LogLikelihood	1896567.6
AICc	1896571.6
BIC	1896590.6

Fitted Poisson Distribution

Parameter		Estimate	Std Error	Lower 95%	Upper 95%
Mean	λ	9.05	0.01	9.03	9.07

Measures	
-2*LogLikelihood	2027453.0
AICc	2027455.0
BIC	2027464.6

hg38 maxi_I wt

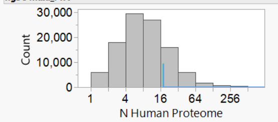

Summary Statistics	
Mean	15.78
Std Dev	83.08
Std Err Mean	0.26
Upper 95% Mean	16.29
Lower 95% Mean	15.27
N	103212.00
Skewness	27.48
Kurtosis	838.53
N Missing	0.00
N Zero	6.00
Interquartile Range	11.00
Proportion Zero	0.00

Fitted ZI Poisson Distribution

Parameter		Estimate	Std Error	Lower 95%	Upper 95%
Mean	λ	15.78	0.01	15.76	15.81
Zero Inflation	π	0.00	0.00	0.00	0.00

Measures	
-2*LogLikelihood	4403377.8
AICc	4403381.8
BIC	4403400.9

Fitted Poisson Distribution

Parameter		Estimate	Std Error	Lower 95%	Upper 95%
Mean	λ	15.78	0.01	15.76	15.81

Measures	
-2*LogLikelihood	4403438.2
AICc	4403440.2
BIC	4403449.7

MHC II

GEM

hg38 maxi_IIa mut

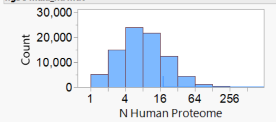

Summary Statistics	
Mean	13.11
Std Dev	45.72
Std Err Mean	0.16
Upper 95% Mean	13.42
Lower 95% Mean	12.80
N	82610.00
Skewness	30.46
Kurtosis	1136.79
N Missing	0.00
N Zero	22.00
Interquartile Range	10.00
Proportion Zero	0.00

Fitted ZI Poisson Distribution

Parameter		Estimate	Std Error	Lower 95%	Upper 95%
Mean	λ	13.11	0.01	13.09	13.14
Zero Inflation	π	0.00	0.00	0.00	0.00

Measures	
-2*LogLikelihood	2143100.1
AICc	2143104.1
BIC	2143122.8

Fitted Poisson Distribution

Parameter		Estimate	Std Error	Lower 95%	Upper 95%
Mean	λ	13.11	0.01	13.09	13.13

Measures	
-2*LogLikelihood	2143271.2
AICc	2143273.2
BIC	2143282.5

hg38 maxi_IIa wt

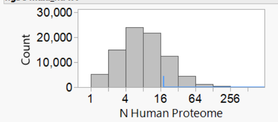

Summary Statistics	
Mean	13.15
Std Dev	46.14
Std Err Mean	0.16
Upper 95% Mean	13.46
Lower 95% Mean	12.83
N	82610.00
Skewness	30.09
Kurtosis	1107.51
N Missing	0.00
N Zero	22.00
Interquartile Range	10.00
Proportion Zero	0.00

Fitted ZI Poisson Distribution

Parameter		Estimate	Std Error	Lower 95%	Upper 95%
Mean	λ	13.15	0.01	13.13	13.18
Zero Inflation	π	0.00	0.00	0.00	0.00

Measures	
-2*LogLikelihood	2164036.4
AICc	2164040.4
BIC	2164059.1

Fitted Poisson Distribution

Parameter		Estimate	Std Error	Lower 95%	Upper 95%
Mean	λ	13.15	0.01	13.12	13.17

Measures	
-2*LogLikelihood	2164209.2
AICc	2164211.2
BIC	2164220.5

TCEM

hg38 maxi_IIa mut

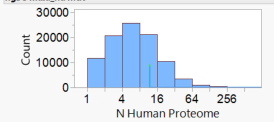

Summary Statistics	
Mean	8.68
Std Dev	23.62
Std Err Mean	0.07
Upper 95% Mean	8.83
Lower 95% Mean	8.54
N	103255.00
Skewness	46.50
Kurtosis	3147.25
N Missing	0.00
N Zero	10229.00
Interquartile Range	8.00
Proportion Zero	0.10

Fitted ZI Poisson Distribution

Parameter		Estimate	Std Error	Lower 95%	Upper 95%
Mean	λ	9.64	0.01	9.62	9.66
Zero Inflation	π	0.10	0.00	0.10	0.10

Measures	
-2*LogLikelihood	1585265.3
AICc	1585269.3
BIC	1585288.4

Fitted Poisson Distribution

Parameter		Estimate	Std Error	Lower 95%	Upper 95%
Mean	λ	8.68	0.01	8.67	8.70

Measures	
-2*LogLikelihood	1705644.7
AICc	1705646.7
BIC	1705656.3

hg38 maxi_IIa wt

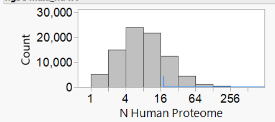

Summary Statistics	
Mean	13.15
Std Dev	46.14
Std Err Mean	0.16
Upper 95% Mean	13.46
Lower 95% Mean	12.83
N	82610.00
Skewness	30.09
Kurtosis	1107.51
N Missing	0.00
N Zero	22.00
Interquartile Range	10.00
Proportion Zero	0.00

Fitted ZI Poisson Distribution

Parameter		Estimate	Std Error	Lower 95%	Upper 95%
Mean	λ	13.15	0.01	13.13	13.18
Zero Inflation	π	0.00	0.00	0.00	0.00

Measures	
-2*LogLikelihood	2164036.4
AICc	2164040.4
BIC	2164059.1

Fitted Poisson Distribution

Parameter		Estimate	Std Error	Lower 95%	Upper 95%
Mean	λ	13.15	0.01	13.12	13.17

Measures	
-2*LogLikelihood	2164209.2
AICc	2164211.2
BIC	2164220.5

Supplemental File 2B Oncogenes and Suppressors Poisson Distributions of TCEM and GEM frequency: Relative to GI microbiome

MHC I

GEM

N TCEM_I (4,5,6,7,8) GI mut

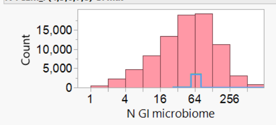

Summary Statistics	
Mean	78.14
Std Dev	101.26
Std Err Mean	0.35
Upper 95% Mean	78.83
Lower 95% Mean	77.45
N	82567.00
Skewness	5.89
Kurtosis	92.31
N Missing	0.00
N Zero	1552.00
Interquartile Range	81.00
Proportion Zero	0.02

Fitted ZI Poisson Distribution

Parameter		Estimate	Std Error	Lower 95%	Upper 95%
Mean	λ	79.64	0.03	79.58	79.70
Zero Inflation	π	0.02	0.00	0.02	0.02

Measures	
-2*LogLikelihood	7099836.2
AICc	7099840.2
BIC	7099858.8

Fitted Poisson Distribution

Parameter		Estimate	Std Error	Lower 95%	Upper 95%
Mean	λ	78.14	0.03	78.08	78.20

Measures	
-2*LogLikelihood	7329282.9
AICc	7329284.9
BIC	7329294.3

N TCEM_I (4,5,6,7,8) GI wt

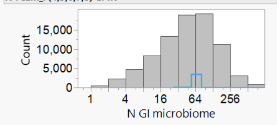

Summary Statistics	
Mean	78.15
Std Dev	101.27
Std Err Mean	0.35
Upper 95% Mean	78.84
Lower 95% Mean	77.46
N	82567.00
Skewness	5.89
Kurtosis	92.26
N Missing	0.00
N Zero	1552.00
Interquartile Range	81.00
Proportion Zero	0.02

Fitted ZI Poisson Distribution

Parameter		Estimate	Std Error	Lower 95%	Upper 95%
Mean	λ	79.64	0.03	79.58	79.71
Zero Inflation	π	0.02	0.00	0.02	0.02

Measures	
-2*LogLikelihood	7101004.1
AICc	7101008.1
BIC	7101026.7

Fitted Poisson Distribution

Parameter		Estimate	Std Error	Lower 95%	Upper 95%
Mean	λ	78.15	0.03	78.09	78.21

Measures	
-2*LogLikelihood	7330473.0
AICc	7330475.0
BIC	7330484.3

TCEM

N TCEM_I (4,5,6,7,8) GI mut

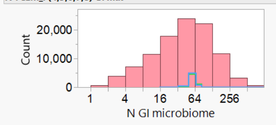

Summary Statistics	
Mean	66.82
Std Dev	83.50
Std Err Mean	0.26
Upper 95% Mean	67.32
Lower 95% Mean	66.31
N	103212.00
Skewness	4.41
Kurtosis	51.11
N Missing	0.00
N Zero	2740.00
Interquartile Range	71.00
Proportion Zero	0.03

Fitted ZI Poisson Distribution

Parameter		Estimate	Std Error	Lower 95%	Upper 95%
Mean	λ	68.64	0.03	68.59	68.69
Zero Inflation	π	0.03	0.00	0.03	0.03

Measures	
-2*LogLikelihood	7615744.1
AICc	7615748.1
BIC	7615767.2

Fitted Poisson Distribution

Parameter		Estimate	Std Error	Lower 95%	Upper 95%
Mean	λ	66.82	0.03	66.77	66.87

Measures	
-2*LogLikelihood	7961547.8
AICc	7961549.8
BIC	7961559.4

N TCEM_I (4,5,6,7,8) GI wt

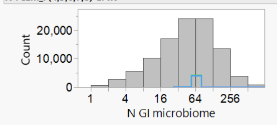

Summary Statistics	
Mean	76.76
Std Dev	98.33
Std Err Mean	0.31
Upper 95% Mean	77.36
Lower 95% Mean	76.16
N	103212.00
Skewness	6.46
Kurtosis	139.34
N Missing	0.00
N Zero	2015.00
Interquartile Range	78.00
Proportion Zero	0.02

Fitted ZI Poisson Distribution

Parameter		Estimate	Std Error	Lower 95%	Upper 95%
Mean	λ	78.29	0.03	78.24	78.35
Zero Inflation	π	0.02	0.00	0.02	0.02

Measures	
-2*LogLikelihood	8619449.2
AICc	8619453.2
BIC	8619472.3

Fitted Poisson Distribution

Parameter		Estimate	Std Error	Lower 95%	Upper 95%
Mean	λ	76.76	0.03	76.71	76.82

Measures	
-2*LogLikelihood	8912010.1
AICc	8912012.1
BIC	8912021.6

MHC II

GEM

N TCEM_IIa (2,3,5,7,8) GI mut

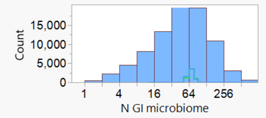

Summary Statistics	
Mean	75.88
Std Dev	92.11
Std Err Mean	0.32
Upper 95% Mean	76.50
Lower 95% Mean	75.25
N	82610.00
Skewness	4.30
Kurtosis	38.43
N Missing	0.00
N Zero	1449.00
Interquartile Range	78.00
Proportion Zero	0.02

Fitted ZI Poisson Distribution

Parameter		Estimate	Std Error	Lower 95%	Upper 95%
Mean	λ	77.23	0.03	77.17	77.29
Zero Inflation	π	0.02	0.00	0.02	0.02

Measures	
-2*LogLikelihood	6546561.6
AICc	6546565.6
BIC	6546584.3

Fitted Poisson Distribution

Parameter		Estimate	Std Error	Lower 95%	Upper 95%
Mean	λ	75.88	0.03	75.82	75.93

Measures	
-2*LogLikelihood	6753809.6
AICc	6753811.6
BIC	6753821.0

N TCEM_IIa (2,3,5,7,8) GI wt

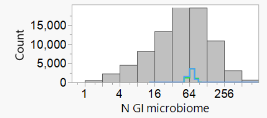

Summary Statistics	
Mean	75.88
Std Dev	92.12
Std Err Mean	0.32
Upper 95% Mean	76.51
Lower 95% Mean	75.25
N	82610.00
Skewness	4.29
Kurtosis	38.42
N Missing	0.00
N Zero	1449.00
Interquartile Range	78.00
Proportion Zero	0.02

Fitted ZI Poisson Distribution

Parameter		Estimate	Std Error	Lower 95%	Upper 95%
Mean	λ	77.23	0.03	77.17	77.29
Zero Inflation	π	0.02	0.00	0.02	0.02

Measures	
-2*LogLikelihood	6546750.1
AICc	6546754.1
BIC	6546772.7

Fitted Poisson Distribution

Parameter		Estimate	Std Error	Lower 95%	Upper 95%
Mean	λ	75.88	0.03	75.82	75.94

Measures	
-2*LogLikelihood	6754008.2
AICc	6754010.2
BIC	6754019.5

TCEM

N TCEM_IIa (2,3,5,7,8) GI mut

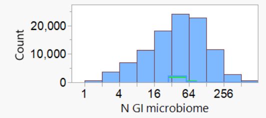

Summary Statistics	
Mean	66.09
Std Dev	79.42
Std Err Mean	0.25
Upper 95% Mean	66.58
Lower 95% Mean	65.61
N	103255.00
Skewness	4.98
Kurtosis	120.04
N Missing	0.00
N Zero	2475.00
Interquartile Range	71.00
Proportion Zero	0.02

Fitted ZI Poisson Distribution

Parameter		Estimate	Std Error	Lower 95%	Upper 95%
Mean	λ	67.71	0.03	67.66	67.77
Zero Inflation	π	0.02	0.00	0.02	0.02

Measures	
-2*LogLikelihood	7225427.9
AICc	7225431.9
BIC	7225451.0

Fitted Poisson Distribution

Parameter		Estimate	Std Error	Lower 95%	Upper 95%
Mean	λ	66.09	0.03	66.04	66.14

Measures	
-2*LogLikelihood	7533208.2
AICc	7533210.2
BIC	7533219.7

N TCEM_IIa (2,3,5,7,8) GI wt

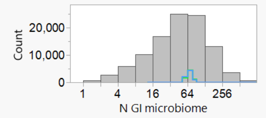

Summary Statistics	
Mean	75.36
Std Dev	91.99
Std Err Mean	0.29
Upper 95% Mean	75.92
Lower 95% Mean	74.80
N	103255.00
Skewness	4.49
Kurtosis	42.33
N Missing	0.00
N Zero	1695.00
Interquartile Range	77.00
Proportion Zero	0.02

Fitted ZI Poisson Distribution

Parameter		Estimate	Std Error	Lower 95%	Upper 95%
Mean	λ	76.62	0.03	76.57	76.67
Zero Inflation	π	0.02	0.00	0.02	0.02

Measures	
-2*LogLikelihood	8125913.7
AICc	8125917.7
BIC	8125936.8

Fitted Poisson Distribution

Parameter		Estimate	Std Error	Lower 95%	Upper 95%
Mean	λ	75.36	0.03	75.31	75.42

Measures	
-2*LogLikelihood	8366220.3
AICc	8366222.3
BIC	8366231.9

Supplemental Figure 3: Oncogenes and Suppressors Pocket specific changes – MHC I and MHC II Normalized log distributions relative to Hg38 Human proteome

MHC I Mutant red; WT grey

P1

(imputed) TCEM_I mut

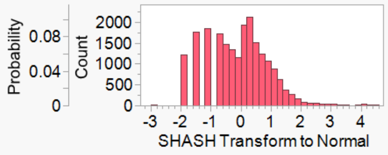

Quantiles

100.0%	maximum	4.4140226357
99.5%		2.5154515586
97.5%		1.6971998823
90.0%		1.0716014667
75.0%	quartile	0.5909916126
50.0%	median	-0.112959283
25.0%	quartile	-0.705338025
10.0%		-1.417441656
2.5%		-1.992546039
0.5%		-1.992546039
0.0%	minimum	-2.96456149

Summary Statistics

Mean	-0.101522
Std Dev	0.9683301
Std Err Mean	0.0067421
Upper 95% Mean	-0.088307
Lower 95% Mean	-0.114737
N	20628
Skewness	0.0915948
Kurtosis	0.1555622
N Missing	0
N Zero	0
Interquartile Range	1.2963296
Proportion Zero	0

(imputed) TCEM_I wt

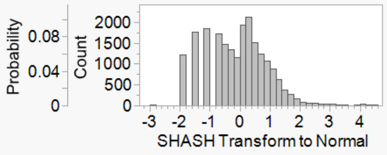

Quantiles

100.0%	maximum	4.4140226357
99.5%		2.5154515586
97.5%		1.6971998823
90.0%		1.0716014667
75.0%	quartile	0.5909916126
50.0%	median	-0.112959283
25.0%	quartile	-0.705338025
10.0%		-1.417441656
2.5%		-1.992546039
0.5%		-1.992546039
0.0%	minimum	-2.96456149

Summary Statistics

Mean	-0.101522
Std Dev	0.9683301
Std Err Mean	0.0067421
Upper 95% Mean	-0.088307
Lower 95% Mean	-0.114737
N	20628
Skewness	0.0915948
Kurtosis	0.1555622
N Missing	0
N Zero	0
Interquartile Range	1.2963296
Proportion Zero	0

P2

(imputed) TCEM_I mut

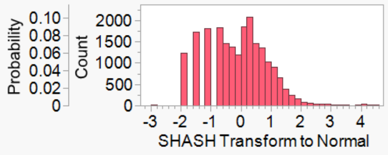

Quantiles

100.0%	maximum	4.4140226357
99.5%		2.4391642639
97.5%		1.6584159424
90.0%		1.0716014667
75.0%	quartile	0.5909916126
50.0%	median	-0.112959283
25.0%	quartile	-0.705338025
10.0%		-1.417441656
2.5%		-1.992546039
0.5%		-1.992546039
0.0%	minimum	-2.96456149

Summary Statistics

Mean	-0.10492
Std Dev	0.9621903
Std Err Mean	0.0066971
Upper 95% Mean	-0.091793
Lower 95% Mean	-0.118046
N	20642
Skewness	0.0588208
Kurtosis	0.062967
N Missing	0
N Zero	0
Interquartile Range	1.2963296
Proportion Zero	0

(imputed) TCEM_I wt

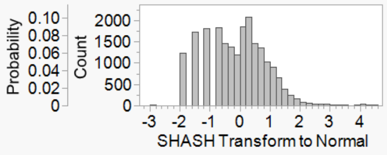

Quantiles

100.0%	maximum	4.4140226357
99.5%		2.4391642639
97.5%		1.6584159424
90.0%		1.0716014667
75.0%	quartile	0.5909916126
50.0%	median	-0.112959283
25.0%	quartile	-0.705338025
10.0%		-1.417441656
2.5%		-1.992546039
0.5%		-1.992546039
0.0%	minimum	-2.96456149

Summary Statistics

Mean	-0.10492
Std Dev	0.9621903
Std Err Mean	0.0066971
Upper 95% Mean	-0.091793
Lower 95% Mean	-0.118046
N	20642
Skewness	0.0588208
Kurtosis	0.062967
N Missing	0
N Zero	0
Interquartile Range	1.2963296
Proportion Zero	0

P3

(imputed) TCEM_I mut

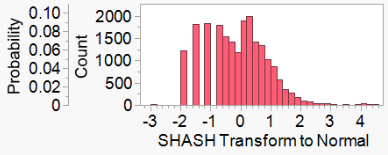

Quantiles

100.0%	maximum	4.4140226357
99.5%		2.3940452842
97.5%		1.6838749514
90.0%		1.0716014667
75.0%	quartile	0.5319239347
50.0%	median	-0.112959283
25.0%	quartile	-0.705338025
10.0%		-1.417441656
2.5%		-1.992546039
0.5%		-1.992546039
0.0%	minimum	-2.96456149

Summary Statistics

Mean	-0.119227
Std Dev	0.9605233
Std Err Mean	0.0066855
Upper 95% Mean	-0.106123
Lower 95% Mean	-0.132331
N	20642
Skewness	0.0694978
Kurtosis	-0.010163
N Missing	0
N Zero	0
Interquartile Range	1.237262
Proportion Zero	0

(imputed) TCEM_I wt

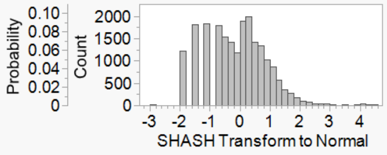

Quantiles

100.0%	maximum	4.4140226357
99.5%		2.3940452842
97.5%		1.6838749514
90.0%		1.0716014667
75.0%	quartile	0.5319239347
50.0%	median	-0.112959283
25.0%	quartile	-0.705338025
10.0%		-1.417441656
2.5%		-1.992546039
0.5%		-1.992546039
0.0%	minimum	-2.96456149

Summary Statistics

Mean	-0.119227
Std Dev	0.9605233
Std Err Mean	0.0066855
Upper 95% Mean	-0.106123
Lower 95% Mean	-0.132331
N	20642
Skewness	0.0694978
Kurtosis	-0.010163
N Missing	0
N Zero	0
Interquartile Range	1.237262
Proportion Zero	0

P4

(imputed) TCEM_I mut

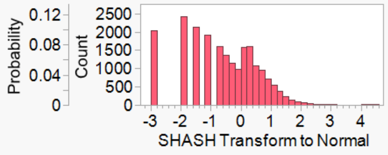

Quantiles

100.0%	maximum	4.4007077149
99.5%		1.9224549145
97.5%		1.3775482226
90.0%		0.8305853509
75.0%	quartile	0.2320725377
50.0%	median	-0.46473686
25.0%	quartile	-1.417441656
10.0%		-1.992546039
2.5%		-2.96456149
0.5%		-2.96456149
0.0%	minimum	-2.96456149

Summary Statistics

Mean	-0.6537
Std Dev	1.2095582
Std Err Mean	0.0084194
Upper 95% Mean	-0.637197
Lower 95% Mean	-0.670203
N	20639
Skewness	-0.264883
Kurtosis	-0.543034
N Missing	0
N Zero	0
Interquartile Range	1.6495142
Proportion Zero	0

(imputed) TCEM_I wt

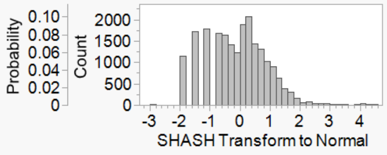

Quantiles

100.0%	maximum	4.4007077149
99.5%		2.4322684806
97.5%		1.672220991
90.0%		1.1003213825
75.0%	quartile	0.5909916126
50.0%	median	-0.112959283
25.0%	quartile	-0.705338025
10.0%		-1.417441656
2.5%		-1.992546039
0.5%		-1.992546039
0.0%	minimum	-2.96456149

Summary Statistics

Mean	-0.093191
Std Dev	0.9528666
Std Err Mean	0.0066327
Upper 95% Mean	-0.08019
Lower 95% Mean	-0.106191
N	20639
Skewness	0.0590781
Kurtosis	0.0226174
N Missing	0
N Zero	0
Interquartile Range	1.2963296
Proportion Zero	0

P5

(imputed) TCEM_I mut

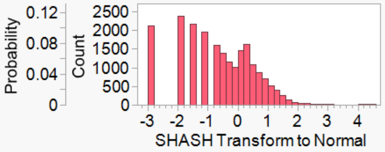

Quantiles		
100.0%	maximum	4.4007077149
99.5%		1.9246250168
97.5%		1.3964768526
90.0%		0.7885646032
75.0%	quartile	0.2320725377
50.0%	median	-0.46473686
25.0%	quartile	-1.417441656
10.0%		-2.96456149
2.5%		-2.96456149
0.5%		-2.96456149
0.0%	minimum	-2.96456149

Summary Statistics	
Mean	-0.66876
Std Dev	1.2108792
Std Err Mean	0.0084303
Upper 95% Mean	-0.652236
Lower 95% Mean	-0.685284
N	20631
Skewness	-0.255024
Kurtosis	-0.533064
N Missing	0
N Zero	0
Interquartile Range	1.6495142
Proportion Zero	0

(imputed) TCEM_I wt

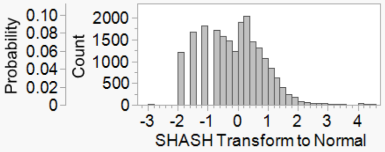

Quantiles		
100.0%	maximum	4.4140226357
99.5%		2.4218442787
97.5%		1.6593954702
90.0%		1.0716014667
75.0%	quartile	0.5319239347
50.0%	median	-0.112959283
25.0%	quartile	-0.705338025
10.0%		-1.417441656
2.5%		-1.992546039
0.5%		-1.992546039
0.0%	minimum	-2.96456149

Summary Statistics	
Mean	-0.105391
Std Dev	0.9501682
Std Err Mean	0.0066152
Upper 95% Mean	-0.092425
Lower 95% Mean	-0.118358
N	20631
Skewness	0.0513706
Kurtosis	0.0637122
N Missing	0
N Zero	0
Interquartile Range	1.237262
Proportion Zero	0

P6

(imputed) TCEM_I mut

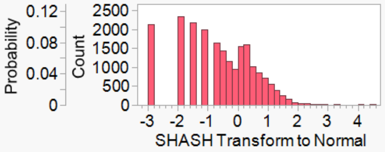

Quantiles		
100.0%	maximum	4.4007077149
99.5%		1.8411488566
97.5%		1.3775482226
90.0%		0.8305853509
75.0%	quartile	0.2320725377
50.0%	median	-0.46473686
25.0%	quartile	-1.417441656
10.0%		-2.96456149
2.5%		-2.96456149
0.5%		-2.96456149
0.0%	minimum	-2.96456149

Summary Statistics	
Mean	-0.673325
Std Dev	1.2075933
Std Err Mean	0.0084062
Upper 95% Mean	-0.656848
Lower 95% Mean	-0.689801
N	20637
Skewness	-0.266649
Kurtosis	-0.553346
N Missing	0
N Zero	0
Interquartile Range	1.6495142
Proportion Zero	0

(imputed) TCEM_I wt

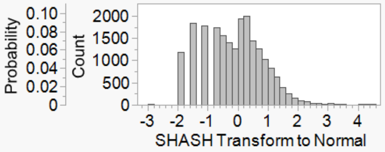

Quantiles		
100.0%	maximum	4.4140226357
99.5%		2.3518396686
97.5%		1.672220991
90.0%		1.0716014667
75.0%	quartile	0.5319239347
50.0%	median	-0.112959283
25.0%	quartile	-0.705338025
10.0%		-1.417441656
2.5%		-1.992546039
0.5%		-1.992546039
0.0%	minimum	-2.96456149

Summary Statistics	
Mean	-0.11533
Std Dev	0.9554165
Std Err Mean	0.0066507
Upper 95% Mean	-0.102294
Lower 95% Mean	-0.128366
N	20637
Skewness	0.0639598
Kurtosis	-0.010663
N Missing	0
N Zero	0
Interquartile Range	1.237262
Proportion Zero	0

P7

(imputed) TCEM_I mut

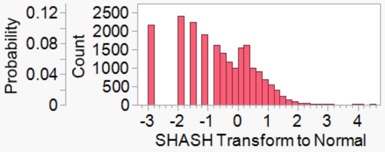

Quantiles		
100.0%	maximum	4.4007077149
99.5%		1.9326383839
97.5%		1.3775482226
90.0%		0.7885646032
75.0%	quartile	0.2320725377
50.0%	median	-0.46473686
25.0%	quartile	-1.417441656
10.0%		-2.96456149
2.5%		-2.96456149
0.5%		-2.96456149
0.0%	minimum	-2.96456149

Summary Statistics	
Mean	-0.677024
Std Dev	1.2126927
Std Err Mean	0.008439
Upper 95% Mean	-0.660483
Lower 95% Mean	-0.693566
N	20650
Skewness	-0.255136
Kurtosis	-0.569788
N Missing	0
N Zero	0
Interquartile Range	1.6495142
Proportion Zero	0

(imputed) TCEM_I wt

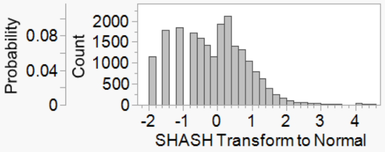

Quantiles		
100.0%	maximum	4.4007077149
99.5%		2.5005221441
97.5%		1.6971198823
90.0%		1.0716014667
75.0%	quartile	0.5319239347
50.0%	median	-0.112959283
25.0%	quartile	-0.705338025
10.0%		-1.417441656
2.5%		-1.992546039
0.5%		-1.992546039
0.0%	minimum	-1.992546039

Summary Statistics	
Mean	-0.102257
Std Dev	0.9571022
Std Err Mean	0.0066604
Upper 95% Mean	-0.089202
Lower 95% Mean	-0.115311
N	20650
Skewness	0.1091572
Kurtosis	0.1239103
N Missing	0
N Zero	0
Interquartile Range	1.237262
Proportion Zero	0

P8

(imputed) TCEM_I mut

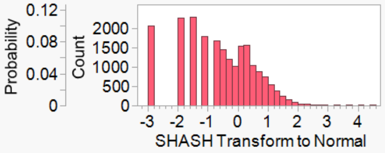

Quantiles		
100.0%	maximum	4.4007077149
99.5%		1.8988699401
97.5%		1.3964768526
90.0%		0.8305853509
75.0%	quartile	0.2320725377
50.0%	median	-0.46473686
25.0%	quartile	-1.417441656
10.0%		-1.992546039
2.5%		-2.96456149
0.5%		-2.96456149
0.0%	minimum	-2.96456149

Summary Statistics	
Mean	-0.655268
Std Dev	1.2050571
Std Err Mean	0.0083848
Upper 95% Mean	-0.638833
Lower 95% Mean	-0.671703
N	20655
Skewness	-0.267835
Kurtosis	-0.509622
N Missing	0
N Zero	0
Interquartile Range	1.6495142
Proportion Zero	0

(imputed) TCEM_I wt

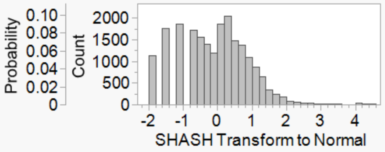

Quantiles		
100.0%	maximum	4.4007077149
99.5%		2.4130198666
97.5%		1.672220991
90.0%		1.0716014667
75.0%	quartile	0.5909916126
50.0%	median	-0.112959283
25.0%	quartile	-0.705338025
10.0%		-1.417441656
2.5%		-1.992546039
0.5%		-1.992546039
0.0%	minimum	-1.992546039

Summary Statistics	
Mean	-0.10023
Std Dev	0.9522232
Std Err Mean	0.0066256
Upper 95% Mean	-0.087243
Lower 95% Mean	-0.113216
N	20655
Skewness	0.0836119
Kurtosis	0.1388087
N Missing	0
N Zero	0
Interquartile Range	1.2963296
Proportion Zero	0

P9

(imputed) TCEM_I mut

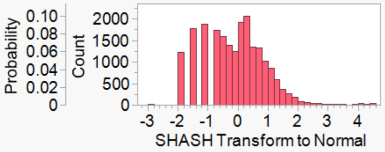

Quantiles		
100.0%	maximum	4.4140226357
99.5%		2.3765592684
97.5%		1.672220991
90.0%		1.0716014667
75.0%	quartile	0.5319239347
50.0%	median	-0.112959283
25.0%	quartile	-0.705338025
10.0%		-1.417441656
2.5%		-1.992546039
0.5%		-1.992546039
0.0%	minimum	-2.96456149

Summary Statistics	
Mean	-0.121349
Std Dev	0.9545669
Std Err Mean	0.0066419
Upper 95% Mean	-0.108331
Lower 95% Mean	-0.134368
N	20655
Skewness	0.0851911
Kurtosis	0.0782634
N Missing	0
N Zero	0
Interquartile Range	1.237262
Proportion Zero	0

(imputed) TCEM_I wt

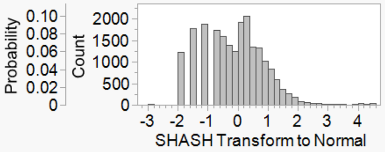

Quantiles		
100.0%	maximum	4.4140226357
99.5%		2.3799168661
97.5%		1.672220991
90.0%		1.0716014667
75.0%	quartile	0.5319239347
50.0%	median	-0.112959283
25.0%	quartile	-0.705338025
10.0%		-1.417441656
2.5%		-1.992546039
0.5%		-1.992546039
0.0%	minimum	-2.96456149

Summary Statistics	
Mean	-0.12101
Std Dev	0.9553742
Std Err Mean	0.0066475
Upper 95% Mean	-0.107981
Lower 95% Mean	-0.13404
N	20655
Skewness	0.0917746
Kurtosis	0.1043271
N Missing	0
N Zero	0
Interquartile Range	1.237262
Proportion Zero	0

MHC II   Mutant blue; WT grey

P1

(imputed) TCEM_IIa hg38 mut

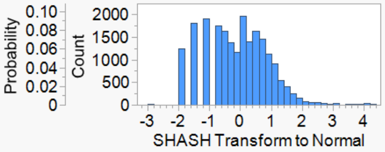

Quantiles		
100.0%	maximum	4.3576442381
99.5%		2.3536696615
97.5%		1.6413656645
90.0%		1.0820989486
75.0%	quartile	0.5556960118
50.0%	median	-0.0996766
25.0%	quartile	-0.694978493
10.0%		-1.412437531
2.5%		-1.995833784
0.5%		-1.995833784
0.0%	minimum	-2.988925681

Summary Statistics	
Mean	-0.123275
Std Dev	0.9578271
Std Err Mean	0.0066672
Upper 95% Mean	-0.110206
Lower 95% Mean	-0.136343
N	20639
Skewness	0.0393439
Kurtosis	-0.074526
N Missing	0
N Zero	0
Interquartile Range	1.2506745
Proportion Zero	0

(imputed) TCEM_IIa hg38 wt

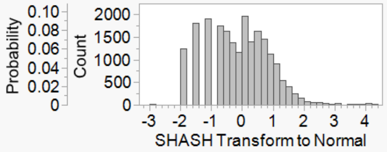

Quantiles		
100.0%	maximum	4.3576442381
99.5%		2.3536696615
97.5%		1.6413656645
90.0%		1.0820989486
75.0%	quartile	0.5556960118
50.0%	median	-0.0996766
25.0%	quartile	-0.694978493
10.0%		-1.412437531
2.5%		-1.995833784
0.5%		-1.995833784
0.0%	minimum	-2.988925681

Summary Statistics	
Mean	-0.123275
Std Dev	0.9578271
Std Err Mean	0.0066672
Upper 95% Mean	-0.110206
Lower 95% Mean	-0.136343
N	20639
Skewness	0.0393439
Kurtosis	-0.074526
N Missing	0
N Zero	0
Interquartile Range	1.2506745
Proportion Zero	0

P2

(imputed) TCEM_IIa hg38 mut

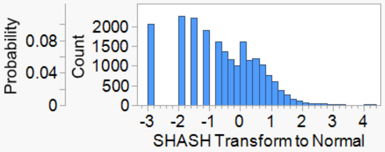

Quantiles		
100.0%	maximum	4.3576442381
99.5%		2.006804034
97.5%		1.4322329509
90.0%		0.8198564948
75.0%	quartile	0.2495025872
50.0%	median	-0.453398355
25.0%	quartile	-1.412437531
10.0%		-1.995833784
2.5%		-2.988925681
0.5%		-2.988925681
0.0%	minimum	-2.988925681

Summary Statistics	
Mean	-0.641388
Std Dev	1.2209941
Std Err Mean	0.0085007
Upper 95% Mean	-0.624726
Lower 95% Mean	-0.65805
N	20631
Skewness	-0.270708
Kurtosis	-0.489497
N Missing	0
N Zero	0
Interquartile Range	1.6619401
Proportion Zero	0

(imputed) TCEM_IIa hg38 wt

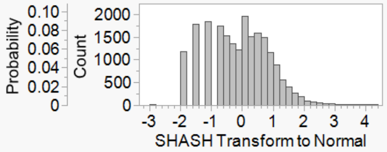

Quantiles		
100.0%	maximum	4.3576442381
99.5%		2.4481300047
97.5%		1.7139515443
90.0%		1.0820989486
75.0%	quartile	0.5556960118
50.0%	median	-0.0996766
25.0%	quartile	-0.694978493
10.0%		-1.412437531
2.5%		-1.995833784
0.5%		-1.995833784
0.0%	minimum	-2.988925681

Summary Statistics	
Mean	-0.096033
Std Dev	0.961798
Std Err Mean	0.0066961
Upper 95% Mean	-0.082908
Lower 95% Mean	-0.109157
N	20631
Skewness	0.0431305
Kurtosis	-0.076563
N Missing	0
N Zero	0
Interquartile Range	1.2506745
Proportion Zero	0

P3

(imputed) TCEM_IIa hg38 mut

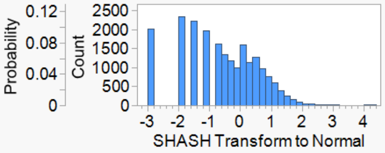

Quantiles		
100.0%	maximum	4.3576442381
99.5%		1.8864946714
97.5%		1.3910216969
90.0%		0.8198564948
75.0%	quartile	0.2495025872
50.0%	median	-0.453398355
25.0%	quartile	-1.412437531
10.0%		-1.995833784
2.5%		-2.988925681
0.5%		-2.988925681
0.0%	minimum	-2.988925681

Summary Statistics	
Mean	-0.650652
Std Dev	1.2130132
Std Err Mean	0.0084439
Upper 95% Mean	-0.634101
Lower 95% Mean	-0.667202
N	20637
Skewness	-0.277162
Kurtosis	-0.523123
N Missing	0
N Zero	0
Interquartile Range	1.6619401
Proportion Zero	0

(imputed) TCEM_IIa hg38 wt

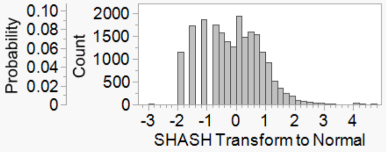

Quantiles		
100.0%	maximum	4.6387153143
99.5%		2.3780913103
97.5%		1.6999798949
90.0%		1.0820989486
75.0%	quartile	0.5556960118
50.0%	median	-0.0996766
25.0%	quartile	-0.694978493
10.0%		-1.412437531
2.5%		-1.995833784
0.5%		-1.995833784
0.0%	minimum	-2.988925681

Summary Statistics	
Mean	-0.098345
Std Dev	0.9508063
Std Err Mean	0.0066186
Upper 95% Mean	-0.085372
Lower 95% Mean	-0.111318
N	20637
Skewness	0.0279811
Kurtosis	-0.067881
N Missing	0
N Zero	0
Interquartile Range	1.2506745
Proportion Zero	0

P4

(imputed) TCEM_IIa hg38 mut

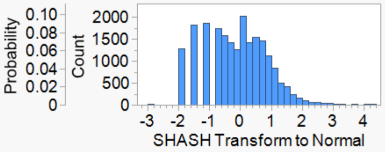

Quantiles		
100.0%	maximum	4.3576442381
99.5%		2.339876211
97.5%		1.6564494752
90.0%		1.0497016293
75.0%	quartile	0.5556960118
50.0%	median	-0.0996766
25.0%	quartile	-0.694978493
10.0%		-1.412437531
2.5%		-1.995833784
0.5%		-1.995833784
0.0%	minimum	-2.988925681

Summary Statistics	
Mean	-0.131043
Std Dev	0.9533272
Std Err Mean	0.0066341
Upper 95% Mean	-0.118039
Lower 95% Mean	-0.144046
N	20650
Skewness	0.0250298
Kurtosis	-0.157775
N Missing	0
N Zero	0
Interquartile Range	1.2506745
Proportion Zero	0

(imputed) TCEM_IIa hg38 wt

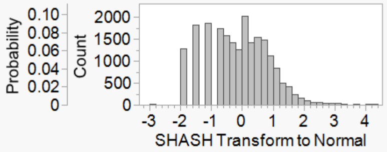

Quantiles		
100.0%	maximum	4.3576442381
99.5%		2.3665634264
97.5%		1.6564494752
90.0%		1.0497016293
75.0%	quartile	0.5556960118
50.0%	median	-0.0996766
25.0%	quartile	-0.694978493
10.0%		-1.412437531
2.5%		-1.995833784
0.5%		-1.995833784
0.0%	minimum	-2.988925681

Summary Statistics	
Mean	-0.130792
Std Dev	0.9538549
Std Err Mean	0.0066378
Upper 95% Mean	-0.117781
Lower 95% Mean	-0.143802
N	20650
Skewness	0.0282554
Kurtosis	-0.149973
N Missing	0
N Zero	0
Interquartile Range	1.2506745
Proportion Zero	0

P5

(imputed) TCEM_IIa hg38 mut

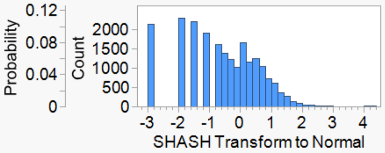

Quantiles		
100.0%	maximum	4.3576442381
99.5%		1.8864946714
97.5%		1.403552177
90.0%		0.8198564948
75.0%	quartile	0.2495025872
50.0%	median	-0.453398355
25.0%	quartile	-1.412437531
10.0%		-2.988925681
2.5%		-2.988925681
0.5%		-2.988925681
0.0%	minimum	-2.988925681

Summary Statistics	
Mean	-0.652272
Std Dev	1.2173177
Std Err Mean	0.0084702
Upper 95% Mean	-0.63567
Lower 95% Mean	-0.668874
N	20655
Skewness	-0.30965
Kurtosis	-0.551678
N Missing	0
N Zero	0
Interquartile Range	1.6619401
Proportion Zero	0

(imputed) TCEM_IIa hg38 wt

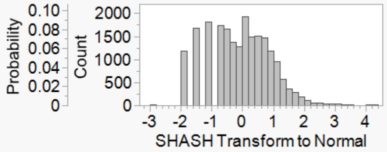

Quantiles		
100.0%	maximum	4.3576442381
99.5%		2.4116421066
97.5%		1.6999798949
90.0%		1.0820989486
75.0%	quartile	0.5556960118
50.0%	median	-0.0996766
25.0%	quartile	-0.694978493
10.0%		-1.412437531
2.5%		-1.995833784
0.5%		-1.995833784
0.0%	minimum	-2.988925681

Summary Statistics	
Mean	-0.092444
Std Dev	0.9559724
Std Err Mean	0.0066517
Upper 95% Mean	-0.079406
Lower 95% Mean	-0.105482
N	20655
Skewness	0.0275958
Kurtosis	-0.099683
N Missing	0
N Zero	0
Interquartile Range	1.2506745
Proportion Zero	0

P6

(imputed) TCEM_IIa hg38 mut

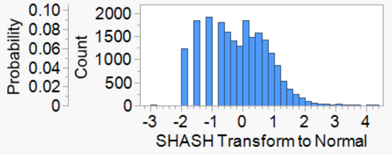

Quantiles

100.0%	maximum	4.3576442381
99.5%		2.3043182425
97.5%		1.6413656645
90.0%		1.0497016293
75.0%	quartile	0.5556960118
50.0%	median	-0.0996766
25.0%	quartile	-0.694978493
10.0%		-1.412437531
2.5%		-1.995833784
0.5%		-1.995833784
0.0%	minimum	-2.988925681

Summary Statistics

Mean	-0.134549
Std Dev	0.9500457
Std Err Mean	0.0066105
Upper 95% Mean	-0.121592
Lower 95% Mean	-0.147506
N	20655
Skewness	0.0262877
Kurtosis	-0.202597
N Missing	0
N Zero	0
Interquartile Range	1.2506745
Proportion Zero	0

(imputed) TCEM_IIa hg38 wt

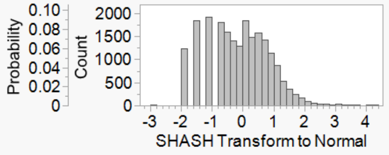

Quantiles

100.0%	maximum	4.3576442381
99.5%		2.3060894939
97.5%		1.6504159509
90.0%		1.0497016293
75.0%	quartile	0.5556960118
50.0%	median	-0.0996766
25.0%	quartile	-0.694978493
10.0%		-1.412437531
2.5%		-1.995833784
0.5%		-1.995833784
0.0%	minimum	-2.988925681

Summary Statistics

Mean	-0.134408
Std Dev	0.9504493
Std Err Mean	0.0066133
Upper 95% Mean	-0.121445
Lower 95% Mean	-0.14737
N	20655
Skewness	0.0297905
Kurtosis	-0.189543
N Missing	0
N Zero	0
Interquartile Range	1.2506745
Proportion Zero	0

P7

(imputed) TCEM_IIa hg38 mut

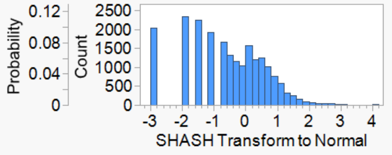

Quantiles

100.0%	maximum	4.1033956706
99.5%		1.9893300714
97.5%		1.4322329509
90.0%		0.8198564948
75.0%	quartile	0.2495025872
50.0%	median	-0.453398355
25.0%	quartile	-1.412437531
10.0%		-1.995833784
2.5%		-2.988925681
0.5%		-2.988925681
0.0%	minimum	-2.988925681

Summary Statistics

Mean	-0.648577
Std Dev	1.2137607
Std Err Mean	0.0084438
Upper 95% Mean	-0.632026
Lower 95% Mean	-0.665127
N	20663
Skewness	-0.272376
Kurtosis	-0.513277
N Missing	0
N Zero	0
Interquartile Range	1.6619401
Proportion Zero	0

(imputed) TCEM_IIa hg38 wt

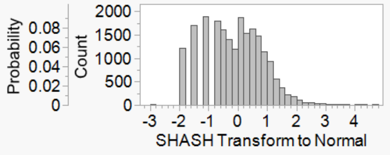

Quantiles

100.0%	maximum	4.6387153143
99.5%		2.4244185133
97.5%		1.6999798949
90.0%		1.0820989486
75.0%	quartile	0.5556960118
50.0%	median	-0.0996766
25.0%	quartile	-0.694978493
10.0%		-1.412437531
2.5%		-1.995833784
0.5%		-1.995833784
0.0%	minimum	-2.988925681

Summary Statistics

Mean	-0.106418
Std Dev	0.9546964
Std Err Mean	0.0066415
Upper 95% Mean	-0.0934
Lower 95% Mean	-0.119435
N	20663
Skewness	0.0469105
Kurtosis	-0.012841
N Missing	0
N Zero	0
Interquartile Range	1.2506745
Proportion Zero	0

P8

(imputed) TCEM_IIa hg38 mut

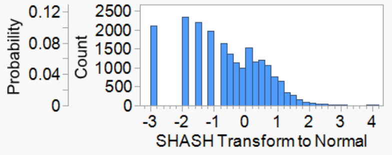

Quantiles

100.0%	maximum	4.0365664637
99.5%		2.0050120237
97.5%		1.4520318097
90.0%		0.8632795849
75.0%	quartile	0.2495025872
50.0%	median	-0.453398355
25.0%	quartile	-1.412437531
10.0%		-2.988925681
2.5%		-2.988925681
0.5%		-2.988925681
0.0%	minimum	-2.988925681

Summary Statistics

Mean	-0.649531
Std Dev	1.2261006
Std Err Mean	0.0085284
Upper 95% Mean	-0.632815
Lower 95% Mean	-0.666248
N	20669
Skewness	-0.269625
Kurtosis	-0.558562
N Missing	0
N Zero	0
Interquartile Range	1.6619401
Proportion Zero	0

(imputed) TCEM_IIa hg38 wt

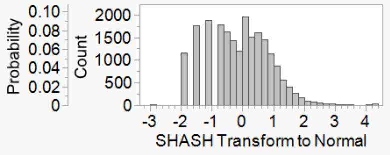

Quantiles

100.0%	maximum	4.3576442381
99.5%		2.4170898769
97.5%		1.6857457387
90.0%		1.0820989486
75.0%	quartile	0.5556960118
50.0%	median	-0.0996766
25.0%	quartile	-0.694978493
10.0%		-1.412437531
2.5%		-1.995833784
0.5%		-1.995833784
0.0%	minimum	-2.988925681

Summary Statistics

Mean	-0.102234
Std Dev	0.9536849
Std Err Mean	0.0066335
Upper 95% Mean	-0.089231
Lower 95% Mean	-0.115236
N	20669
Skewness	0.065334
Kurtosis	-0.03814
N Missing	0
N Zero	0
Interquartile Range	1.2506745
Proportion Zero	0

P9

(imputed) TCEM_IIa hg38 mut

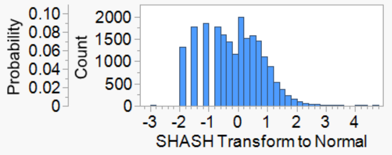

Quantiles

100.0%	maximum	4.6387153143
99.5%		2.324670092
97.5%		1.6413656645
90.0%		1.0497016293
75.0%	quartile	0.5556960118
50.0%	median	-0.0996766
25.0%	quartile	-0.694978493
10.0%		-1.412437531
2.5%		-1.995833784
0.5%		-1.995833784
0.0%	minimum	-2.988925681

Summary Statistics

Mean	-0.132824
Std Dev	0.9550085
Std Err Mean	0.0066432
Upper 95% Mean	-0.119803
Lower 95% Mean	-0.145845
N	20666
Skewness	0.017333
Kurtosis	-0.084482
N Missing	0
N Zero	0
Interquartile Range	1.2506745
Proportion Zero	0

(imputed) TCEM_IIa hg38 wt

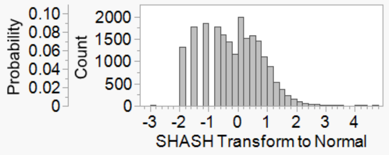

Quantiles

100.0%	maximum	4.6387153143
99.5%		2.3308702857
97.5%		1.6413656645
90.0%		1.0497016293
75.0%	quartile	0.5556960118
50.0%	median	-0.0996766
25.0%	quartile	-0.694978493
10.0%		-1.412437531
2.5%		-1.995833784
0.5%		-1.995833784
0.0%	minimum	-2.988925681

Summary Statistics

Mean	-0.13253
Std Dev	0.955442
Std Err Mean	0.0066462
Upper 95% Mean	-0.119503
Lower 95% Mean	-0.145557
N	20666
Skewness	0.020622
Kurtosis	-0.072395
N Missing	0
N Zero	0
Interquartile Range	1.2506745
Proportion Zero	0

Oncogenes and Suppressors Pocket specific changes – MHC I and MHC II Normalized log distributions relative to GI Microbiome

MHC I   Mutant red; WT grey

P1

Normalized log2(N TCEM_I GI microbiome) mut

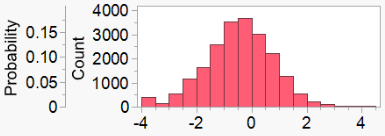

Quantiles

100.0%	maximum	4.4293941696
99.5%		2.4076590242
97.5%		1.6861626254
90.0%		0.9802019413
75.0%	quartile	0.3151547498
50.0%	median	-0.430530809
25.0%	quartile	-1.209043765
10.0%		-2.017890679
2.5%		-2.915750967
0.5%		-3.893218834
0.0%	minimum	-3.893218834

Summary Statistics

Mean	-0.486653
Std Dev	1.1965008
Std Err Mean	0.0083308
Upper 95% Mean	-0.470324
Lower 95% Mean	-0.502982
N	20628
Skewness	-0.325342
Kurtosis	0.3235113
N Missing	0
N Zero	0
Interquartile Range	1.5241985
Proportion Zero	0

Normalized log2(N TCEM_I GI microbiome) wt

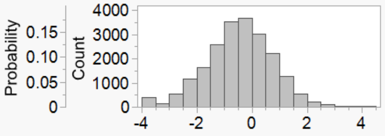

Quantiles

100.0%	maximum	4.4293941696
99.5%		2.4076590242
97.5%		1.6861626254
90.0%		0.9802019413
75.0%	quartile	0.3151547498
50.0%	median	-0.430530809
25.0%	quartile	-1.209043765
10.0%		-2.017890679
2.5%		-2.915750967
0.5%		-3.893218834
0.0%	minimum	-3.893218834

Summary Statistics

Mean	-0.486653
Std Dev	1.1965008
Std Err Mean	0.0083308
Upper 95% Mean	-0.470324
Lower 95% Mean	-0.502982
N	20628
Skewness	-0.325342
Kurtosis	0.3235113
N Missing	0
N Zero	0
Interquartile Range	1.5241985
Proportion Zero	0

P2

Normalized log2(N TCEM_I GI microbiome) mut

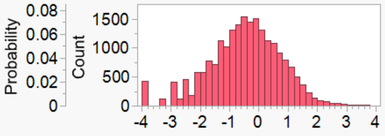

Quantiles

100.0%	maximum	3.7653212978
99.5%		2.3318452103
97.5%		1.6288870015
90.0%		0.9933675483
75.0%	quartile	0.3264699299
50.0%	median	-0.430530809
25.0%	quartile	-1.209043765
10.0%		-2.017890679
2.5%		-2.915750967
0.5%		-3.893218834
0.0%	minimum	-3.893218834

Summary Statistics

Mean	-0.492548
Std Dev	1.2011113
Std Err Mean	0.00836
Upper 95% Mean	-0.476162
Lower 95% Mean	-0.508935
N	20642
Skewness	-0.363997
Kurtosis	0.3137832
N Missing	0
N Zero	0
Interquartile Range	1.5355137
Proportion Zero	0

Normalized log2(N TCEM_I GI microbiome) wt

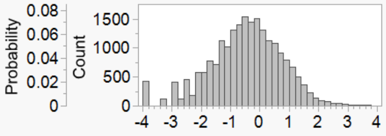

Quantiles

100.0%	maximum	3.7653212978
99.5%		2.3318452103
97.5%		1.6288870015
90.0%		0.9933675483
75.0%	quartile	0.3264699299
50.0%	median	-0.430530809
25.0%	quartile	-1.209043765
10.0%		-2.017890679
2.5%		-2.915750967
0.5%		-3.893218834
0.0%	minimum	-3.893218834

Summary Statistics

Mean	-0.492548
Std Dev	1.2011113
Std Err Mean	0.00836
Upper 95% Mean	-0.476162
Lower 95% Mean	-0.508935
N	20642
Skewness	-0.363997
Kurtosis	0.3137832
N Missing	0
N Zero	0
Interquartile Range	1.5355137
Proportion Zero	0

P3

Normalized log2(N TCEM_I GI microbiome) mut

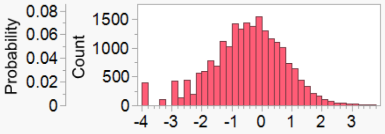

Quantiles

100.0%	maximum	3.7163436348
99.5%		2.3674391809
97.5%		1.6685899773
90.0%		0.9735617842
75.0%	quartile	0.3376915614
50.0%	median	-0.430530809
25.0%	quartile	-1.209043765
10.0%		-2.017890679
2.5%		-2.915750967
0.5%		-3.893218834
0.0%	minimum	-3.893218834

Summary Statistics

Mean	-0.489927
Std Dev	1.1975511
Std Err Mean	0.0083352
Upper 95% Mean	-0.473589
Lower 95% Mean	-0.506265
N	20642
Skewness	-0.344345
Kurtosis	0.287479
N Missing	0
N Zero	0
Interquartile Range	1.5467353
Proportion Zero	0

Normalized log2(N TCEM_I GI microbiome) wt

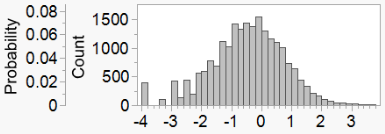

Quantiles

100.0%	maximum	3.7163436348
99.5%		2.3674391809
97.5%		1.6685899773
90.0%		0.9735617842
75.0%	quartile	0.3376915614
50.0%	median	-0.430530809
25.0%	quartile	-1.209043765
10.0%		-2.017890679
2.5%		-2.915750967
0.5%		-3.893218834
0.0%	minimum	-3.893218834

Summary Statistics

Mean	-0.489927
Std Dev	1.1975511
Std Err Mean	0.0083352
Upper 95% Mean	-0.473589
Lower 95% Mean	-0.506265
N	20642
Skewness	-0.344345
Kurtosis	0.287479
N Missing	0
N Zero	0
Interquartile Range	1.5467353
Proportion Zero	0

P4

Normalized log2(N TCEM_I GI microbiome) mut

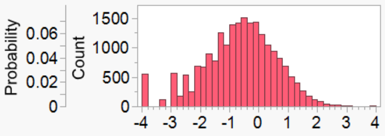

Quantiles

100.0%	maximum	3.9418056779
99.5%		2.0855241559
97.5%		1.5166092563
90.0%		0.8320264878
75.0%	quartile	0.1716058215
50.0%	median	-0.574206096
25.0%	quartile	-1.387398895
10.0%		-2.264499765
2.5%		-3.893218834
0.5%		-3.893218834
0.0%	minimum	-3.893218834

Summary Statistics

Mean	-0.65814
Std Dev	1.2117948
Std Err Mean	0.008435
Upper 95% Mean	-0.641607
Lower 95% Mean	-0.674674
N	20639
Skewness	-0.360255
Kurtosis	0.152431
N Missing	0
N Zero	0
Interquartile Range	1.5590047
Proportion Zero	0

Normalized log2(N TCEM_I GI microbiome) wt

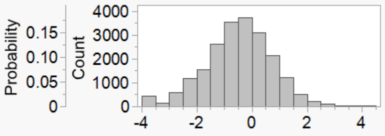

Quantiles

100.0%	maximum	4.4293941696
99.5%		2.414734022
97.5%		1.6931118503
90.0%		0.9735617842
75.0%	quartile	0.303744805
50.0%	median	-0.430530809
25.0%	quartile	-1.209043765
10.0%		-2.017890679
2.5%		-2.915750967
0.5%		-3.893218834
0.0%	minimum	-3.893218834

Summary Statistics

Mean	-0.492938
Std Dev	1.1973306
Std Err Mean	0.0083343
Upper 95% Mean	-0.476602
Lower 95% Mean	-0.509274
N	20639
Skewness	-0.336655
Kurtosis	0.3555827
N Missing	0
N Zero	0
Interquartile Range	1.5127886
Proportion Zero	0

P5

Normalized log2(N TCEM_I GI microbiome) mut

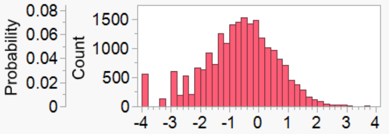

Quantiles		
100.0%	maximum	3.7653212978
99.5%		2.1115271571
97.5%		1.5206815509
90.0%		0.8320264878
75.0%	quartile	0.1589534027
50.0%	median	-0.596203998
25.0%	quartile	-1.387398895
10.0%		-2.264499765
2.5%		-3.893218834
0.5%		-3.893218834
0.0%	minimum	-3.893218834

Summary Statistics	
Mean	-0.662266
Std Dev	1.2143681
Std Err Mean	0.0084545
Upper 95% Mean	-0.645694
Lower 95% Mean	-0.678838
N	20631
Skewness	-0.370355
Kurtosis	0.1869148
N Missing	0
N Zero	0
Interquartile Range	1.5463523
Proportion Zero	0

Normalized log2(N TCEM_I GI microbiome) wt

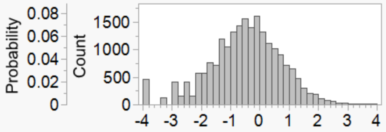

Quantiles		
100.0%	maximum	3.9518635913
99.5%		2.3441066616
97.5%		1.6728328089
90.0%		0.9802019413
75.0%	quartile	0.2922388553
50.0%	median	-0.450061004
25.0%	quartile	-1.209043765
10.0%		-2.017890679
2.5%		-3.393218834
0.5%		-3.893218834
0.0%	minimum	-3.893218834

Summary Statistics	
Mean	-0.508282
Std Dev	1.2018994
Std Err Mean	0.0083677
Upper 95% Mean	-0.49188
Lower 95% Mean	-0.524683
N	20631
Skewness	-0.373341
Kurtosis	0.3700277
N Missing	0
N Zero	0
Interquartile Range	1.5012826
Proportion Zero	0

P6

Normalized log2(N TCEM_I GI microbiome) mut

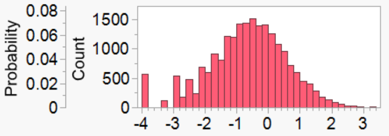

Quantiles		
100.0%	maximum	3.2061583817
99.5%		2.123137516
97.5%		1.5289818157
90.0%		0.8168776737
75.0%	quartile	0.1461875659
50.0%	median	-0.596203998
25.0%	quartile	-1.387398895
10.0%		-2.131459517
2.5%		-3.893218834
0.5%		-3.893218834
0.0%	minimum	-3.893218834

Summary Statistics	
Mean	-0.662481
Std Dev	1.2069128
Std Err Mean	0.0084014
Upper 95% Mean	-0.646014
Lower 95% Mean	-0.678948
N	20637
Skewness	-0.365304
Kurtosis	0.2307759
N Missing	0
N Zero	0
Interquartile Range	1.5335865
Proportion Zero	0

Normalized log2(N TCEM_I GI microbiome) wt

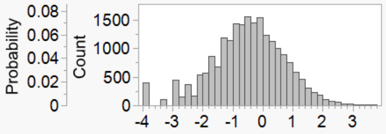

Quantiles		
100.0%	maximum	3.7555283176
99.5%		2.254156194
97.5%		1.6896428684
90.0%		0.9534069756
75.0%	quartile	0.2806356349
50.0%	median	-0.469899304
25.0%	quartile	-1.209043765
10.0%		-2.017890679
2.5%		-2.915750967
0.5%		-3.893218834
0.0%	minimum	-3.893218834

Summary Statistics	
Mean	-0.510704
Std Dev	1.1804941
Std Err Mean	0.0082175
Upper 95% Mean	-0.494597
Lower 95% Mean	-0.526811
N	20637
Skewness	-0.32903
Kurtosis	0.3257235
N Missing	0
N Zero	0
Interquartile Range	1.4896794
Proportion Zero	0

P7

Normalized log2(N TCEM_I GI microbiome) mut

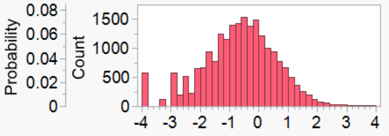

Quantiles		
100.0%	maximum	3.9518635913
99.5%		2.1515901139
97.5%		1.5487664441
90.0%		0.8320264878
75.0%	quartile	0.1461875659
50.0%	median	-0.596203998
25.0%	quartile	-1.387398895
10.0%		-2.264499765
2.5%		-3.893218834
0.5%		-3.893218834
0.0%	minimum	-3.893218834

Summary Statistics	
Mean	-0.666469
Std Dev	1.2171186
Std Err Mean	0.0084698
Upper 95% Mean	-0.649867
Lower 95% Mean	-0.68307
N	20650
Skewness	-0.342126
Kurtosis	0.2064974
N Missing	0
N Zero	0
Interquartile Range	1.5335865
Proportion Zero	0

Normalized log2(N TCEM_I GI microbiome) wt

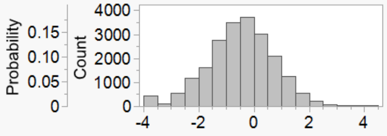

Quantiles		
100.0%	maximum	4.4293941696
99.5%		2.2853745
97.5%		1.6614795455
90.0%		0.9802019413
75.0%	quartile	0.303744805
50.0%	median	-0.450061004
25.0%	quartile	-1.209043765
10.0%		-2.017890679
2.5%		-2.915750967
0.5%		-3.893218834
0.0%	minimum	-3.893218834

Summary Statistics	
Mean	-0.503761
Std Dev	1.1933991
Std Err Mean	0.0083047
Upper 95% Mean	-0.487483
Lower 95% Mean	-0.520039
N	20650
Skewness	-0.339884
Kurtosis	0.3207438
N Missing	0
N Zero	0
Interquartile Range	1.5127886
Proportion Zero	0

P8

Normalized log2(N TCEM_I GI microbiome) mut

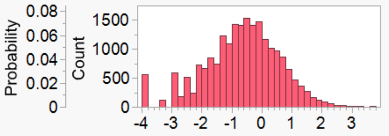

Quantiles		
100.0%	maximum	3.6684306776
99.5%		2.1310496193
97.5%		1.5125215917
90.0%		0.8168776737
75.0%	quartile	0.1589534027
50.0%	median	-0.596203998
25.0%	quartile	-1.387398895
10.0%		-2.264499765
2.5%		-3.893218834
0.5%		-3.893218834
0.0%	minimum	-3.893218834

Summary Statistics	
Mean	-0.663086
Std Dev	1.2132818
Std Err Mean	0.0084421
Upper 95% Mean	-0.646539
Lower 95% Mean	-0.679633
N	20655
Skewness	-0.360367
Kurtosis	0.1701761
N Missing	0
N Zero	0
Interquartile Range	1.5463523
Proportion Zero	0

Normalized log2(N TCEM_I GI microbiome) wt

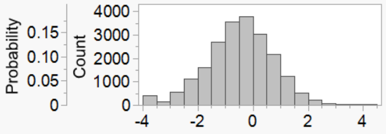

Quantiles		
100.0%	maximum	4.4293941696
99.5%		2.3256205574
97.5%		1.6434947348
90.0%		0.9534069756
75.0%	quartile	0.2922388553
50.0%	median	-0.430530809
25.0%	quartile	-1.209043765
10.0%		-2.017890679
2.5%		-2.915750967
0.5%		-3.893218834
0.0%	minimum	-3.893218834

Summary Statistics	
Mean	-0.496187
Std Dev	1.1796823
Std Err Mean	0.0082083
Upper 95% Mean	-0.480099
Lower 95% Mean	-0.512276
N	20655
Skewness	-0.35739
Kurtosis	0.3537173
N Missing	0
N Zero	0
Interquartile Range	1.5012826
Proportion Zero	0

P9

Normalized log2(N TCEM_I GI microbiome) mut

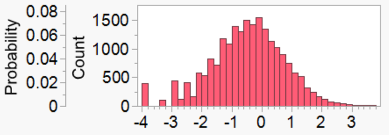

Quantiles		
100.0%	maximum	3.7163436348
99.5%		2.3569673465
97.5%		1.7170852611
90.0%		0.9894292563
75.0%	quartile	0.303744805
50.0%	median	-0.430530809
25.0%	quartile	-1.209043765
10.0%		-2.017890679
2.5%		-2.915750967
0.5%		-3.893218834
0.0%	minimum	-3.893218834

Summary Statistics	
Mean	-0.492461
Std Dev	1.1948036
Std Err Mean	0.0083135
Upper 95% Mean	-0.476165
Lower 95% Mean	-0.508756
N	20655
Skewness	-0.324278
Kurtosis	0.2905651
N Missing	0
N Zero	0
Interquartile Range	1.5127886
Proportion Zero	0

Normalized log2(N TCEM_I GI microbiome) wt

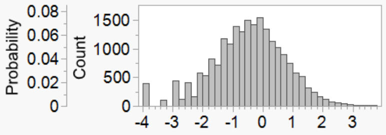

Quantiles		
100.0%	maximum	3.7163436348
99.5%		2.3569673465
97.5%		1.7204666416
90.0%		0.9933675483
75.0%	quartile	0.303744805
50.0%	median	-0.430530809
25.0%	quartile	-1.209043765
10.0%		-2.017890679
2.5%		-2.915750967
0.5%		-3.893218834
0.0%	minimum	-3.893218834

Summary Statistics	
Mean	-0.492278
Std Dev	1.1949758
Std Err Mean	0.0083147
Upper 95% Mean	-0.47598
Lower 95% Mean	-0.508575
N	20655
Skewness	-0.323904
Kurtosis	0.2903618
N Missing	0
N Zero	0
Interquartile Range	1.5127886
Proportion Zero	0

MHC II

P1

Normalized log2(N TCEM_IIa GI microbiome) mut

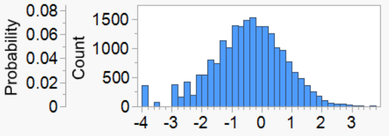

Quantiles

100.0%	maximum	3.7036430987
99.5%		2.2993847807
97.5%		1.6730074516
90.0%		0.9863793994
75.0%	quartile	0.3414759039
50.0%	median	-0.400967175
25.0%	quartile	-1.206801233
10.0%		-1.921186103
2.5%		-2.924411322
0.5%		-3.904789841
0.0%	minimum	-3.904789841

Summary Statistics

Mean	-0.465389
Std Dev	1.1792594
Std Err Mean	0.0082085
Upper 95% Mean	-0.449299
Lower 95% Mean	-0.481478
N	20639
Skewness	-0.336888
Kurtosis	0.3073659
N Missing	0
N Zero	0
Interquartile Range	1.5482771
Proportion Zero	0

Normalized log2(N TCEM_IIa GI microbiome) wt

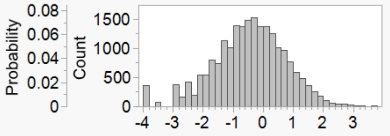

Quantiles

100.0%	maximum	3.7036430987
99.5%		2.2993847807
97.5%		1.6730074516
90.0%		0.9863793994
75.0%	quartile	0.3414759039
50.0%	median	-0.400967175
25.0%	quartile	-1.206801233
10.0%		-1.921186103
2.5%		-2.924411322
0.5%		-3.904789841
0.0%	minimum	-3.904789841

Summary Statistics

Mean	-0.465389
Std Dev	1.1792594
Std Err Mean	0.0082085
Upper 95% Mean	-0.449299
Lower 95% Mean	-0.481478
N	20639
Skewness	-0.336888
Kurtosis	0.3073659
N Missing	0
N Zero	0
Interquartile Range	1.5482771
Proportion Zero	0

P2

Normalized log2(N TCEM_IIa GI microbiome) mut

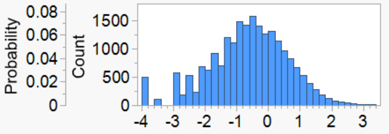

Quantiles

100.0%	maximum	3.3504103841
99.5%		2.2160480112
97.5%		1.5361679355
90.0%		0.8434073802
75.0%	quartile	0.1939026465
50.0%	median	-0.566045058
25.0%	quartile	-1.386448516
10.0%		-2.135333748
2.5%		-3.404789841
0.5%		-3.904789841
0.0%	minimum	-3.904789841

Summary Statistics

Mean	-0.633169
Std Dev	1.20738
Std Err Mean	0.0084059
Upper 95% Mean	-0.616692
Lower 95% Mean	-0.649645
N	20631
Skewness	-0.346004
Kurtosis	0.1704399
N Missing	0
N Zero	0
Interquartile Range	1.5803512
Proportion Zero	0

Normalized log2(N TCEM_IIa GI microbiome) wt

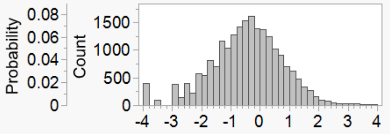

Quantiles

100.0%	maximum	3.8325435765
99.5%		2.3104731471
97.5%		1.6659576001
90.0%		0.979262728
75.0%	quartile	0.3178940981
50.0%	median	-0.420479033
25.0%	quartile	-1.206801233
10.0%		-2.02105992
2.5%		-2.924411322
0.5%		-3.904789841
0.0%	minimum	-3.904789841

Summary Statistics

Mean	-0.47943
Std Dev	1.1838559
Std Err Mean	0.0082421
Upper 95% Mean	-0.463275
Lower 95% Mean	-0.495585
N	20631
Skewness	-0.34292
Kurtosis	0.3775312
N Missing	0
N Zero	0
Interquartile Range	1.5246953
Proportion Zero	0

P3

Normalized log2(N TCEM_IIa GI microbiome) mut

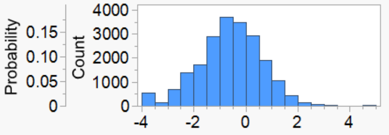

Quantiles

100.0%	maximum	4.6766784024
99.5%		2.1478196024
97.5%		1.5319707268
90.0%		0.8513667027
75.0%	quartile	0.1939026465
50.0%	median	-0.566045058
25.0%	quartile	-1.338078138
10.0%		-2.135333748
2.5%		-3.904789841
0.5%		-3.904789841
0.0%	minimum	-3.904789841

Summary Statistics

Mean	-0.633452
Std Dev	1.2054278
Std Err Mean	0.0083911
Upper 95% Mean	-0.617005
Lower 95% Mean	-0.649899
N	20637
Skewness	-0.373186
Kurtosis	0.2500524
N Missing	0
N Zero	0
Interquartile Range	1.5319808
Proportion Zero	0

Normalized log2(N TCEM_IIa GI microbiome) wt

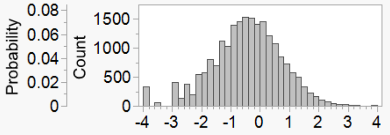

Quantiles

100.0%	maximum	3.8325435765
99.5%		2.2949753685
97.5%		1.6651727593
90.0%		0.9649011598
75.0%	quartile	0.3059568991
50.0%	median	-0.420479033
25.0%	quartile	-1.166894548
10.0%		-1.921186103
2.5%		-2.924411322
0.5%		-3.904789841
0.0%	minimum	-3.904789841

Summary Statistics

Mean	-0.4716
Std Dev	1.160618
Std Err Mean	0.0080792
Upper 95% Mean	-0.455764
Lower 95% Mean	-0.487436
N	20637
Skewness	-0.31905
Kurtosis	0.3495971
N Missing	0
N Zero	0
Interquartile Range	1.4728514
Proportion Zero	0

P4

Normalized log2(N TCEM_IIa GI microbiome) mut

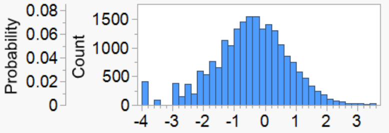

Quantiles

100.0%	maximum	3.4423431175
99.5%		2.4264281822
97.5%		1.7103388434
90.0%		0.9856677323
75.0%	quartile	0.3178940981
50.0%	median	-0.400967175
25.0%	quartile	-1.166894548
10.0%		-2.02105992
2.5%		-2.924411322
0.5%		-3.904789841
0.0%	minimum	-3.904789841

Summary Statistics

Mean	-0.472253
Std Dev	1.1924462
Std Err Mean	0.0082981
Upper 95% Mean	-0.455988
Lower 95% Mean	-0.488518
N	20650
Skewness	-0.334827
Kurtosis	0.4407796
N Missing	0
N Zero	0
Interquartile Range	1.4847886
Proportion Zero	0

Normalized log2(N TCEM_IIa GI microbiome) wt

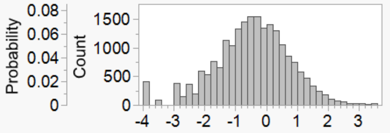

Quantiles

100.0%	maximum	3.4423431175
99.5%		2.4264281822
97.5%		1.7103388434
90.0%		0.9856677323
75.0%	quartile	0.3178940981
50.0%	median	-0.400967175
25.0%	quartile	-1.166894548
10.0%		-2.02105992
2.5%		-2.924411322
0.5%		-3.904789841
0.0%	minimum	-3.904789841

Summary Statistics

Mean	-0.472199
Std Dev	1.1922949
Std Err Mean	0.008297
Upper 95% Mean	-0.455936
Lower 95% Mean	-0.488462
N	20650
Skewness	-0.334719
Kurtosis	0.4416774
N Missing	0
N Zero	0
Interquartile Range	1.4847886
Proportion Zero	0

P5

Normalized log2(N TCEM_IIa GI microbiome) mut

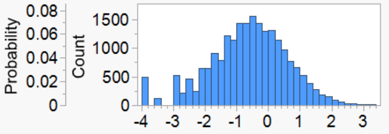

Quantiles

100.0%	maximum	3.2257288912
99.5%		2.1508394105
97.5%		1.5536692941
90.0%		0.8513667027
75.0%	quartile	0.180913487
50.0%	median	-0.566045058
25.0%	quartile	-1.386448516
10.0%		-2.135333748
2.5%		-3.404789841
0.5%		-3.904789841
0.0%	minimum	-3.904789841

Summary Statistics

Mean	-0.631816
Std Dev	1.2014731
Std Err Mean	0.0083599
Upper 95% Mean	-0.61543
Lower 95% Mean	-0.648202
N	20655
Skewness	-0.352916
Kurtosis	0.2051919
N Missing	0
N Zero	0
Interquartile Range	1.567362
Proportion Zero	0

Normalized log2(N TCEM_IIa GI microbiome) wt

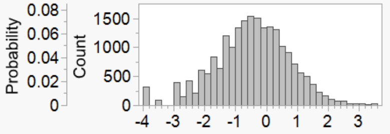

Quantiles

100.0%	maximum	3.4423431175
99.5%		2.4090878268
97.5%		1.7151433929
90.0%		0.9863793994
75.0%	quartile	0.3178940981
50.0%	median	-0.420479033
25.0%	quartile	-1.206801233
10.0%		-2.02105992
2.5%		-2.924411322
0.5%		-3.904789841
0.0%	minimum	-3.904789841

Summary Statistics

Mean	-0.472335
Std Dev	1.1824062
Std Err Mean	0.0082272
Upper 95% Mean	-0.456209
Lower 95% Mean	-0.488461
N	20655
Skewness	-0.271724
Kurtosis	0.3094483
N Missing	0
N Zero	0
Interquartile Range	1.5246953
Proportion Zero	0

P6

Normalized log2(N TCEM_IIa GI microbiome) mut

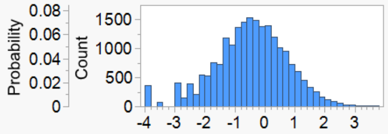

Quantiles

100.0%	maximum	3.7036430987
99.5%		2.323874095
97.5%		1.7000044791
90.0%		0.979262728
75.0%	quartile	0.3178940981
50.0%	median	-0.420479033
25.0%	quartile	-1.206801233
10.0%		-1.921186103
2.5%		-2.924411322
0.5%		-3.904789841
0.0%	minimum	-3.904789841

Summary Statistics

Mean	-0.475311
Std Dev	1.1787646
Std Err Mean	0.0082019
Upper 95% Mean	-0.459235
Lower 95% Mean	-0.491388
N	20655
Skewness	-0.321765
Kurtosis	0.3241452
N Missing	0
N Zero	0
Interquartile Range	1.5246953
Proportion Zero	0

Normalized log2(N TCEM_IIa GI microbiome) wt

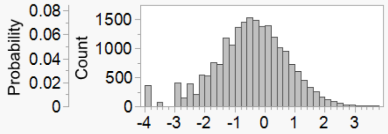

Quantiles

100.0%	maximum	3.7036430987
99.5%		2.323874095
97.5%		1.7000044791
90.0%		0.979262728
75.0%	quartile	0.3178940981
50.0%	median	-0.420479033
25.0%	quartile	-1.206801233
10.0%		-1.921186103
2.5%		-2.924411322
0.5%		-3.904789841
0.0%	minimum	-3.904789841

Summary Statistics

Mean	-0.475209
Std Dev	1.1788318
Std Err Mean	0.0082024
Upper 95% Mean	-0.459132
Lower 95% Mean	-0.491287
N	20655
Skewness	-0.321789
Kurtosis	0.3237806
N Missing	0
N Zero	0
Interquartile Range	1.5246953
Proportion Zero	0

P7

Normalized log2(N TCEM_IIa GI microbiome) mut

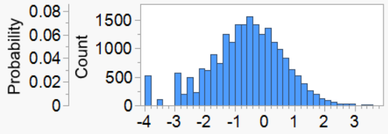

Quantiles

100.0%	maximum	3.5647005107
99.5%		2.1775702306
97.5%		1.5379282859
90.0%		0.8434073802
75.0%	quartile	0.1939026465
50.0%	median	-0.566045058
25.0%	quartile	-1.386448516
10.0%		-2.135333748
2.5%		-3.404789841
0.5%		-3.904789841
0.0%	minimum	-3.904789841

Summary Statistics

Mean	-0.632839
Std Dev	1.2095912
Std Err Mean	0.0084148
Upper 95% Mean	-0.616345
Lower 95% Mean	-0.649332
N	20663
Skewness	-0.366229
Kurtosis	0.2004704
N Missing	0
N Zero	0
Interquartile Range	1.5803512
Proportion Zero	0

Normalized log2(N TCEM_IIa GI microbiome) wt

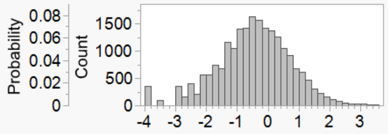

Quantiles

100.0%	maximum	3.4146619791
99.5%		2.3950046438
97.5%		1.6769043161
90.0%		0.9576553348
75.0%	quartile	0.3059568991
50.0%	median	-0.420479033
25.0%	quartile	-1.166894548
10.0%		-1.921186103
2.5%		-2.924411322
0.5%		-3.904789841
0.0%	minimum	-3.904789841

Summary Statistics

Mean	-0.477121
Std Dev	1.1666659
Std Err Mean	0.0081161
Upper 95% Mean	-0.461213
Lower 95% Mean	-0.49303
N	20663
Skewness	-0.323532
Kurtosis	0.4129639
N Missing	0
N Zero	0
Interquartile Range	1.4728514
Proportion Zero	0

P8

Normalized log2(N TCEM_IIa GI microbiome) mut

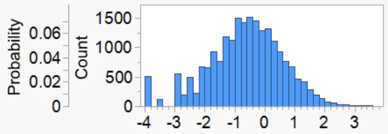

Quantiles

100.0%	maximum	3.5781219935
99.5%		2.1634354995
97.5%		1.5460442749
90.0%		0.8434073802
75.0%	quartile	0.1678110721
50.0%	median	-0.566045058
25.0%	quartile	-1.386448516
10.0%		-2.135333748
2.5%		-3.404789841
0.5%		-3.904789841
0.0%	minimum	-3.904789841

Summary Statistics

Mean	-0.640653
Std Dev	1.2055742
Std Err Mean	0.0083856
Upper 95% Mean	-0.624217
Lower 95% Mean	-0.65709
N	20669
Skewness	-0.341908
Kurtosis	0.1932679
N Missing	0
N Zero	0
Interquartile Range	1.5542596
Proportion Zero	0

Normalized log2(N TCEM_IIa GI microbiome) wt

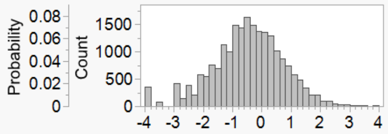

Quantiles

100.0%	maximum	3.8325435765
99.5%		2.3331295968
97.5%		1.7038084954
90.0%		0.9721034685
75.0%	quartile	0.3059568991
50.0%	median	-0.420479033
25.0%	quartile	-1.166894548
10.0%		-1.921186103
2.5%		-2.924411322
0.5%		-3.904789841
0.0%	minimum	-3.904789841

Summary Statistics

Mean	-0.475566
Std Dev	1.1747886
Std Err Mean	0.0081715
Upper 95% Mean	-0.45955
Lower 95% Mean	-0.491583
N	20669
Skewness	-0.317928
Kurtosis	0.3518748
N Missing	0
N Zero	0
Interquartile Range	1.4728514
Proportion Zero	0

P9

Normalized log2(N TCEM_IIa GI microbiome) mut

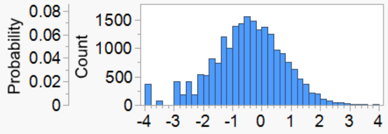

Quantiles

100.0%	maximum	3.8325435765
99.5%		2.328174633
97.5%		1.7000044791
90.0%		0.9934539371
75.0%	quartile	0.3059568991
50.0%	median	-0.440284685
25.0%	quartile	-1.206801233
10.0%		-2.02105992
2.5%		-2.924411322
0.5%		-3.904789841
0.0%	minimum	-3.904789841

Summary Statistics

Mean	-0.482665
Std Dev	1.1865479
Std Err Mean	0.0082539
Upper 95% Mean	-0.466487
Lower 95% Mean	-0.498843
N	20666
Skewness	-0.312386
Kurtosis	0.3050784
N Missing	0
N Zero	0
Interquartile Range	1.5127581
Proportion Zero	0

Normalized log2(N TCEM_IIa GI microbiome) wt

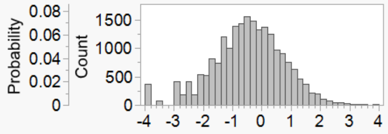

Quantiles

100.0%	maximum	3.8325435765
99.5%		2.328174633
97.5%		1.7000044791
90.0%		0.9934539371
75.0%	quartile	0.3059568991
50.0%	median	-0.440284685
25.0%	quartile	-1.206801233
10.0%		-2.02105992
2.5%		-2.924411322
0.5%		-3.904789841
0.0%	minimum	-3.904789841

Summary Statistics

Mean	-0.482595
Std Dev	1.1865889
Std Err Mean	0.0082541
Upper 95% Mean	-0.466417
Lower 95% Mean	-0.498774
N	20666
Skewness	-0.312366
Kurtosis	0.3049473
N Missing	0
N Zero	0
Interquartile Range	1.5127581
Proportion Zero	0

Supplemental Figure 4: GBM and LUSC Poisson Distributions of mutant TCEM relative to human proteome and GI Microbiome

Distributions cancer=GBM, facet I=TCEM_I

hg38 maxi_I mut

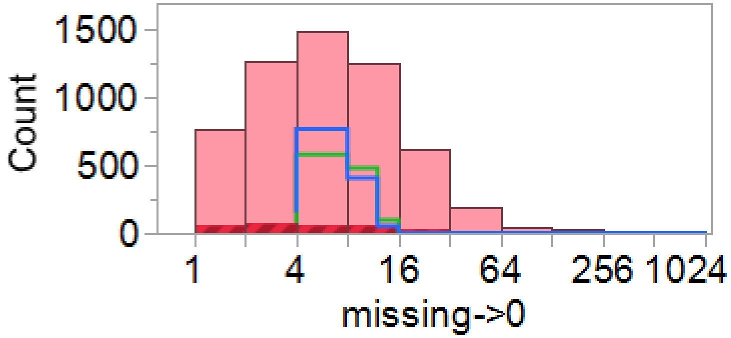

Fitted ZI Poisson Distribution

Parameter		Estimate	Std Error	Lower 95%	Upper 95%
Mean	λ	9.00	0.04	8.92	9.08
Zero Inflation	π	0.10	0.00	0.10	0.11

Measures	
-2*LogLikelihood	80654.80
AICc	80658.80
BIC	80672.26

N TCEM_I (4,5,6,7,8) GI mut

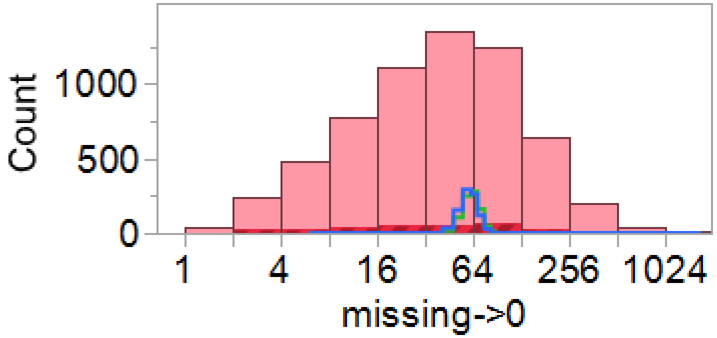

Fitted ZI Poisson Distribution

Parameter		Estimate	Std Error	Lower 95%	Upper 95%
Mean	λ	65.63	0.10	65.43	65.84
Zero Inflation	π	0.03	0.00	0.03	0.04

Measures	
-2*LogLikelihood	450773.28
AICc	450777.28
BIC	450790.75

Distributions cancer=LUSC, facet I=TCEM_I

hg38 maxi_I mut

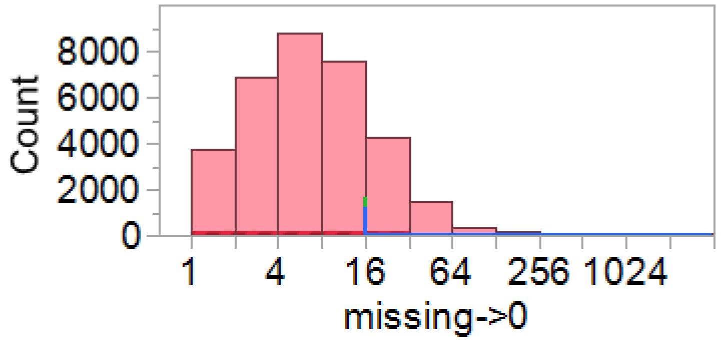

Fitted ZI Poisson Distribution

Parameter		Estimate	Std Error	Lower 95%	Upper 95%
Mean	λ	11.17	0.02	11.14	11.21
Zero Inflation	π	0.09	0.00	0.09	0.09

Measures	
-2*LogLikelihood	761414.69
AICc	761418.69
BIC	761435.67

N TCEM_I (4,5,6,7,8) GI mut

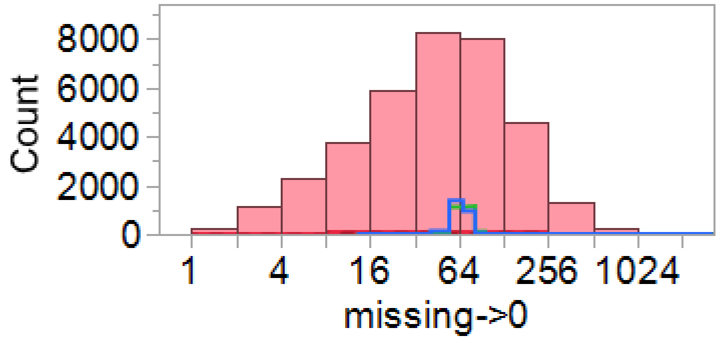

Fitted ZI Poisson Distribution

Parameter		Estimate	Std Error	Lower 95%	Upper 95%
Mean	λ	74.57	0.05	74.48	74.66
Zero Inflation	π	0.03	0.00	0.03	0.03

Measures	
-2*LogLikelihood	2837352.4
AICc	2837356.4
BIC	2837373.3

Distributions cancer=GBM, facet IIa=TCEM_IIa

hg38 maxi_IIa mut

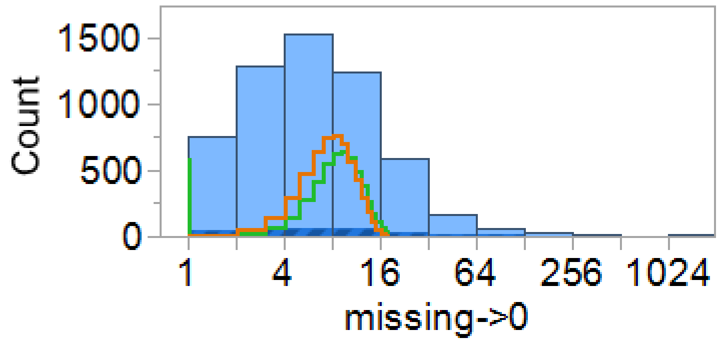

Fitted ZI Poisson Distribution

Parameter		Estimate	Std Error	Lower 95%	Upper 95%
Mean	λ	9.18	0.04	9.10	9.26
Zero Inflation	π	0.11	0.00	0.10	0.12

Measures	
-2*LogLikelihood	90,581.75
AICc	90,585.76
BIC	90,599.21

N TCEM_IIa (2,3,5,7,8) GI mut

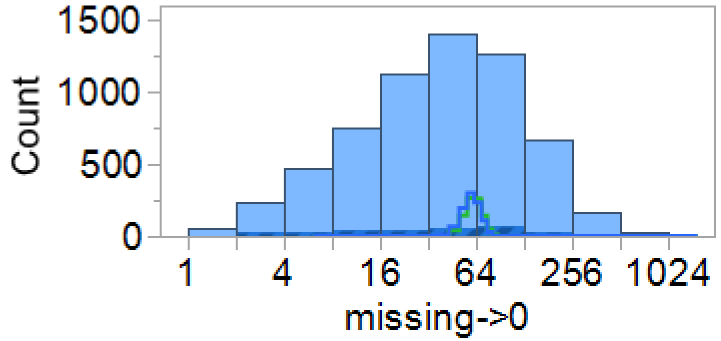

Fitted ZI Poisson Distribution

Parameter		Estimate	Std Error	Lower 95%	Upper 95%
Mean	λ	64.81	0.10	64.60	65.01
Zero Inflation	π	0.03	0.00	0.03	0.04

Measures	
-2*LogLikelihood	428649.72
AICc	428653.72
BIC	428667.18

Distributions cancer=LUSC, facet IIa=TCEM_IIa

hg38 maxi_IIa mut

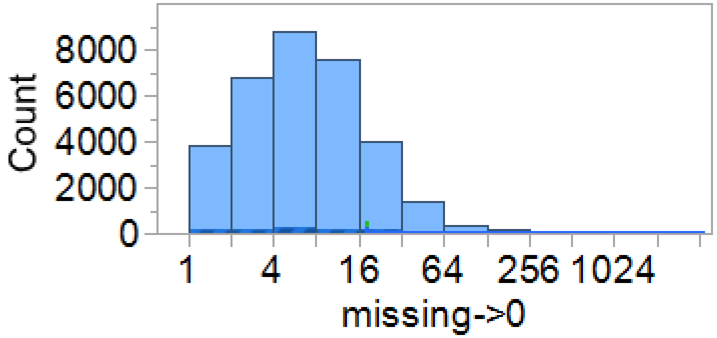

Fitted ZI Poisson Distribution

Parameter		Estimate	Std Error	Lower 95%	Upper 95%
Mean	λ	10.65	0.02	10.61	10.68
Zero Inflation	π	0.09	0.00	0.09	0.09

Measures	
-2*LogLikelihood	673114.40
AICc	673118.40
BIC	673135.37

N TCEM_IIa (2,3,5,7,8) GI mut

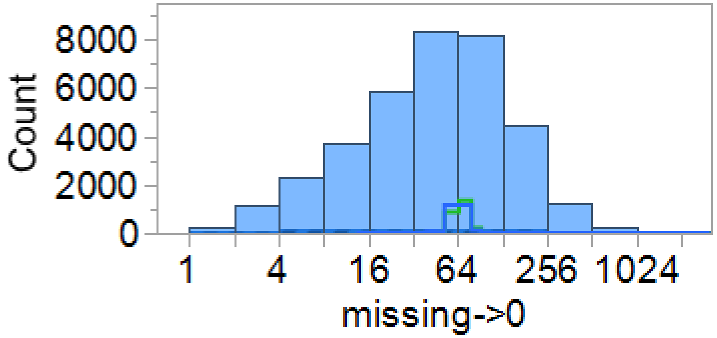

Fitted ZI Poisson Distribution

Parameter		Estimate	Std Error	Lower 95%	Upper 95%
Mean	λ	73.58	0.05	73.49	73.67
Zero Inflation	π	0.03	0.00	0.03	0.03

Measures	
-2*LogLikelihood	2722851.2
AICc	2722855.2
BIC	2722872.2

Supplemental Table 1: Microbiome organisms in reference set.

Gastrointestinal microbiome bacterial proteomes were assembled from the NIH Human Microbiome Project Reference Genomes database (www.hmpdacc.org/HMRGD).

**GI microbiome**
Anaerobaculum hydrogeniformans ATCC BAA-1850
Anaerostipes caccae DSM 14662
Anaerostipes sp. 3 2 56FAA
Bacteroides cellulosilyticus DSM 14838
Bacteroides clarus YIT 12056
Bacteroides eggerthii DSM 20697
Bacteroides sp. 3 1 19
Bacteroides sp. D22
Bacteroides xylanisolvens SD CC 1b
Bacteroides xylanisolvens SD CC 2a
Bifidobacterium breve DSM 20213 = JCM 1192
Bifidobacterium sp. 12 1 47BFAA
Citrobacter youngae ATCC 29220
Clostridiales butyrate-prod
Clostridiales butyrate-producing bacter
Clostridiales butyrate-producing bacterium SS
Clostridiales butyrate-producing bacterium SSC/2
Clostridium sp. M62/1
Clostridium sp. SS2/1
Coprobacillus sp. 8 2 54BFAA
Coprococcus sp. HPP0074
Corynebacterium sp. HFH0082
Edwardsiella tarda ATCC 23685
Enterobacter cancerogenus ATCC 35316
Enterococcus faecalis TX2134
Erysipelotrichaceae bacterium 21 3
Escherichia coli 4 1 47FAA
Escherichia coli MS 60-1
Escherichia coli MS 69-1
Escherichia coli MS 78-1
Escherichia coli MS 84-1
Eubacterium rectale M104/1
Faecalibacterium prausnitzii M21/2
Fusobacterium mortiferum ATCC 9817
Fusobacterium varium ATCC 27725
Hafnia alvei ATCC 51873
Helicobacter pylori GAM101Biv
Helicobacter pylori GAM246Ai
Helicobacter pylori GAM252T

Helicobacter pylori GAM83Bi
Helicobacter pylori GAM93Bi
Helicobacter pylori GAM96Ai
Helicobacter pylori HP116Bi
Helicobacter pylori HP250BFiii
Helicobacter pylori HP250BSi
Klebsiella pneumoniae subsp. pneumoniae WGLW3
Lachnospiraceae bacterium 2 1 58FAA
Lachnospiraceae bacterium 3 1 57FAA CT1
Lachnospiraceae bacterium 5 1 57FAA
Lachnospiraceae bacterium 5 1 63FAA
Lachnospiraceae bacterium 7 1 58FAA
Lactobacillus reuteri MM4-1A
Lactobacillus reuteri SD2112
Listeria innocua ATCC 33091
Megamonas hypermegale ART12/1
Methanobrevibacter smithii DSM 2375
Paraprevotella xylaniphila YIT 11841
Phascolarctobacterium succinatutens YIT 12067
Prevotella oralis HGA0225
Roseburia intestinalis M50/1
Roseburia intestinalis XB6B4
Ruminococcus obeum A2-162
Ruminococcus torques L2-14
Succinatimonas hippei YIT 12066
Sutterella wadsworthensis 2 1 59BFAA
Veillonella sp. 6 1 27
Weissella paramesenteroides ATCC 33313

P40238	MPL	71	Oncogene	Q86U86	PBRM1	193	TSG
Q99836	MYD88	36	Oncogene	A0A0D9SGE8	PHF6	50	TSG
Q16236	NFE2L2	99	Oncogene	P27986	PIK3R1	121	TSG
P01111	NRAS	55	Oncogene	A0A3B3IU23	PRDM1	152	TSG
P16234	PDGFRA	290	Oncogene	Q13635	PTCH1	209	TSG
P42336	PIK3CA	267	Oncogene	P60484	PTEN	292	TSG
P30153	PPP2R1A	121	Oncogene	P06400	RB1	152	TSG
Q06124	PTPN11	101	Oncogene	Q68DV7	RNF43	121	TSG
P07949	RET	209	Oncogene	Q01196_8	RUNX1	84	TSG
Q9Y6X0	SETBP1	322	Oncogene	Q9BYW2	SETD2	323	TSG
O75533	SF3B1	207	Oncogene	Q15796	SMAD2	86	TSG
Q99835	SMO	119	Oncogene	Q13485	SMAD4	160	TSG
O43791	SPOP	92	Oncogene	G5E975	SMARCB1	56	TSG
Q01130	SRSF2	34	Oncogene	O15524	SOCS1	25	TSG
P16473	TSHR	140	Oncogene	P48436	SOX9	98	TSG
Q01081	U2AF1	5	Oncogene	Q8N3U4_2	STAG2	209	TSG
P36896_4	ACVR1B	106	TSG	Q15831	STK11	62	TSG
Q5JTC6	AMER1	253	TSG	Q6N021	TET2	163	TSG
P25054	APC	423	TSG	P21580	TNFAIP3	103	TSG
O14497	ARID1A	294	TSG	P04637	TP53	471	TSG
Q8NFD5	ARID1B	12	TSG	Q6Q0C0	TRAF7	86	TSG
Q8NFD5_3	ARID1B	243	TSG	Q92574	TSC1	128	TSG
Q68CP9	ARID2	261	TSG	P40337	VHL	75	TSG
Q8IXJ9	ASXL1	208	TSG	P19544_7	WT1	98	TSG

Supplemental Table 3: GBM and LUSC TCGA case numbers

GBM	LUSC
TCGA_case_id	*TCGA_case_id*
TCGA-02-2483	TCGA-18-3417
TCGA-02-2485	TCGA-18-4083
TCGA-06-0145	TCGA-21-1070
TCGA-06-0152	TCGA-21-1083
TCGA-06-0155	TCGA-22-1016
TCGA-06-0157	TCGA-22-4601
TCGA-06-0185	TCGA-22-5485
TCGA-06-0188	TCGA-22-A5C4
TCGA-06-0214	TCGA-43-2578
TCGA-06-0686	TCGA-43-8116
TCGA-06-0744	TCGA-56-7730
TCGA-06-0745	TCGA-56-8504
TCGA-06-0877	TCGA-56-8624
TCGA-06-0881	TCGA-56-8625
TCGA-06-2557	TCGA-60-2714
TCGA-06-2570	TCGA-66-2766
TCGA-08-0386	TCGA-66-2782
TCGA-14-0786	TCGA-68-A59J
TCGA-14-2554	TCGA-77-6842
TCGA-19-2620	TCGA-77-8133
TCGA-19-2624	TCGA-77-8139
TCGA-19-2629	TCGA-77-8140
TCGA-19-5960	TCGA-77-A5GB
TCGA-26-5132	TCGA-85-8071
TCGA-26-5135	TCGA-85-8353
TCGA-27-2523	TCGA-85-8580
TCGA-27-2528	TCGA-98-8023
TCGA-28-5218	TCGA-98-A53A
TCGA-32-1970	TCGA-98-A53I
TCGA-32-1980	TCGA-98-A53J
TCGA-41-5651	
